# Brain Cell Type Specific Gene Expression and Co-expression Network Architectures

**DOI:** 10.1038/s41598-018-27293-5

**Published:** 2018-06-11

**Authors:** Andrew T. McKenzie, Minghui Wang, Mads E. Hauberg, John F. Fullard, Alexey Kozlenkov, Alexandra Keenan, Yasmin L. Hurd, Stella Dracheva, Patrizia Casaccia, Panos Roussos, Bin Zhang

**Affiliations:** 1 Department of Genetics and Genomic Sciences, Icahn School of Medicine at Mount Sinai, New York, NY 10029 USA; 2 Icahn Institute of Genomics and Multiscale Biology, Icahn School of Medicine at Mount Sinai, New York, NY 10029 USA; 3 Medical Scientist Training Program, Icahn School of Medicine at Mount Sinai, New York, NY 10029 USA; 4 Friedman Brain Institute, Icahn School of Medicine at Mount Sinai, New York, NY 10029 USA; 5 iPSYCH, The Lundbeck Foundation Initiative for Integrative Psychiatric Research, Aarhus, 8000 Denmark; 6 Department of Biomedicine, Aarhus University, Aarhus, 8000 Denmark; 7 Department of Psychiatry, Icahn School of Medicine at Mount Sinai, New York, NY USA; 8 Mental Illness Research, Education, and Clinical Center (VISN 2), James J. Peters VA Medical Center, Bronx, NY USA; 9 Fishberg Department of Neuroscience, Icahn School of Medicine at Mount Sinai, New York, NY 10029 USA; 10 Neuroscience Initiative, The City University of New York, Advanced Science Research Center, 85 St. Nicholas Terrace, New York, NY 10031 USA

**Keywords:** Network models, Network models

## Abstract

Elucidating brain cell type specific gene expression patterns is critical towards a better understanding of how cell-cell communications may influence brain functions and dysfunctions. We set out to compare and contrast five human and murine cell type-specific transcriptome-wide RNA expression data sets that were generated within the past several years. We defined three measures of brain cell type-relative expression including specificity, enrichment, and absolute expression and identified corresponding consensus brain cell “signatures,” which were well conserved across data sets. We validated that the relative expression of top cell type markers are associated with proxies for cell type proportions in bulk RNA expression data from postmortem human brain samples. We further validated novel marker genes using an orthogonal ATAC-seq dataset. We performed multiscale coexpression network analysis of the single cell data sets and identified robust cell-specific gene modules. To facilitate the use of the cell type-specific genes for cell type proportion estimation and deconvolution from bulk brain gene expression data, we developed an R package, BRETIGEA. In summary, we identified a set of novel brain cell consensus signatures and robust networks from the integration of multiple datasets and therefore transcend limitations related to technical issues characteristic of each individual study.

## Introduction

Interactions among multiple cell types orchestrate the structures and functions of all animal tissues, including the mammalian brain. Distinct cell types in the brain play different and specialized roles in electrical signaling^1,2^, metabolic coupling^3^, axonal ensheathing^4^, regulation of blood flow^5^, and immune surveillance^6,7^. These cell types belong to distinct lineages and are developmentally specified through an integrated transcriptional and epigenetic control of cell differentiation and gene expression^8,9^. A conclusive number of distinct cell types in the mammalian brain cannot be provided without a certain level of uncertainty related to the goals of any given analysis, and is profoundly affected by the sensitivity and specificity of the technology used for cell classification. In bulk brain tissue, gene expression experiments have highlighted cell type composition based on the expression value of markers for five major cell types: neurons, astrocytes, oligodendrocytes, microglia, and endothelial cells^10^. However, within the neuronal population, depending on the source, it has been reported that approximately 50–250 neuronal sub-cell types^11–13^ exist. Similarly, within other lineages, many other cell types have been classified as separate entities, including oligodendrocyte precursor cells (also known as NG2 cells), ependymal cells, smooth muscle cells, and pericytes^14^.

Over the past few years, a series of comprehensive RNA-seq experiments in different brain cell types have been published in humans^15,16^ and mice^17–20^. Some of these experiments have profiled gene expression of cell populations isolated through immunopanning procedures^15,17^. Immunopanning involves immunoprecipitation of particular cell types in cell culture plates, based on selection for an antibody adsorbed to the plate surface^21^. As such, the analysis of currently available data has to take into consideration the limitation of the cell-type isolation procedures, which often included a series of positive and negative selections with pre-defined cell type-specific markers. Others studies have performed RNA profiling of single cells with microfluidics devices and used clustering methods to identify cell types from the resulting RNA expression profiles^16,18,19^. The devices used for single cell RNA sequencing (scRNA-seq) often select cells based on size or via encapsulation in a droplet^22^ and involve the creation of a cDNA library from the transcriptome from a theoretical maximum of one cell. Single cell experiments capture a wider range of cell types than in immunopanning, which reduces bias but acts to increase the variance of the resulting cell type signatures, thus requiring larger sample sizes for analysis. This larger sample size in scRNA-seq, in turn, allows investigators to interrogate the correlation space through network analysis of the interactions among genes^23,24^. However, to the best of our knowledge, when these methods have been applied to brain scRNA-seq data, they have not used a multiscale approach that allows for identification of overlapping gene modules as well as individual gene-gene interactions, as can be performed by MEGENA (Multiscale Embedded Gene Co-expression Network Analysis)^25^.

Previous studies have analyzed brain cell type-specific expression signatures using microarray or RNA-seq in mice^26,27^. However, the existing studies have been mainly based on individual datasets, and are, therefore, subject to systematic noise, including sampling bias due to sample collection or preparation technique, as well as stochastic gene expression. As an increasing number of RNA-seq cell type-specific transcriptomic experiments have become available for both human and mouse, it is desirable to conduct a comprehensive meta-analysis of brain cell type gene signatures. In this manuscript, we first systematically evaluate cell type-specific RNA expression patterns identified in five of these RNA-seq studies^15–19^. The six cell types that we set out to compare are: astrocytes, endothelial cells, microglia, neurons, oligodendrocytes, and oligodendrocyte precursor cells (OPCs). We defined three criteria of ascription of cell type-associated gene expression: specificity, which measures whether a gene is expressed in only one cell type; enrichment, which measures whether a gene tends to have higher expression in one cell type compared to all others; and absolute expression, which measures whether a gene tends to have high expression in a given cell type, irrespective of its expression levels in other cell types. We showed that cell type enrichment patterns in the brain have high overall conservation in pairwise and global comparisons based on all three measures. Next, we interrogated marker genes most specific to each of the cell types, and found that they were significantly associated with text mining results in PubMed (https://www.ncbi.nlm.nih.gov/pubmed) searches, and that their aggregate expression patterns were well-correlated with immunohistochemistry-level data from the same brain samples. As a resource to the community, we release these gene signatures and functions for leveraging them in an R package, BRETIGEA (BRain cEll Type specIfic Gene Expression Analysis), which we validated on a postmortem brain gene expression data set. Finally, we perform network analysis on the scRNA-seq data sets using the multiscale network modeling approach MEGENA, showing that there is conservation of modules both within and across cell types. Taken together, our results establish fundamental cell type-associated gene expression patterns in the brain that can be used for many applications, including cell type enrichment analysis of differential expression signatures, deconvolution of relative cell type proportions from bulk RNA-seq data, and facilitating the interpretation of network analyses in scRNA-seq experiments.

## Results

### Identification and comparison of cell type-associated expression across multiple data sets

**Figure 1:**
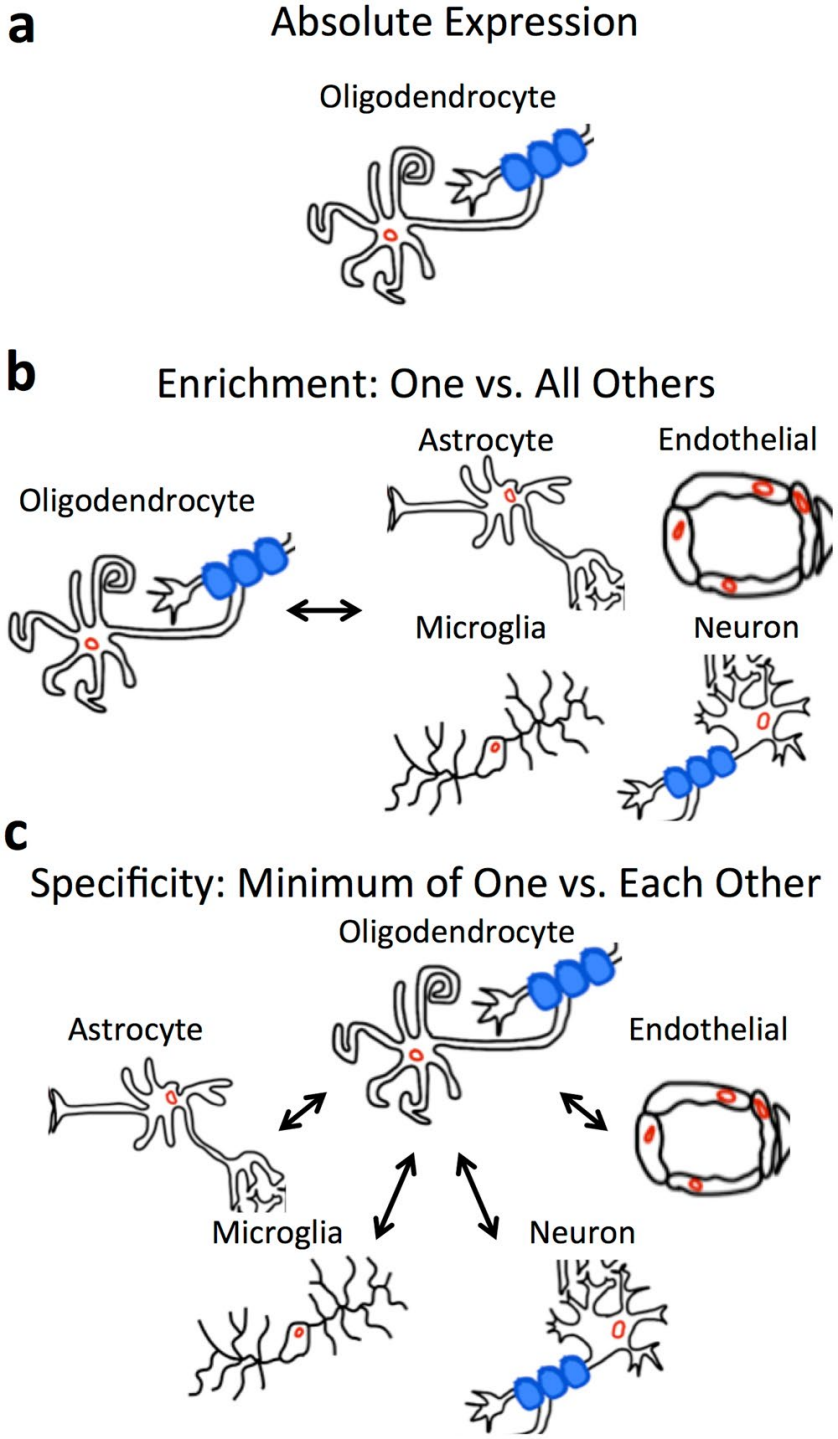
Explanation of the three cell type associated measures. Diagrams showing the three cell type-associated measures used in this study. (**a**) Cell type absolute expression simply measures the relative expression of each gene in each cell type, irrespective of the expression of that gene in other cell types. (**b)** Cell type enrichment measures the expression of each gene relative to the expression of that gene in all other cell types. With this measure, a gene could have relatively high expression in two cell types, and be relatively enriched in each of them compared to all other cell types. (**c)** Cell type specificity measures the expression of each gene relative to the highest expression of that gene in all other cell types. This measure requires that the expression of the gene is only high in one cell type; therefore, we call it “specific.”

**Figure 2:**
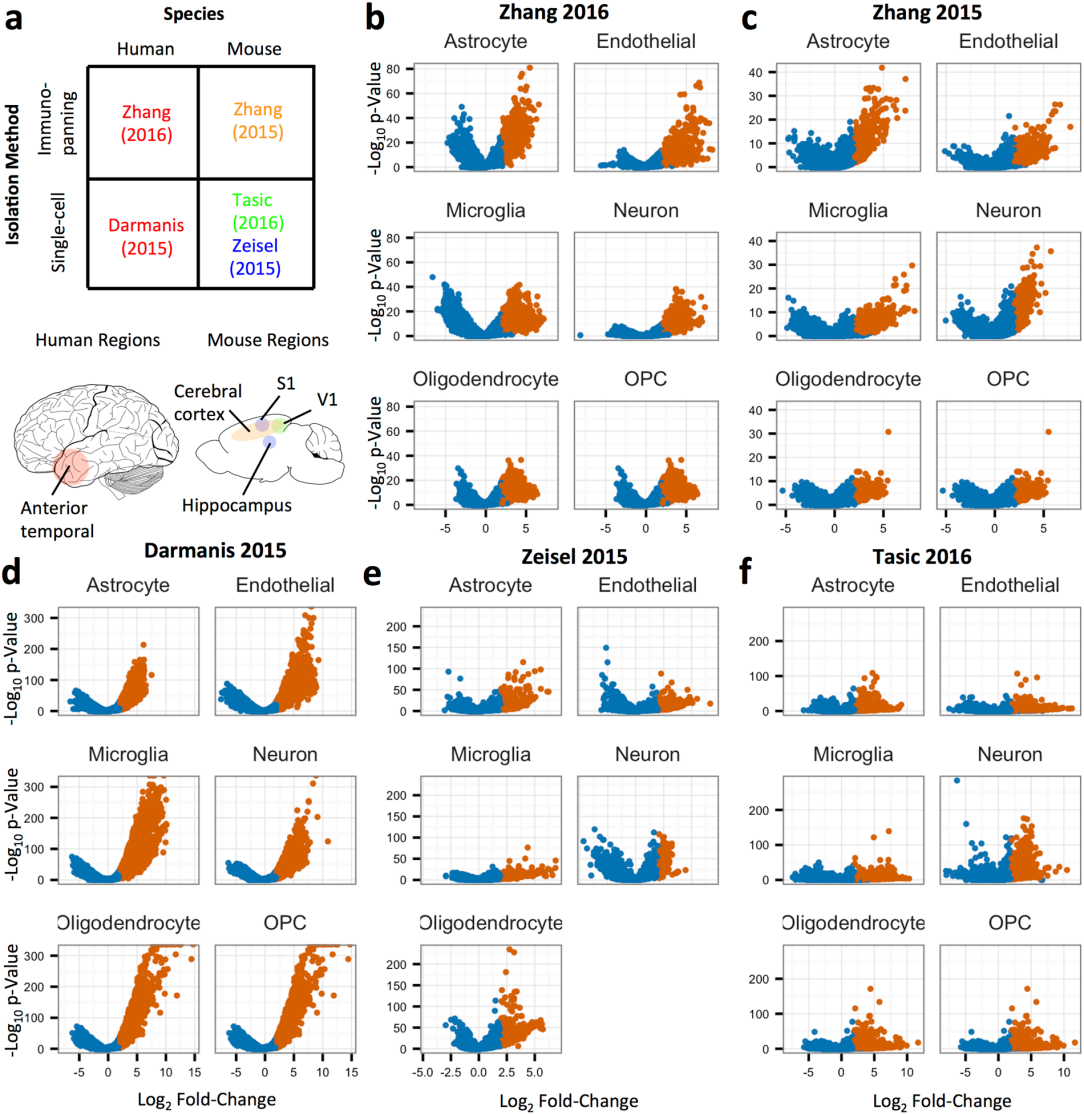
Differential expression volcano plots across cell types and data sets for cell type enrichment. (**a**) Summary of the five data sets used in this study indicating the region in which the cell types were isolated from in both the human (left) and mouse (right) brains. (**b–f)**: Volcano plots show the cell type enrichment differential expression calculation for each cell type in each data set (**b** = Darmanis *et al*. **c** = Zhang *et al*., **d** = Zhang *et al*., **e** = Zeisel *et al*. **f** = Tasic *et al*.). In each small multiple, the x-axis refers to the log2 fold-change of the gene in the cell type of interest compared to the reference cell set, while the y-axis shows the −log_10_ Benjamini-Hochberg adjusted p-value of that comparison, calculated using edgeR. Genes with adjusted p-values < 0.05 and log fold-change > 2 are colored orange. OPC = Oligodendrocyte Precursor Cell.

The goal of this study is to define and explore cell type-specific gene expression patterns via a meta-analysis of brain cell type RNA expression data from five studies with publically available RNA-sequencing reads^15–19^ (Table 1). Towards this end, we downloaded and reprocessed the raw sequencing reads with a unified pipeline, and used three separate metrics to calculate cell type-relative expression specificity, enrichment, and absolute expression data from each data set. We defined *cell type specificity* as the minimum fold change in expression between the cell type of interest and each of the other cells; *cell type enrichment*, as the fold change in expression when comparing the cell type of interest to all of the other cells at once; and *cell type absolute expression*, as the expression in a particular cell type irrespective of that in other cell types (Fig. 1). Consistent with the strong developmental and epigenetic effect of cell type status on gene expression, we identified a strong signal for all three measures in all cell types and data sets. For example, we found a large number of significantly enriched genes in all cell types in every dataset (Fig. 2), with a mean of 1,430 (SEM = 197) genes significantly enriched in the cell type of interest at cutoffs of fold-change > 4 and Benjamini-Hochberg (BH)-adjusted p-value < 0.05.

**Table 1:** Summary of RNA expression data sets used in this analysis.

Data Set	Species	Data Type	GEO accession	# AST	# END	# MIC	# NEU	# MOL	# OPC	Covariates
Darmanis	Human	Single-cell	GSE67835	62	20	16	131	38	18	Total Features
Zhang (2016)	Human	Cell population	GSE73721	12	2	3	1	5	—	—
Zhang (2015)	Mouse	Cell population	GSE52564	2	2	2	2	2	2	—
Zeisel	Mouse	Single-cell	GSE60361	129	137	33	1519	484	—	Total Features, Tissue, Age
Tasic	Mouse	Single-cell	GSE71585	50	21	22	1465	42	24	Total Features

The sample size for each cell type, as well as the covariates included in the differential expression model, for each of the data sets used in this meta-analysis. Note that these numbers are calculated subsequent to cleaning and combining of cell populations originally annotated in each of the data sets, as described in the Methods. A dash indicates that a cell type was not present in that data set. AST = astrocyte, END = endothelial cell, MIC = microglia, NEU = neuron, MOL = mature oligodendrocyte, OPC = oligodendrocyte precursor cell.

**Figure 3:**
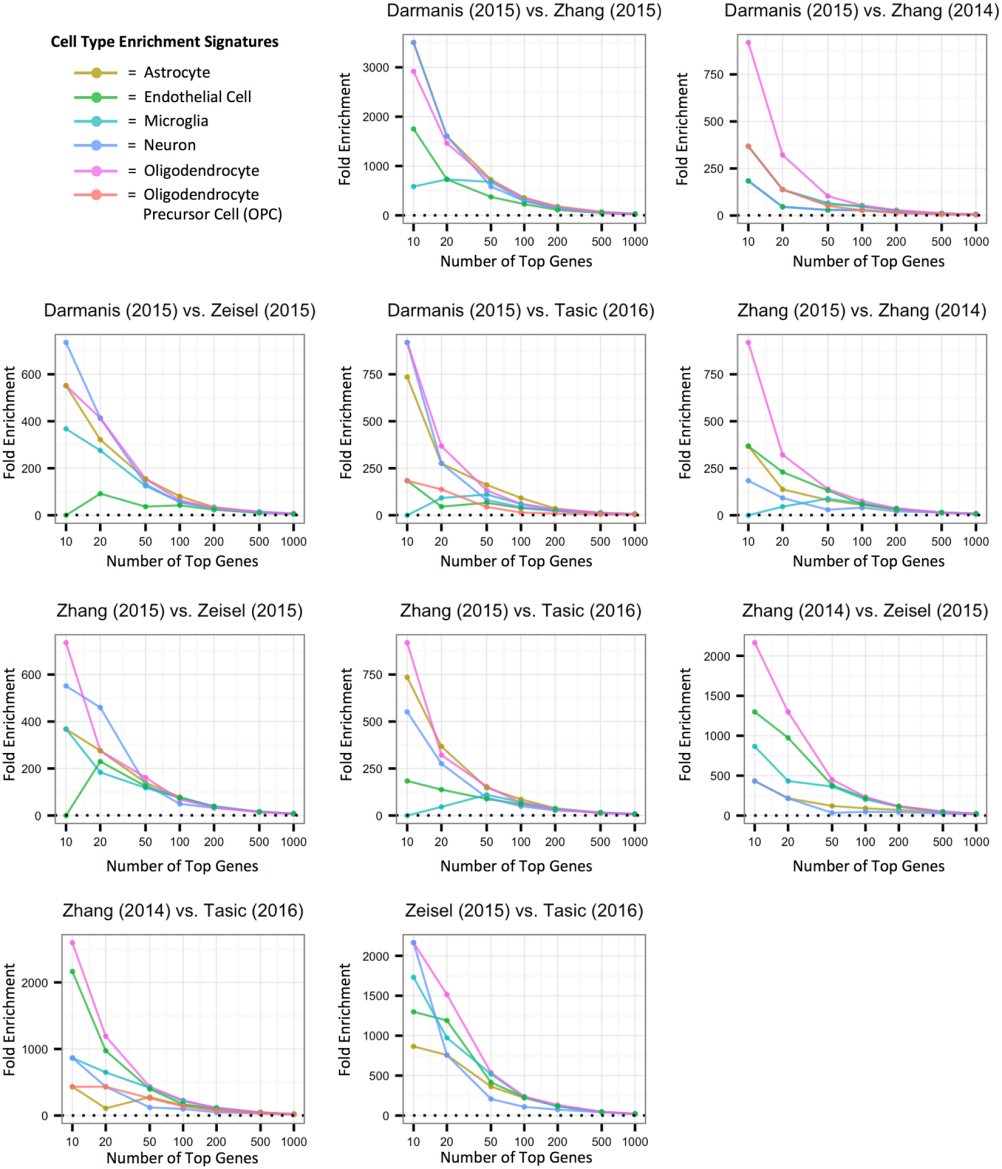
Pairwise data set comparison of cell type enrichment rankings for each cell type. Plots of the fold-enrichment of the intersections of genes ranked in the top n genes (where n = 10, 20, 50, 100, 200, 500, 1000) between pairs of data sets for the cell type enrichment measure. The data sets were merged to only include gene symbols common to both prior to calculating the fold enrichment score. A fold enrichment of 0 indicates that no genes were found in the intersection of those two sets of top genes.

We next measured the pairwise overlap of the top cell type specific, cell type enriched, and cell type expressed genes at various top gene cutoffs from n = 10 to n = 1000 by calculating their fold enrichment in each other in pairwise signature comparisons (Fig. 3, Supplemental Figs 1, 2). In order to summarize these pairwise intersections, we calculated the average pairwise fold enrichment across data sets for each of the three measures (Supplemental Fig. 3A,C,E). We found that the top 10 most enriched oligodendrocyte genes had a significantly higher average number of intersections across pairs of data sets than the top 10 most enriched microglia genes (Cohen’s d = 1.66, p = 4.4e-6). Consistent with this, in global comparisons of the top 10 genes in the cell type enrichment signatures, 3 genes (*PLP1*, *UGT8*, and *ERMN*) were shared among all five of the oligodendrocyte signatures, while none were shared among all five of the microglial signatures (Supplemental Fig. 3B,D,F). However, when we used the top gene cutoffs of 50 genes and above, we detected a strong global overlap between all cell type-relative measures in all cell types (Supplemental Fig. 3B,D,F). For example, when considering the 100 top genes most enriched in a cell type, there was a highly significant enrichment in astrocytes (Fold Enrichment (FE) = 1.4e8, p = 4.2e-103), endothelial cells (FE = 1.2e8, p = 2.8e-86), microglia (FE = 1.2e8, p = 2e-86), neurons (FE = 4.2e7, p = 3.7e-30), and oligodendrocytes (FE = 2e8, p = 6.4e-163) across the five data sets. The intersection of the top 100 genes most enriched in OPCs in the three data sets with OPC signatures available was also highly significant (FE = 1628, p = 2.2e-15).

### Ranking genes by their cell type-associated expression across data sets

We next sought to devise a consensus ranking across data sets for all three of the cell type-associated measures. For cell type enrichment and specificity, we used the mean of the fold change across data sets to order genes. For cell type expression, we ranked expression values across data sets and used the median of the ranks to order genes. We present the top 1000 genes for each of the six cell types and three measures based on aggregating the signatures across each of the human, mouse, and combination mouse and human data sets (Supplemental File 1). All six of the genes we identified as the most enriched in each cell type across all data sets have been previously associated with specific cell type: *AQP4* in astrocytes^28^, *APOLD1* in endothelial cells^29^, *CCL4* in microglia^30^, *RELN* in neurons^31^, *PLP1* in oligodendrocytes^32^, and *PDGFRA* in OPCs^33^. Consistent with the similarity of the cell type-enrichment and cell type-specificity measures, the top genes identified were the same between these two measures in all cell types with the exception of microglia, in which *CCL3*, a secreted chemokine that regulates local inflammation levels^34^, was ranked the most specific gene. Notably, *PRNP*, encoding the prion protein, is ranked among the top 200 most expressed genes in all six cell types, and is highly expressed in the brain relative to other tissue types^35^, suggestive of brain-specific regulation.

**Figure 4:**
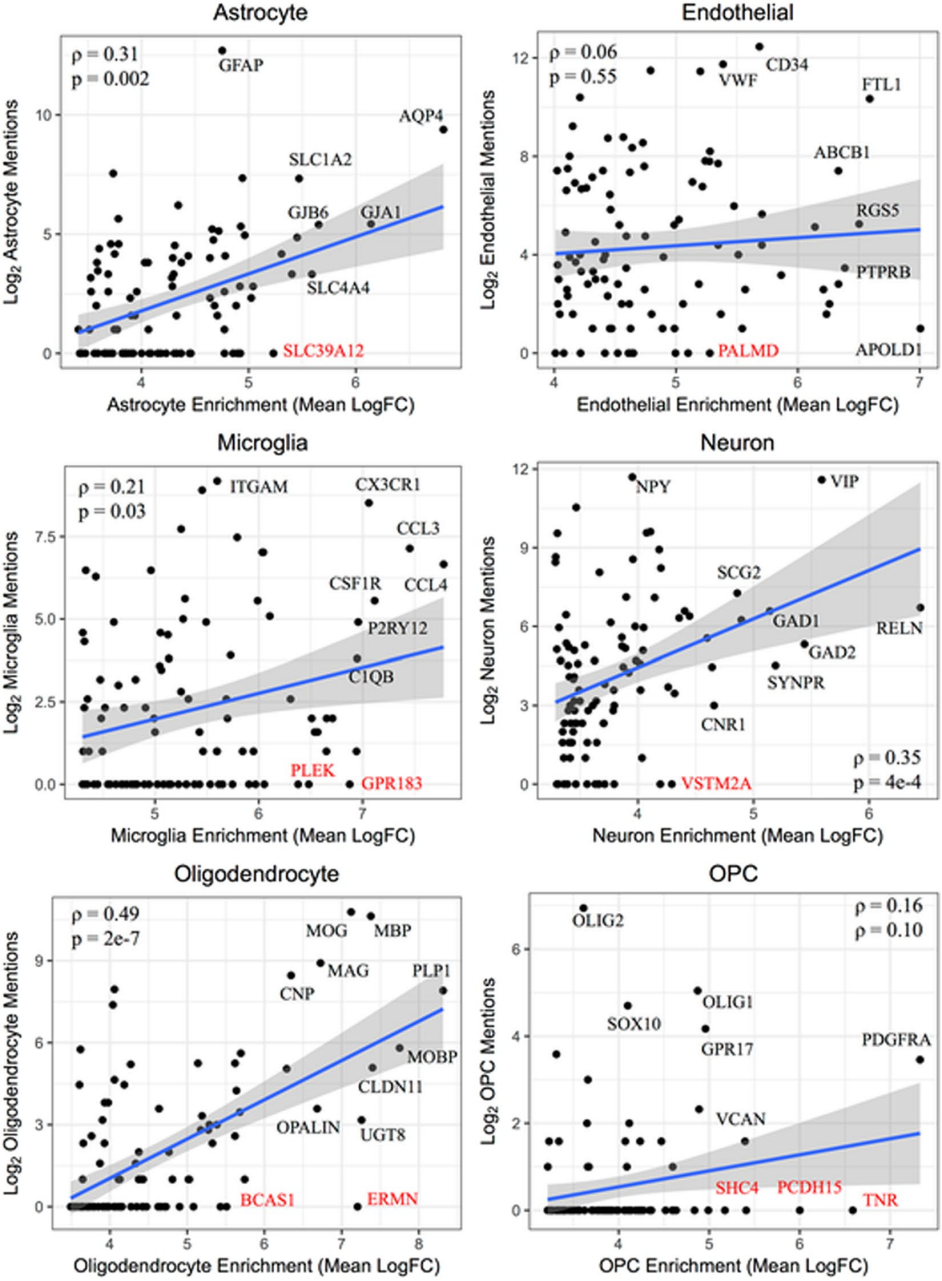
Correlation of genewise cell type enrichment measures with PubMed text mining results within cell types. For each of the six cell types, i.e. the astrocyte (**a**), endothelial cell (**b**), microglia (**c**), neuron (**d**), oligodendrocyte (**e**), and oligodendrocyte precursor cell (**f**), the top 100 gene symbols most enriched in that cell type are plotted against the number of PubMed abstracts that mention both that gene symbol as well as the corresponding cell type. The Spearman correlation between these measures was calculated. Several gene symbols were chosen for highlighting, including gene symbols that have not been mentioned in a PubMed abstract with that cell type to date (labeled red). Note that for oligodendrocyte precursor cells (OPCs), the cell type name used in the PubMed search was “oligodendrocyte precursor.”

Next, we searched PubMed for the combination of gene symbols most enriched in each of the cell types and the word defining that specific cell type (Supplemental File 2). For each cell type, we found the number of PubMed hits found when searching for cell-specific markers compared to the set of all other marker genes. We detected a significant difference for astrocytes (U-test p = 0.003), endothelial cells (p = 2e-14), microglia (p = 1e-9), neurons (6e-5) and oligodendrocytes (9e-4), with a trend identified for the less commonly studied OPCs (p = 0.056; Supplemental Fig. 4). When we restricted this analysis to a correlation within the top 100 most enriched genes for a single cell type, we detected significant rank correlations between our cell type-enrichment data and the text mining results for astrocytes (ρ = 0.31, p = 0.002), microglia (ρ = 0.21, p = 0.035), neurons (ρ = 0.34, p = 4e-4), and oligodendrocytes (ρ = 0.49, p = 2e-7), thus corroborating the difference even among the highest-ranked marker genes for these cell types (Fig. 4). Notably, we detected a no significant relationship for OPCs (ρ = 0.16, p = 0.10) and endothelial cells (ρ = 0.06, p = 0.55). Endothelial cells are also widely studied for their function outside of the brain, where they may have different patterns in gene expression as they have within the brain, which may have introduced noise into the rank correlation result for this cell type. This analysis also allowed us to identify genes strongly enriched in a particular cell type but not described in the literature for their role in that cell type (Fig. 4). For example, we identified *SLC39A12*, a gene encoding a zinc transporter necessary for neural development^36^, as highly enriched in astrocytes across data sets (mean log_2_ fold-change = 5.23), despite no PubMed abstracts mentioning this robust association. Considering the reports of dysregulation of *SLC39A12* in persons with schizophrenia^37,38^, this suggests that the putative dysregulation of zinc processing in schizophrenia may be associated with astrocytes.

### Comparison of top gene rankings among cell type association measures

**Figure 5:**
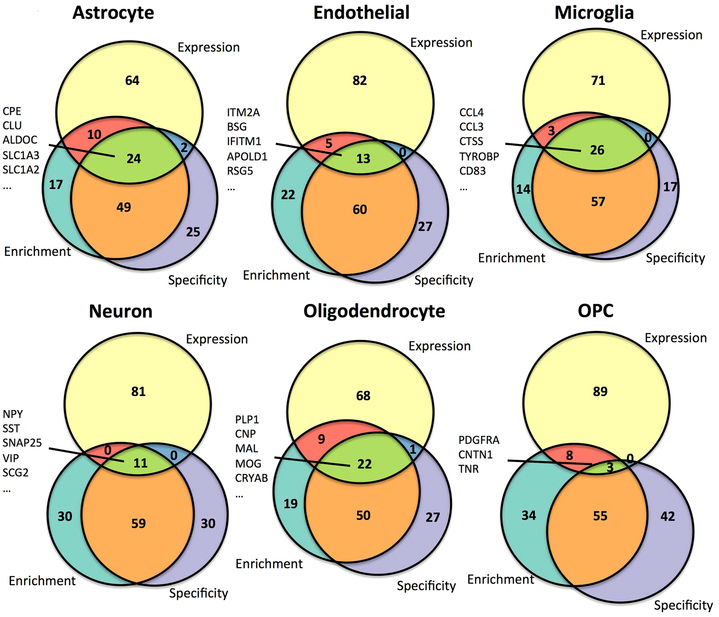
Intersections among the top genes for three cell type associated measures consensus rankings across all cell types. The top 100 genes ranked across both mouse and human data sets for each of the cell type measures are intersected using approximately proportional Venn diagrams for each of the astrocyte (**a**), endothelial cell (**b**), microglia (**c**), neuron (**d**), oligodendrocyte (**e**), and oligodendrocyte precursor cell (**f**) signatures. The Venn diagrams were generated using the R package Vennerable (version 3.0). The 5 genes with the top expression values in each of the cell types that intersect in all three of the top 100 gene sets are listed, with the exception of OPCs, for which all 7 of the genes with intersections between the three measures are listed.

We next sought to compare the top 100 genes as measured by each of the three cell type-associated measures in the consensus signatures created across both humans and mice (Fig. 5). As expected due to the similarity of the cell type-specificity and cell type-enrichment measures, there was a much stronger overlap between cell type-specificity and cell type-enrichment than between either of these measurements and cell type-absolute expression. However, we found that several of the top 100 most expressed genes were also found in the top 100 most enriched and most specific signatures for each cell type, including 26 in microglia (Fig. 5). This finding suggests that a non-trivial portion of the overall RNA content of a particular brain cell type has uniquely high expression in that cell type. Notably, 19 of the top 100 highest-expressed genes in each cell type were shared across all 6 cell types, including *MALAT1*, *ACTB*, *HSPA8*, *FTH1*, *FOS*, *HSP90AB1*, *EEF1A1*, *UBC*, *RPL4*, *RPS11*, and several mitochondrial genes. These pan-cell type high-expression genes and their associated products may be maintaining or mediating processes essential to cellular function across all of the brain cell types.

As an additional measure of the similarity between our three cell type association measures, we calculated Spearman correlations between human and mouse combined gene rankings as defined by each of the three measures for all of the six cell types (Supplemental Figs 5–10). Because the cell type-enrichment and cell type-specificity measures are designed to distinguish between genes with relatively high cell type expression bias in a given cell type, we restricted the correlation analysis to genes ranked in the top 1000 according to one of the measures at a time. As expected from the definitions of each of the three measures, genes with high specificity for one cell type also tended to have high enrichment for that cell type. For example, genes ranked in the top 1000 for specificity in astrocytes tended to have much higher ranks for enrichment in astrocytes as well (ρ = 0.67, p = 7.8e-129). However, these measures were far from perfectly correlated, suggestive of differences in the measures. We also found that genes ranked as highly specific or enriched in a given cell type also tended to have high expression in that cell across all six cell types, consistent with the use of shrinkage estimation in our calculations of cell type-specificity and -enrichment.

### Validating cell type specific markers in bulk RNA expression data

**Figure 6:**
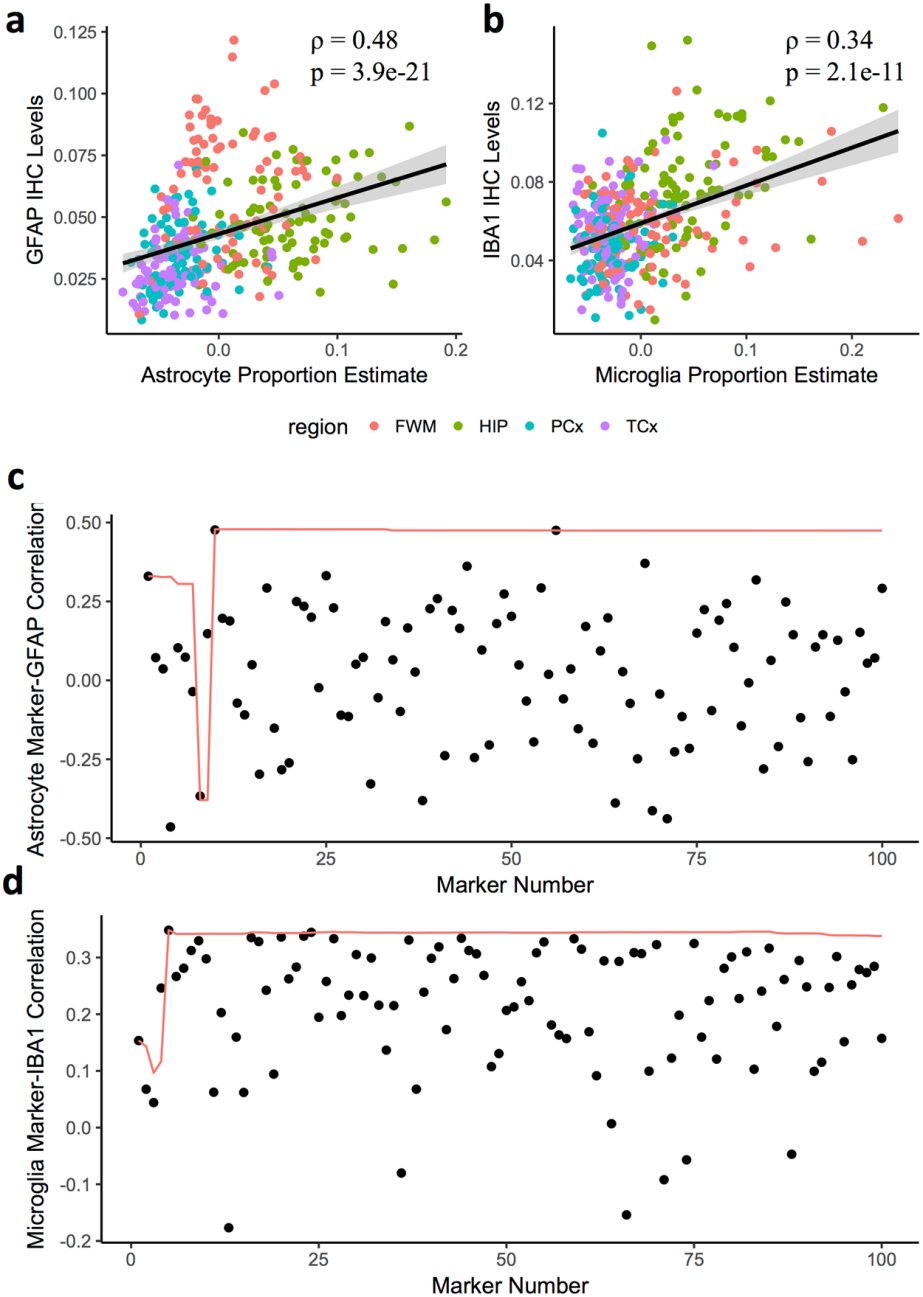
Cell type relative proportion estimates from bulk RNA expression for astrocytes and microglia are associated with IHC quantifications from the Allen Brain Atlas brain bank. (a-b) Scatter plots of the relative cell type proportion estimates, generated using a singular value decomposition (SVD) of the top 50 marker genes for astrocytes (**a**) or microglia (**b**) (x-axis) with the immunohistochemistry (IHC) protein level quantifications for the astrocyte marker GFAP (**a**) or the microglia IBA1 (**b**) (y-axis) in the same donors across the four brain regions in the Allen Brain Atlas Aging, Dementia, and TBI data set. The black line is a result of a linear model fit to the data, while the grey lines represent 95% confidence intervals. (**c**,**d**) Rho values resulting from rank correlations of the RNA expression of the top 100 marker genes individually (black dots) as well as the SVD-based estimate using the cumulative astrocyte (**c**) or microglia (**d**) marker genes up to that marker gene, inclusive (red lines), with the IHC quantifications for GFAP (**c**) or IBA1 (**d**). FWM, frontal white matter; HIP, hippocampus; PCx, parietal cortex; TCx, temporal cortex.

We next sought to validate that our cell type-specific genes (henceforth, marker genes) could be used to estimate the relative proportions of cell types in tissue samples in an independent data set. To do so, we employed matched RNA expression and immunohistochemistry (IHC) quantification data from the frontal white matter, hippocampus, temporal cortex, and parietal cortex in the Allen Brain Atlas Aging, Dementia, and TBI study^39^. We found that our singular value decomposition (SVD)-based estimation for the relative proportion of astrocytes and microglia, based on the RNA expression of the top 50 cell marker genes,, had significant rank correlations with the IHC quantifications for the astrocyte marker GFAP^40^ (rho = 0.48, p = 3.9e-21) and the microglia marker IBA1^41^ (rho = 0.34, p = 2.1e-11), respectively, across all four brain regions (Fig. 6A,B). When we compared these correlations to those found between the individual RNA expression values of the top 100 ranked marker genes for astrocytes and microglia and the IHC quantifications for GFAP and IBA1, we found that the cumulative consensus estimate outperformed the majority of them, thus demonstrating the practical utility of our multiple marker-based approach (Fig. 6C,D). Notably, the sign was switched for the astrocyte proportion estimate that included only the top 8 and 9 human astrocyte marker genes (Fig. 6C), emphasizing the importance of leveraging a larger set of marker genes, which allows for a more robust cell type proportion sign correction. As an exploratory analysis, we also estimated the relative proportion of astrocytes and microglia within (instead of across) each of the four brain regions included in the Allen Brain Atlas Aging, Dementia, and TBI data set (Supplemental Fig. 11). These region-specific correlations have lower power because of diminished sample sizes and decreased variance across brain regions. However, we still identified nominally significant rank correlations between estimated relative astrocyte proportions and IHC quantifications for GFAP in the parietal cortex (ρ = 0.27, p = 0.01) as well as estimated relative microglia proportions and IHC quantifications for IBA1 in the hippocampus (ρ = 0.38, p = 0.0003).

### Validation of novel cell marker genes using ATAC-seq

We screened for potential novel marker genes among the list of top 100 cell type-specific genes for each cell type by searching PubMed. Any marker gene whose gene symbol or synonym coincides with its cell type of interest in at least one PubMed record was considered a possibly known marker gene, and was otherwise considered a potential novel marker gene. The PubMed inquiry was automated by using R package RISmed (v 2.1.6). As of August 10, 2017, we obtained 43, 16, 33, 11, 51, and 88 potential novel marker genes for astrocyte, endothelial, microglia, neuron, oligodendrocyte, and oligodendrocyte precursor (OPC), respectively. While this naïve PubMed query approach was neither an exhaustive or comprehensive means for identifying novel marker genes and hence false positive and false negative were unavoidable, our analysis suggested that a majority (60%) of the top markers were possibly known to be associated with their predicted brain cell type, thus partly validating the present top marker genes. The result also revealed that neuronal cell type markers are more likely to have been studied in the literature while the oligodendrocyte and OPC are less studied.

**Figure 7:**
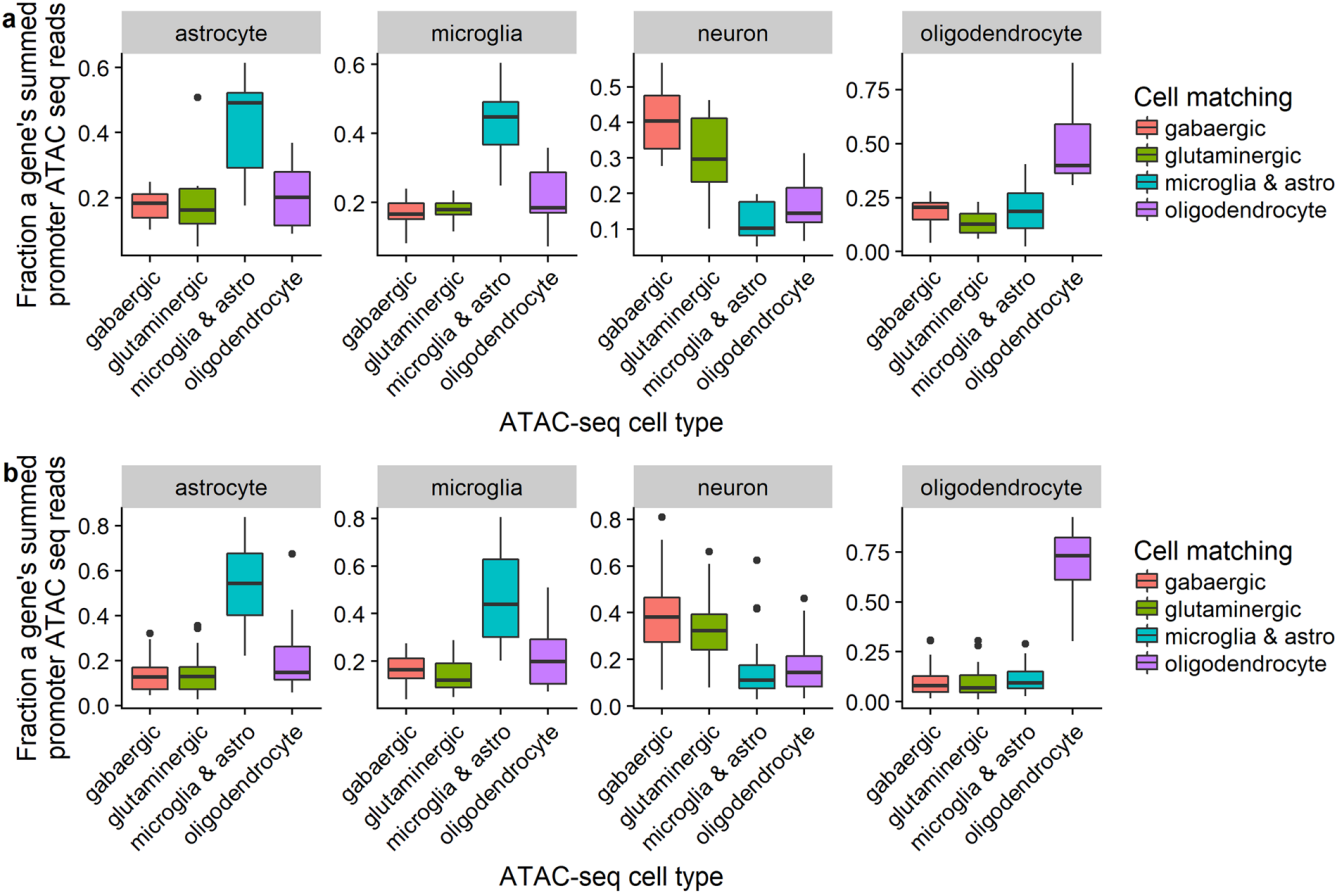
Relative chromatin accessibility in (**a**) novel and (**b**) known brain cell type marker genes by ATAC-seq. Relative chromatin accessibility, as measured by Assay for Transposase-Accessible Chromatin sequencing (ATAC-seq), at the promoters of novel candidate cell marker genes in four different ATAC-seq cell populations from the adult human brain. “Marker” genes were defined as top cell type-specific genes for a particular cell type. In each panel, the boxplots show the distribution of the fraction of ATAC-seq reads regarding novel markers of a particular cell type. Astro, astrocytes.

To further validate our novel marker genes, we examined the signatures of open chromatin at these genes using ATAC-seq data in 4 brain cell types: gabaergic neurons, glutaminergic neurons, oligodendrocytes, and glial cells of microglia/astrocyte type. As there were no matching cell types in the ATAC-seq data for the endothelial and OPC cells, these were excluded from the analysis. In the ATAC-seq data, the number of reads in an identified region of open chromatin correlates with its accessibility. In turn, the number of ATAC-seq reads at a gene’s promoter is likely to be positively correlated with the gene’s expression^42^. When focusing on the top 10 (except in neurons where there were only 8), potential novel markers for each type which had a transcription start site (TSS) overlapping with one or more ATAC-seq peaks, the level of ATAC-seq expression (i.e. normalized peak read counts; see the Supplemental Methods for data processing) at TSS was significantly higher in predicted marker cell types than in other cell types (one-tailed t-test p-value ranged from 5.0 × 10^–4^ to 1.7 × 10^−6^; Fig. 7a). 32 (88.9%) out of the 38 tested novel markers revealed the chromatin at the TSS to be most accessible in the matching ATAC-seq cell type among 4 different cell types measured. Table 2 lists the 32 novel markers validated by ATAC-seq. Moreover, 36 (94.7%) of the tested novel markers showed the highest or the second highest levels of open chromatin at the TSS in the matching ATAC-seq cell type.

**Table 2:** Novel marker genes validated by ATAC-seq.

Cell type	Gene
astrocyte	ADGRV1, CLDN10, ETNPPL, PRSS35, RNF219-AS1, STON2, TPD52L1
microglia	ARHGAP25, ATP8B4, FAM105A, HLA-DPA1, MS4A14, PIK3AP1, SH2B3, TRIB1, UBAC2
neuron	BTBD11, DISP2, GALNTL6, SERTM1, SYT13, VSTM2A, ZMAT4
oligodendrocyte	ANLN, CARNS1, CLCA4, CTNNA3, PAIP2B, QDPR, SLAIN1, SOX2-OT, TMEM144

As a comparison, we also evaluated the enrichment of ATAC-seq expression at the TSS of top ranked known marker genes which were revealed by the present PubMed search. 171 known marker genes had a TSS overlapped with ATAC-seq peaks. Again, the ATAC-seq expression level at the TSS was significantly higher in predicted marker cell types than in other cell types (one-tailed t-test p-value ranged from 1.0 × 10^−10^ to 2.4 × 10^−28^; Fig. 7b). Compared to the same analysis in novel markers, the stronger enrichment and thus smaller t-test P value herein was likely due to an increased power by a larger number of known marker genes tested. Nonetheless, 95.9% (164/171) of the known marker genes showed the highest or the second highest levels of open chromatin at the TSS in the matching ATAC-seq cell type, which was comparable to what we observed from the novel marker genes.

### Functional evaluation of the marker genes using MGI phenotype

To test whether the present brain cell type marker genes were associated with any phenotype, we overlapped the marker genes with MGI phenotype gene set (http://www.informatics.jax.org/) and employed Fisher’s exact test (FET) to search for significant enrichment. Supplemental Fig. 12 and File 3 summarize the MGI phenotype enrichment analysis for the top 500 specificity marker genes in each of the 5 brain cell types, except for OPCs. Two MGI phenotypes, including nervous system (1.3~2.4-Fold Enrichment (FE), FDR-adjusted FET p-value (p) < 0.01) and homeostasis/metabolism (1.2~1.7 FE, p ≤ 0.05) were enriched in marker genes from all the 5 cell types. In addition, behavior/neurological phenotype was enriched in 4 brain cell type markers (1.4~2.5 FE, p ≤ 5 × 10-4), except microglia and OPC. Significant enrichment of nervous system and neurological phenotype in the brain cell type marker genes indicated the critical roles of the markers implicated in normal brain function. Among the other significant phenotypes, immune system was enriched in microglia (3.0 FE, p ≤ 1.7 × 10^−51^) and endothelial (1.9 FE, p ≤ 8.1 × 10^−15^) cell type markers, consistent with the active participation of the two cell types in both innate and adaptive immune responses.

### Identification of robust cell type-associated coexpression networks

**Figure 8:**
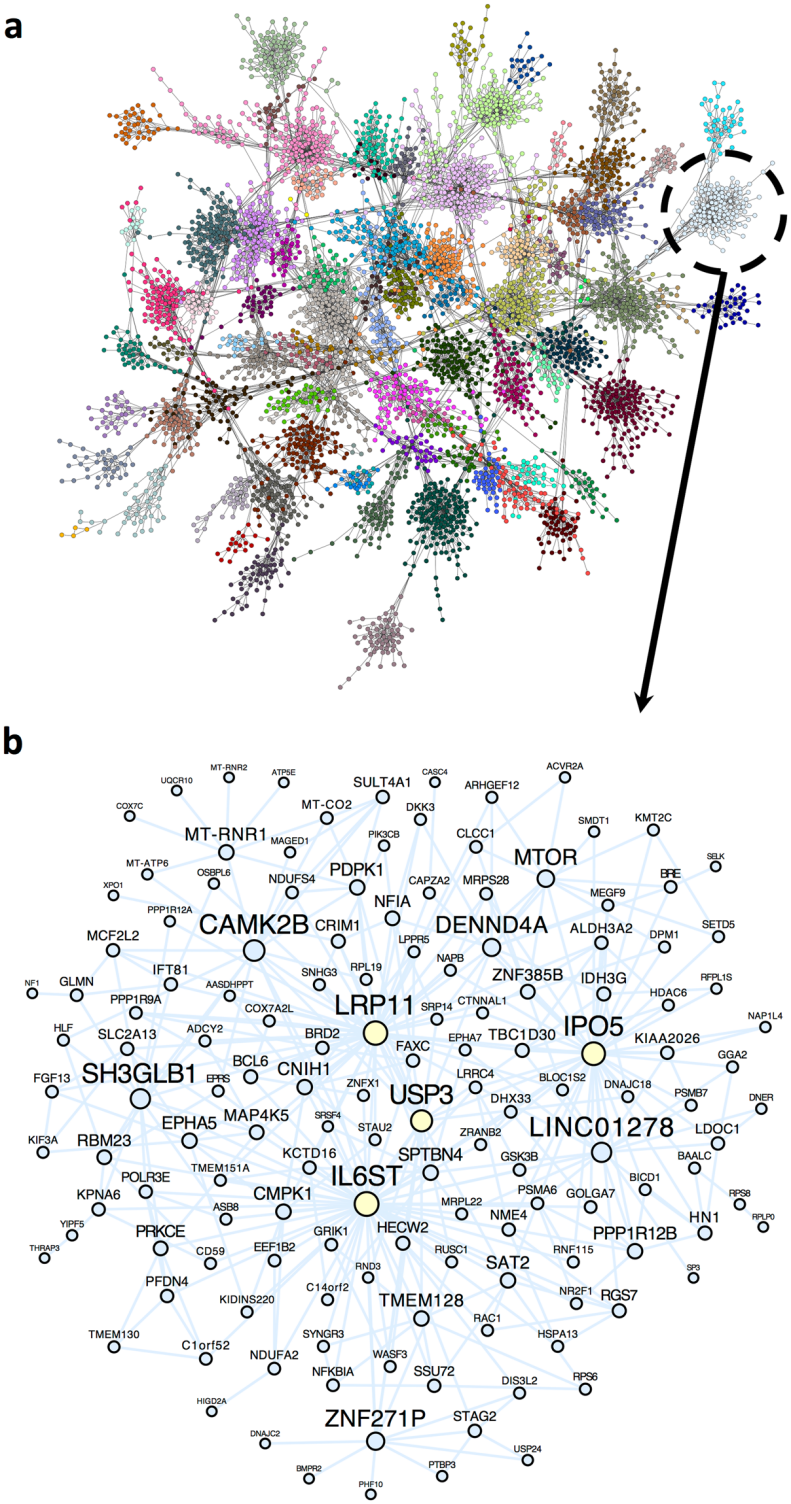
Multiscale networks identified within neurons contain neuron-specific modules. (**a**) Multiscale network modules identified using MEGENA in the Darmanis *et al*. human neuron-annotated RNA-seq samples. Dots represent genes while lines represent network connections, and dots are colored by their presence in one of several modules. **(b)** Zoomed-in gene-level network for the genes in Module #57, which is significantly enriched in the GO term “neurotrophin TRK receptor signaling pathway”.

To interrogate the interactions of genes within each of the human brain cell types, we applied MEGENA, a multiscale clustering algorithm, to RNA-seq data from the human Darmanis *et al*. data set, identifying modules within each cell type and data set that may correspond to particular subcellular compartments, and/or signaling pathways (Supplemental File 4). For example, in the neuron network, we identified more than a hundred modules of coexpressed genes (Fig. 8A). Notably, Module #57 is substantially enriched in neuron-specific genes (FE = 7.3, p = 1.4e-9) and is also enriched in genes associated with the GO term “neurotrophin TRK receptor signaling pathway” (OR = 6.8, p = 1.2e-6) (Fig. 8B; Supplemental Fig. 13). The top hub gene in this module is *IL6ST*, which encodes a transmembrane protein that activates the JAK/STAT pathway and has been associated with neuron growth in human neuroprogenitor cells^43^. In networks associated with the other cell types, we also found modules enriched in genes associated with cell type marker enrichments and GO terms suggesting cell type-specific activity, including “glutamate secretion” in astrocytes^44^ (Module #19, Odds Ratio (OR) = 24, p = 0.0003, Supplemental Fig. 14), “regulation of platelet-derived growth factor receptor signaling pathway” in endothelial cells^45^ (Module #24, OR = 261, p = 1.8e-5, Supplemental Fig. 15), “interleukin-6 receptor activity” in microglia^46^ (Module #38, OR = Inf, p = 0.0003, Supplemental Fig. 16), “glucose import” in oligodendrocytes^47^ (Module #59, OR = 46, p = 0.003, Supplemental Fig. 17), and “microtubule anchoring at microtubule organizing center” in OPCs^48^ (Module #74, OR = 332, p = 3.8e-5, Supplemental Fig. 18). Therefore, we suggest that these networks capture aspects of cell type-specific pathways in each of the six major brain cell types.

**Figure 9:**
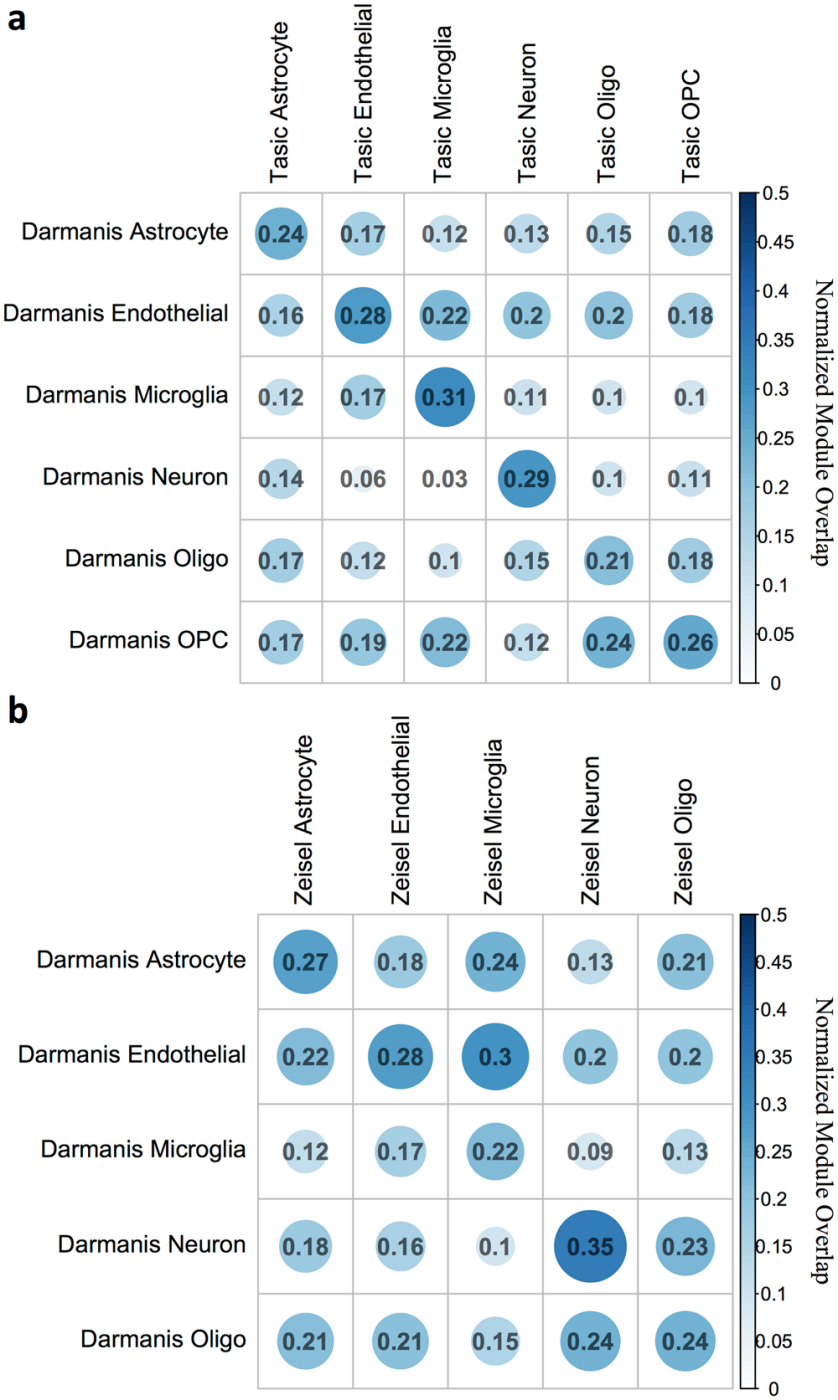
More significant module intersections are found within than between cell types across human to mouse data set comparisons. The proportion (between 0 and 0.5) of modules with significant intersections between MEGENA multiscale models is plotted for the human Darmanis *et al*. to mouse Tasic *et al*. (**a**) and human Darmanis *et al*. to mouse Zeisel *et al*. (**b**) comparisons. A module-module intersection between two cell type-associated multiscale networks was counted as a significant intersection (or overlap) if its Benjamini-Hochberg-adjusted Fisher’s Exact Test p-value was < 0.05. To generate proportions for the number of overlapping modules relative to each mouse cell type module set, the overlap matrix was normalized by column.

To query the robustness of the Darmanis *et al*. human networks, we measured the conservation of these networks in the cell type-specific networks found in each of the mouse Tasic *et al*. and Zeisel *et al*. data sets. We tested the hypothesis that there would be a higher proportion of modules from the same cell type that have significantly intersecting genes. Consistent with this hypothesis, we found that a higher proportion of significant modules were found in human-mouse pairings rather than the off-diagonal modules in both the Tasic *et al*. (Fig. 9A) and the Zeisel *et al*. (Fig. 9B), with the exception of oligodendrocyte-OPC pairings, which share many gene expression pathways, and the Darmanis *et al*. endothelial and Zeisel *et al*. microglia pairing. Importantly, the presence of some modules with significantly intersecting genes between cells of different types is also expected, because many pathways are shared across brain cell types. For example, Module #37, a 187-gene module in the Darmanis *et al*. multiscale neuron network significantly intersects with at least one other module in each of the human brain cell multiscale networks including astrocytes (Module #25, FE = 9.7, p = 2e-08), endothelial cells (Module #5, FE = 5.4, p = 2.1e-07), oligodendrocytes (Module #4, FE = 9.5, p = 4.5e-9), and OPCs (Module #4, FE = 4.3, p = 2.1e-9), but with the exception of microglia (Module #2, FE = 4.0, p = 0.1). Consistent with the presence of this module in most brain cell types, the neuron network Module #37 is not significantly enriched in neuron-specific genes, and is most enriched in genes associated with the GO term “respiratory chain complex” (OR = 10, p = 0.0049, Supplemental Fig. 13), which is a well-conserved pathway across cell types. The top hub gene in the neuron network Module #37 is *NCOA2*, which encodes the transcriptional activator *TIF2*, a gene that has been found to regulate mitochondrial respiration in skeletal muscle^49^. Taken together, these results show that the multiscale networks we identified in the scRNA-seq data are robust both across and within cell types.

## Discussion

In this study, we sought to compare and contrast five recently generated brain cell type RNA-seq data sets. As expected, we identified strong cell type-associated differential expression signatures within each of the data sets. We found that all three of the cell type-associated gene measures that we developed have substantial overlap between data sets, across both human and murine comparisons, and immunopanning cell population to single-cell comparisons. As expected, genes that were the most enriched in each brain cell type include well-known cell type marker genes. However, we also were able to identify potentially novel marker genes that are robustly enriched in particular cell types across data sets, such as *SLC39A12* in astrocytes and *VSTM2A* in neurons. We validated the utility of our collection of cell type-specific marker genes in a postmortem bulk gene expression data set, finding that individual marker genes may not be reliable in any given data set, while large sets of top marker genes have higher validity. We further screened for potential novel cell type markers by PubMed search and found that the gene symbols of more than 60% of the top specificity markers coincide with predicted cell types in at least one literature. Using an orthogonal dataset of ATAC-seq profiling of chromatin accessibility for different cell types in human brain tissue, we found that the top novel markers showed significantly higher levels of chromatin accessibility at their TSS in the matching ATAC-seq cell type than in non-matching cell types. We next performed multiscale network modeling of cell types in the scRNA-seq data, showing that this approach captured overlapping subcellular compartments and pathways within cells, and that the enrichment of the identified modules in our sets of consensus marker genes yields insight into their cell type-specificity and associated function. Overall, we expect that the tools we have developed for cell type associated analysis may be of interest for investigators analyzing both bulk and scRNA-seq data in the brain.

One important relationship between the three measures we defined is that absolute expression is strongly predictive of both cell type specificity and enrichment, as genes with higher absolute expression are often considered more trustworthy as marker genes. In order to incorporate this insight into our approach, we employed shrinkage estimation of the differences in expression between cell types when calculating cell type specificity and enrichment (see Methods). While the utilization of shrinkage estimation was fruitful in our study, the optimal amount of shrinkage remains unclear. Future studies should evaluate different approaches to the trade-off of choosing between marker genes that are highly specific/enriched but have lower overall expression and marker genes that are less specific/enriched but have higher overall expression. Notably, the optimal choice on this trade-off may differ for each study based on how much sample there is and/or how deep of sequencing a given experiment employs. By interrogating the overlap of the three cell type-associated measures, we were also able to identify genes that have both high absolute expression within a cell type and strong cell type-specific and cell type-enriched expression. These high expression cell type markers may be especially useful in experiments with limited sample quantity.

Because one of the validations of our cell type proportion estimation method is through the use of the Allen Brain Atlas (ABA) Aging, Dementia, and TBI study data set, it is worthwhile to consider some of the limitations of our approach. First, the ABA data set contains protein markers as measured via immunohistochemistry (IHC) as the independent measure of relative cell type proportion in each sample. An alternative data set might use fluorescence-activated cell sorting (FACS) to quantify relative cell proportions. While FACS would still rely on an individual marker gene, it would be more robust to differences in the expression of that marker gene within cells across samples. Second, the data set had IHC measurements corresponding to only two cell types, astrocytes and microglia, which limited our ability to query the cell type proportion estimation of the other four cell types that we generated signatures four. Finally, the ABA data set includes the frontal white matter as one of its brain regions, whereas the data sets that we used to generate the signatures were dissected from primarily grey matter brain regions. This may help explain why the rank correlations between the RNA-derived cell type proportion estimates and the IHC quantifications in the frontal white matter region were among the lowest. In part because of these limitations, we focused our major analysis of the ABA data set on an analysis that leverages the maximum statistical power across brain regions. In the future, alternative data sets that transcend some of the limitations of the ABA data set could be used to query the effectiveness of brain cell type proportion estimation methods from bulk RNA expression data.

One of the major ways that the consensus cell type-associated signatures that we identified in this study can be used is in cell type proportion estimation and deconvolution of bulk gene expression data. Cell type deconvolution analysis of bulk gene expression data from tissues made up of a mixture of cell types, such as the brain, is of considerable importance because of the large confounding effect of cell type composition differences in bulk gene expression samples^50^, and several approaches have been designed to address it^51,52^. We adapted a straightforward eigengene decomposition-based approach, which has been proposed and validated by CellCODE^53^, that leverages our consensus cell type-specific marker genes to estimate surrogate cell type proportions variables, which we implemented in a freely available R package, BRETIGEA. After estimation from bulk brain gene expression samples, the surrogate cell type proportion variables can be used to find the correlation of cell type changes with matched phenotype data, for example to uncover cell type proportion changes in disease states. The cell type proportions can also be used as a covariate for adjusting away the effect of brain cell type proportions on the expression of individual genes, which can be followed by downstream applications such as differential expression and/or differential correlation analysis. The entire process of estimating cell type proportions and deconvoluting them out of a bulk gene expression matrix has been conveniently implemented as a function in BRETIGEA. Generating scRNA-seq samples is an alternative way to address this problem, although the large degree of heterogeneity of cell types necessitates a several-fold increase in the number of necessary samples. Furthermore, there may be biases in the process of single cell selection and amplification, which means that bulk RNA-seq followed by cell type proportion deconvolution may still have advantages. Studies designed to directly compare the relative strengths of both of these strategies in disease or gene perturbation contexts are warranted.

As an important aspect of our study is the use of multiple cell type-specific marker genes to estimate surrogate cell type proportion variables, we offer some guidelines on how many marker genes should be selected. The major trade-off involved is quality versus quantity: the highest-ranked cell type-specific markers have the strongest effects and are among the most robust across data sets, while leveraging a larger number of marker genes averages away any stochasticity in the expression of individual genes and any systematic differences in their gene expression across groups, e.g. due to differences in gene expression between cases and controls. For example, in the Allen Brain Atlas RNA expression data, we found that individual marker genes and small sets of marker genes performed less well, while approximately 50 marker genes led to the best performance. In general, we recommend that investigators use approximately 50 marker genes, which is the default option in BRETIGEA, unless their data only measures expression from a subset of cell type marker genes, in which case a smaller number of genes such as 25 may be more appropriate. On the other hand, in performing the enrichment tests, we used 500 cell type-specific marker genes, because in this context we were more interested in the statistical power of the enrichment as opposed to precise numerical cell type proportion estimates, and enrichment tests tend be more highly powered upon leveraging a larger set of reference genes.

It is worthwhile to consider how the analysis and tools presented in this manuscript fit into the growing ecosystem of research involving scRNA-seq. One of the key steps that our approach is helpful for is in cell type identification of samples from single cells. While cell type identification in scRNA-seq data analysis is sometimes completely unsupervised, it is often an iterative process that involves filtering of the expression matrix for cell type markers and then performing clustering analysis on that filtered matrix^46^. In this case, sets of independent cell type markers such as the ones that we have developed in this manuscript are critical. A degree of supervised clustering analysis may be especially important when investigators are using scRNA-seq to compare single cell samples from healthy and diseased tissue. In this case, it can be difficult to distinguish whether a new cell type has emerged in a diseased state, or whether an existing cell type has alterations in the expression of a key set of genes. Large sets of marker genes are essential to leverage for this purpose, as any individual marker gene could have altered expression as the result of the disease process itself. Therefore, we suggest that as the field of molecular neuroscience moves increasingly to scRNA-seq-based analysis, the use of well-validated brain cell type marker gene sets such the ones that we present in this manuscript will be critical.

While we have focused in this manuscript on analyzing six major cell types in the brain, *i*.*e*. astrocytes, endothelial cells, microglia, neurons, oligodendrocytes, and OPCs, it is also important to consider the growing trend towards sub-cell type gene expression analysis in the brain. For example, recent studies have shown that there are more than thirty distinct subtypes of neurons in the hypothalamus^54^, as well as many distinct subtypes along the spectrum of differentiation between oligodendrocyte precursor cells and myelinating oligodendrocytes^55^. If investigators are interested in querying whether all of the sub-cell types fit the expected gene expression patterns of a given major cell type, then they may find the marker genes that we have annotated in this manuscript to be helpful. However, it is also possible that investigators will require a more fine-grained set of marker genes for these sub cell-types than the markers for the six major cell types we have analyzed here. In this case, then we expect that the methods of identifying cell type-specific and cell type-enriched genes in those sub-types, which we have developed and validated here across multiple data sets, may be of use to researchers involved in this area.

Our multiscale network modeling within each brain cell type reveals an additional modality in which cell type-specific marker genes can be of use to investigators. In particular, we found that analyzing the enrichment of our cell type-specific marker genes within each of the identified modules was a fruitful way to interrogate their functions. First, cell type enrichment analysis helped us to identify which modules were likely to be found within only one cell type, such as the neurotrophin-related module that we identified in the multiscale neuron network. Second, by identifying which modules were not enriched for cell type-specific genes, it helped us to identify which modules were likely to be present in many cell types, such as the pan-cellular respiratory chain complex-related module we identified, which is likely associated with mitochondria. Notably, the presence of a mitochondria-associated module makes biological sense, because mitochondria are present in all brain cell types, and the variability in their abundance within single cells will lead to a correlation in their RNA expression levels that can be captured by the multiscale network model. Third, it may help to identify modules that are not truly present within a cell type, but instead are called as such due to cell type misclassification or microfluidic cell double capture events. Notably, the presence of such modules may also be due to cell type heterogeneity, which makes their interpretation challenging.

To summarize the gene expression data that we generated, we present a web visualization tool made using R/Shiny, available at http://celltypes.org/brain/. This tool allows users to rapidly query the cell type expression patterns of the genes that they are interested in. Users can choose to log transform the data, view the expression in the main cell types or sub-cell types, view the exon-level data, and/or download the data to perform their own analyses. One way that investigators may find this particularly valuable is in investigating any differences between human and murine gene expression in brain cells that are relevant in comparative neurobiology. For example, *PMP2*, a gene which encodes Peripheral Myelin Protein 2, is highly expressed in astrocytes in both human data sets (ranked #104 and #172 in absolute expression in the two human data sets), whereas it has a low expression in all of the murine astrocyte expression signatures (ranked < #15,000 in absolute expression in astrocytes in all three of the mouse data sets). Because we analyzed all of the five data sets using an integrated pipeline, it is more likely that any such major differences that segregate by species are due to biological rather than technical reasons. In the future, as additional cell type-associated gene expression data sources become available from the brain and other tissues, we plan to add them to the website, so that users can further query the robustness of the cell type-associated gene expression patterns in which they are interested.

## Conclusions

We set out to compare and contrast several cell type-specific transcriptome-wide RNA expression data sets published within the last few years. Using a series of novel pipelines designed to facilitate meta-analysis, our results suggest that there is a large degree of conservation of cell type enrichment across data sets. This conservation is seen in comparisons between human and mouse data sets, and comparisons between populations of cell types and isolated single cells. Through the consensus marker genes that we identified, we designed a procedure for cell type proportion estimation from bulk RNA expression data, which we validated on gene expression from postmortem brain tissue. We identified potential novel cell type markers, which were subsequently validated by studying chromatin accessibility using ATAC-seq in different cell types isolated from human postmortem brain samples. We further generated multiscale networks from the single-cell RNA-seq data, which allowed us to explore and annotate fundamental brain cell pathways. We expect that our results will be useful to investigators studying the cellular and molecular operations of the brain across diverse fields of neuroscience.

## Methods

### Description of human brain cell gene expression data sets

Darmanis *et al*.^16^ used adult human anterior temporal lobe brain tissue that was resected while patients were undergoing epilepsy surgery and was considered normal via pathological examination. They made a single-cell suspension from this tissue via incubation in a solution containing papain, followed by washing and trituration. To reduce what they considered contamination from myelin debris and vascular macrophages, they performed immunopanning depletion by incubating the single-cell suspension in plates containing anti-CD45 antibodies followed by plates containing anti-GalC hybridoma antibodies. They then captured single cells and generated cDNA libraries from those single cells using the Fluidigm C1 system, followed by sequencing on the Illumina NextSeq platform. Based on RNA-seq gene expression, the authors performed clustering analysis to identify cell types. We downloaded cell type annotations and the raw sequencing read data from Gene Expression Omnibus (GEO; GSE67835). As described in the following method section, we reprocessed the RNA-seq data using a unified pipeline to quantify both gene- and exon-level expression for each sequencing library.

Zhang *et al*.^15^ collected normal temporal lobe samples from human brains resected from epilepsy patients in the same manner described in the Darmanis *et al*. study. They also incubated the tissue with papain in order to create a single-cell suspension. Next, the authors plated the suspended cells onto a series of immunopanning dishes coated with particular antibodies in order to isolate cell type populations of interest. The cell type populations that they isolated were microglia/macrophages (with an anti-CD45 antibody), oligodendrocytes (with an anti-GalC hybridoma antibody), neurons (with an anti-Thy1 antibody), astrocytes (with an anti-HepaCAM antibody), and endothelial cells (via binding reactivity to BSL-1). Although the authors did not distinguish between myeloid cell types (i.e., between microglia and macrophages), we considered these to be microglia for the purpose of cross-data set comparison. The cells were scraped off the immunopanning dishes and treated with the Qiagen miRNeasy kit to extract total RNA. The authors then generated cDNA libraries and sequenced them with the Illumina NextSeq platform. They mapped the reads to human genome version 19 (hg19) using TopHat2, and used Cufflinks to estimate expression levels for each gene as FPKM. We downloaded the sequencing read data from GEO (GSE73721) and used the samples they annotated as mature astrocytes from normal tissue samples as the astrocyte cell type population, discarding the samples annotated as astrocytes from diseased tissue samples.

### Description of mouse brain cell gene expression data sets

Zhang *et al*.^17^ dissected brain tissue from mice and dissociated single cells, followed by fluorescence activated cell sorting (FACS) or immunopanning to isolate populations of brain cell types. They collected cells from pooled litters of postnatal day 7 (P7) FVN/Swiss mice. The sex of the mice in these litters was not specified. The cell types included astrocytes (using a transgenic mouse expressing EGFP driven by regulatory sequences in Aldh1l1–Bacterial Artificial Chromosome), neurons (using an anti-L1CAM antibody), microglia (using an anti-CD45 antibody following PBS washing of blood), oligodendrocyte precursor cells (using an anti-PDGFRα antibody), newly formed oligodendrocytes (using an anti-GalC antibody), myelinating oligodendrocytes (using an anti-MOG antibody), endothelial cells (using EGFP driven by the pan-endothelial Tie2 promotor), and pericytes (using an anti-PDGFRβ antibody). From these cell populations, they isolated total RNA using the Qiagen miRNeasy kit, generated cDNA libraries from the total RNA, and sequenced the cDNA libraries using the Illumina HiSeq. 2000. We downloaded the sequencing read data from GEO (GSE52564).

Zeisel *et al*.^18^ extracted tissue from the somatosensory cortex and hippocampus of CD-1 mice (P21-P31). They isolated cells from P22-P32 CD1 mice that were a mix of 33 males and 34 females. They dissociated single cells via incubation in papain protease followed by filtration through a 20 µm filter. A subset of the cells was dissected from 5HT3-EGFP mice, which were subsequently FACS sorted to enrich for interneurons. The authors used the Fluidigm C1 system to isolate single cells, followed by cDNA library construction and sequencing using the Illumina HiSeq. 2000. We used the clustering-derived cell type annotations that they computed as a starting point to group our more general cell types of interest, and pooled the cell types across the two brain regions they analyzed. Specifically, we grouped each of their multiple astrocyte, endothelial, and microglia cell type classifications into one group for each cell type, while we classified their Oligo5 and Oligo6 into a mature oligodendrocyte cell type population. Further, we pooled all of the interneuron and pyramidal cell types that they identified into one neuron cell type population. None of the other oligodendrocyte sub-classes that they identified were found to be similar enough to OPCs to include that as a cell type in our analysis. We downloaded the sequencing read data from GEO (GSE60361).

Tasic *et al*.^19^ sectioned brains and microdissected the primary visual cortex (V1) of 8-week old mice that expressed the fluorescent protein tdTomato (tdT) in a particular cortical cell type, through the use of a Cre recombinase system. They isolated cells from 8-week old male C57BL/6 J mice. From the dissected brain tissue, the authors generated single-cell suspensions via trituration through μm-scale Pasteur pipettes, followed by FACS to isolate single cells with low DAPI, and, in the majority of cases, high tdT. They used the Illumina SMARTer Ultra Low RNA Kit to create a cDNA library, and the Illumina HiSeq. 2000 for sequencing. The authors also performed and validated clustering to identify cell types, which we used as annotations for our analyses. Notably, we pooled all the neuron sub-classes from all cortical layers that the authors identified into a single neuron cell type. We downloaded the sequencing read data from GEO (GSE71585).

### Read mapping and data pre-processing

For each human or mouse RNA-seq datasets analyzed, we downloaded published raw sequencing data from GEO (Table 1). While either normalized gene expression measures or gene-level read counts data were provided along with the original publications, the RNA-seq data from the same species were processed by different pipelines in different studies, thus resulting in different gene expression quantification metrics, rendering complexity and incompatibility in conducting a cross-dataset comparison. To simplify the downstream data analysis, we reprocessed the sequencing alignment and gene expression normalization using a unified and efficient pipeline built upon the STAR aligner^56^, featureCounts^57^, and R/Bioconductor package edgeR^58^. The details about these processes are included in the Supplemental Methods.

### Cell type-associated gene expression measurements

We consider three types of genewise cell type-relative expression measurements: specificity, enrichment, and absolute expression levels (Fig. 1). Specificity is defined as the difference between a gene’s expression in the cell type of interest compared to the other cell type in which it has its highest expression. Enrichment is defined as the difference between a gene’s expression in the cell type of interest compared to all of the other cell types. Finally, absolute expression is defined as the relative expression of a gene within a cell type, irrespective of that gene’s expression in other cell types. To calculate specificity and enrichment, we first filtered the data sets to retain only those genes that had an average (arithmetic mean) of at least five read counts in at least one cell type. Next, we estimated the dispersion of each gene and fit a negative binomial generalized linear model to the count data using the R package edgeR^58^. In all data sets, cell type was modeled as a covariate, alongside adjustment covariates specific to each data set (Table 1). We set the prior.count variable in edgeR to 10, which adds pseudocounts to each observation relative to the library size of each sample, thus increasing the proportion of shrinkage to allow for more robust signature estimation. To calculate cell type enrichment, we compared the expression of samples annotated to that cell type, which we call the cell type of interest, to the expression of samples annotated to all the other major brain cell types, which we call the reference cell set. For example, samples annotated to astrocytes were compared to samples annotated to endothelial cells, neurons, microglia, oligodendrocytes, and OPCs. The exception is that either oligodendrocytes or OPCs were excluded from the reference cell set when the other was the cell type of interest, since their expression patterns are too similar to allow for meaningful reference comparisons. To calculate cell type specificity, we performed contrasts on the fitted models that compared each cell type to all reference cell types individually, and chose the minimum resulting fold-change for each cell type of interest. For each of the cell types in each of the data sets, we created a volcano plot with the results of the differential expression enrichment analysis and highlighted the genes that had Benjamini-Hochberg^59^ adjusted p-value less than 0.05 and fold-change in the cell type of interest versus the others of greater than or equal to 4.

To calculate absolute expression within each cell type, we first normalized count values by the quantile method, as above, as well as by read length in order to generate RPKM (Reads Per Kilobase of transcript per Million mapped reads) values. We then calculated the arithmetic mean of the RPKMs within each cell type, and quantified the associated dispersion by finding the standard error of the mean. Genes within each sample were ranked by their expression values in order to facilitate cross data set comparisons.

### Comparison of cell type-enrichment gene rankings across data sets

We first found the fold enrichment of the intersection of the top *n* genes (where n = 10, 20, 50, 100, 200, 500, and 1000) ranked according to one of the three cell type expression-based measures in each of the data sets and cell types, after pairwise merging of the data sets to include only gene symbols that were common to both. To calculate the fold enrichment (FE) we used the equation:

1 $${FE}=\frac{|{D}_{1n}{\cap }^{}{D}_{2n}|}{\frac{|{D}_{1n}|\times |{D}_{2n}|}{|U|}}$$

where *D*_*1n*_ is the set of genes in the first data set for a given number of top genes *n*, *D*_*2n*_ the corresponding set of genes in the second data set, *U* is in the universe of gene symbols from the intersection of the data sets, and the cardinality operators indicate the size of the gene sets. Next, we calculated the percentage of genes that overlapped in both pairwise and global data sets comparisons of the top *n* gene signatures in each cell type. Finally, we merged the five data sets to include only gene symbols that were common to all, and used the SuperExactTest R package (version 0.99.2)^60^ to find the fold enrichments and p-values of the intersection of global intersection analyses.

### Ranking of genes by cell type-enrichment across data sets

To compare the cell type-specificity and -enrichment of genes across data sets, we calculated the median of the corresponding log fold changes across the data sets that contained that cell type. Because not all gene symbols were present in all of the data sets, we required that the gene symbol be present in more than half of the data sets in order to be included in the overall ranking list. Further, for the combined mouse-human comparison, we required that the gene symbol be present in at least one of each of the human and mouse data sets. For each cell type, genes were then ranked within each cell type by their median fold-change across the data sets. To compare the cell type-expression measurements of genes across data sets, we first converted all of the gene expression values within each data set to ranks according to expression within each cell type. For each cell type, genes were then ranked within each cell type by their median fold-change or their median expression across the data sets. Gene rankings were then aggregated across the data sets for each cell type by calculating the grand median ranking.

### Estimation of cell type proportion from bulk gene expression data

We next used matched RNA expression and immunohistochemistry marker data from the Aging, Dementia, and TBI study from the Allen Brain Atlas^39^ as an additional validation of the marker genes identified in bulk brain gene expression data from the Allen Brain Atlas Aging, Dementia, and TBI study. To make these estimates, we adapted the previously validated singular value decomposition (SVD) method from CellCODE^53^, which has been implemented in the BRETIGEA (BRain cEll Type specIfic Gene Expression Analysis) R package (version 1.0). The details of the estimation are included in the Supplementary Materials.

### Multiscale network analysis in the single cell RNA expression data sets

MEGENA networks were constructed from genes expressed in at least half of the samples for each cell type in each data source by using the R package MEGENA (version 1.3.4-1). Briefly, Pearson correlation coefficients (PCCs) were firstly computed for all gene pairs. The gene pairs with absolute PCCs larger than 0.3 were ranked and iteratively tested for planarity to grow a Planar Filtered Network (PFN). Multiscale clustering analysis was conducted with the resulting PFN to identify coexpression modules at different network scale topology under the default parameter setting of the package.

## Electronic supplementary material

Supplementary Methods and Results

Supplemental File 1

Supplemental File 2

Supplemental File 3

Supplemental File 4
