# Author Correction: Brain Cell Type Specific Gene Expression and Co-expression Network Architectures

**DOI:** 10.1038/s41598-021-97284-6

**Published:** 2021-09-24

**Authors:** Andrew T. McKenzie, Minghui Wang, Mads E. Hauberg, John F. Fullard, Alexey Kozlenkov, Alexandra Keenan, Yasmin L. Hurd, Stella Dracheva, Patrizia Casaccia, Panos Roussos, Bin Zhang

**Affiliations:** 1 Department of Genetics and Genomic Sciences, Icahn School of Medicine at Mount Sinai, New York, NY 10029 USA; 2 Icahn Institute of Genomics and Multiscale Biology, Icahn School of Medicine at Mount Sinai, New York, NY 10029 USA; 3 Medical Scientist Training Program, Icahn School of Medicine at Mount Sinai, New York, NY 10029 USA; 4 Friedman Brain Institute, Icahn School of Medicine at Mount Sinai, New York, NY 10029 USA; 5 iPSYCH, The Lundbeck Foundation Initiative for Integrative Psychiatric Research, 8000 Aarhus, Denmark; 6 Department of Biomedicine, Aarhus University, 8000 Aarhus, Denmark; 7 Department of Psychiatry, Icahn School of Medicine at Mount Sinai, New York, NY USA; 8 Mental Illness Research, Education, and Clinical Center (VISN 2), James J. Peters VA Medical Center, Bronx, NY USA; 9 Fishberg Department of Neuroscience, Icahn School of Medicine at Mount Sinai, New York, NY 10029 USA; 10 Neuroscience Initiative, Advanced Science Research Center, The City University of New York, 85 St. Nicholas Terrace, New York, NY 10031 USA

Correction to: Scientific Reports 10.1038/s41598-018-27293-5, published online 11 June 2018

The original version of this Article contained an error in Figures 4 and 5 where the Figure bodies were swapped.

**Figure 4:**
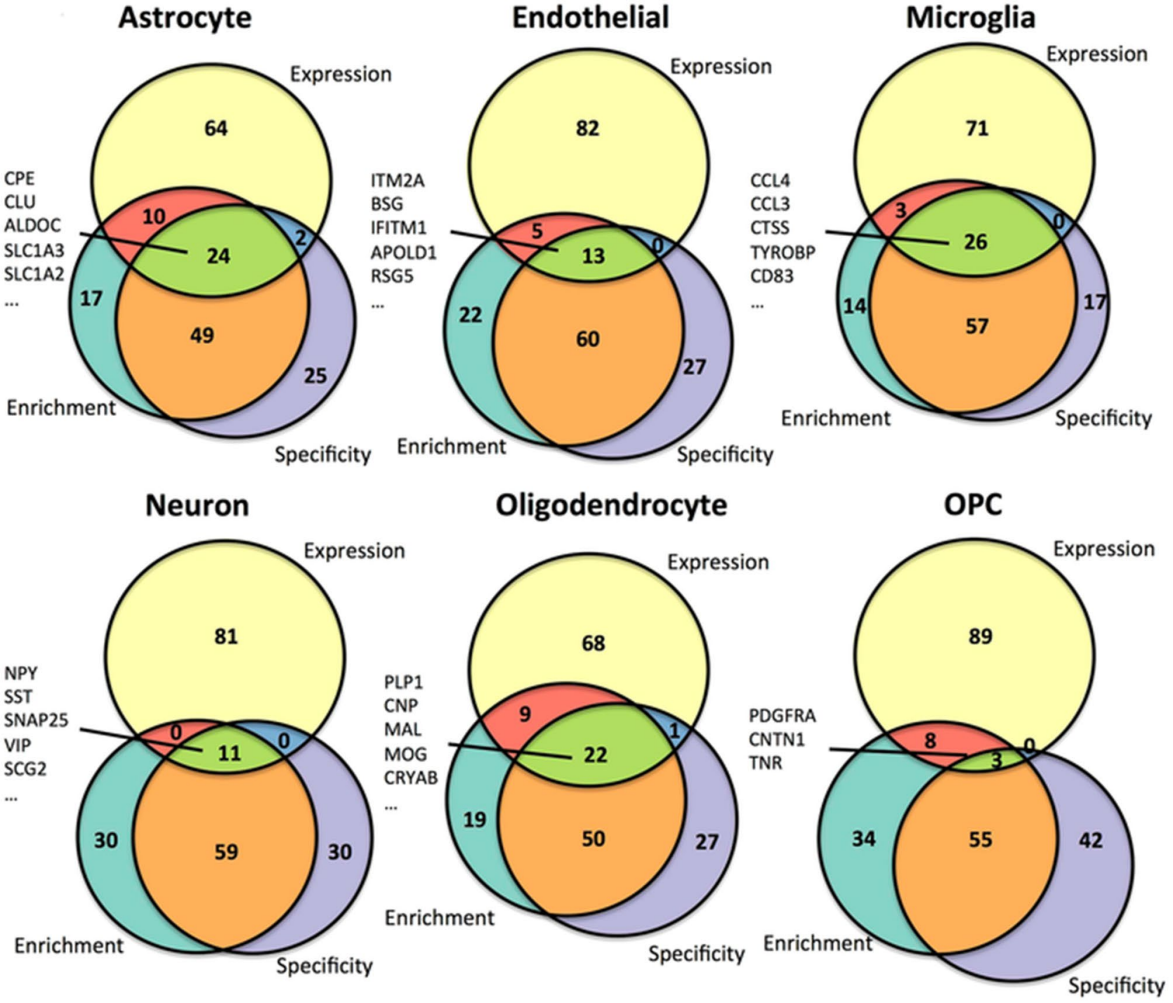
Correlation of genewise cell type enrichment measures with PubMed text mining results within cell types. For each of the six cell types, i.e. the astrocyte (**a**), endothelial cell (**b**), microglia (**c**), neuron (**d**), oligodendrocyte (**e**), and oligodendrocyte precursor cell (**f**), the top 100 gene symbols most enriched in that cell type are plotted against the number of PubMed abstracts that mention both that gene symbol as well as the corresponding cell type. The Spearman correlation between these measures was calculated. Several gene symbols were chosen for highlighting, including gene symbols that have not been mentioned in a PubMed abstract with that cell type to date (labeled red). Note that for oligodendrocyte precursor cells (OPCs), the cell type name used in the PubMed search was “oligodendrocyte precursor.”

**Figure 5:**
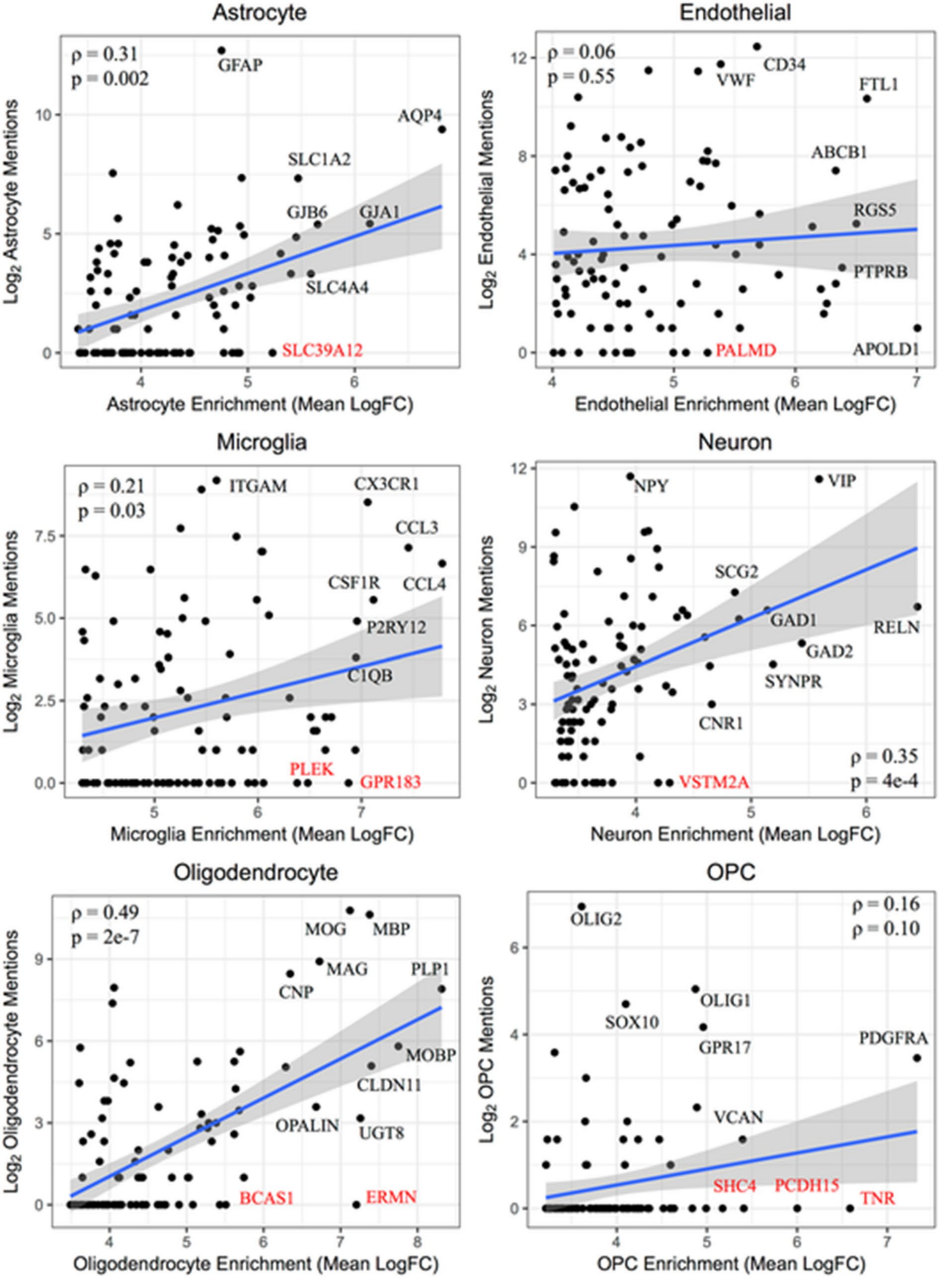
Intersections among the top genes for three cell type associated measures consensus rankings across all cell types. The top 100 genes ranked across both mouse and human data sets for each of the cell type measures are intersected using approximately proportional Venn diagrams for each of the astrocyte (**a**), endothelial cell (**b**), microglia (**c**), neuron (**d**), oligodendrocyte (**e**), and oligodendrocyte precursor cell (**f**) signatures. The Venn diagrams were generated using the R package Vennerable (version 3.0). The 5 genes with the top expression values in each of the cell types that intersect in all three of the top 100 gene sets are listed, with the exception of OPCs, for which all 7 of the genes with intersections between the three measures are listed.

The original Figures 4 and 5 and accompanying legends appear below.

The original Article has been corrected.

