# Supplementary material for: Brain Cell Type Specific Gene Expression and Co-expression Network Architectures
Source: Sci Rep. 2018 Jun 11;8:8868. doi: 10.1038/s41598-018-27293-5 (PMC5995803; doi:10.1038/s41598-018-27293-5)
Supplement: Supplementary file 1 — Supplementary Methods and Results [file 41598_2018_27293_MOESM1_ESM.pdf]

Brain Cell Type Specific Gene Expression and Co-expression Network Architectures

Andrew T. McKenzie, Minghui Wang, Mads E. Hauberg, John F. Fullard, Alexey Kozlenkov, Alexandra Keenan, Yasmin L. Hurd, Stella Dracheva, Patrizia Casaccia, Panos Roussos, and Bin Zhang

Supplementary Methods

Read mapping and data pre-processing

For each human or mouse RNA-seq datasets analyzed, we downloaded published raw sequencing data from GEO (**Table 1**). While either normalized gene expression measures or gene-level read counts data were provided along with the original publications, the RNA-seq data from the same species were processed by different pipelines in different studies, thus resulting in different gene expression quantification metrics, rendering complexity and incompatibility in conducting a cross-dataset comparison. To simplify the downstream data analysis, we reprocessed the sequencing alignment and gene expression normalization using a unified and efficient pipeline built upon the STAR aligner[1], featureCounts[2], and R/Bioconductor package edgeR[3]. With this pipeline, the raw sequencing reads were aligned to either human hg38 genome or mouse mm10 genome using the STAR aligner. For human datasets, GENCODE gene annotation model GRCh38.p2 release 22 was used to assist with the alignment of reads to known gene features. For mouse datasets, the UCSC mm10 gene annotation model was used. Following read alignment, featureCounts was used to quantify the gene expression at both the gene and exon level. For the single cell data sets, the scater R package (version 1.0.4)[4] was used to calculate the total number of features identified in each sample, which was used as a covariate in the differential expression models, as it may reflect biases in the initial amount of RNA prior to amplification. For the Darmanis *et al.* data set, 3 outlier samples were detected and removed on the basis of principal component analysis using the scater R package (SRR1974559, SRR1974625, and SRR1974648). The gene level read counts data was normalized using the quantile approach[3,5], with a percentile of 0.75 for the cell population data sets and 0.99 for the single cell data sets, to adjust for sequencing library size difference.

Gene symbol conversion

For all three murine data sets, the gene symbols were converted from MGI symbols to HGNC symbols by using the Ensembl database, accessed through biomaRt (version 2.28)[6]. If no homolog existed, then the original mouse symbol was retained. In the case of multiple homologous genes, we selected the human gene with the highest homology percentage based on protein coding region DNA divergence. In the case that multiple transcripts mapped to the same gene symbol, the gene symbol with the maximum average expression count was retained for subsequent analyses[7].

Comparison of cell type signatures to PubMed text mining results

For the top 100 most enriched genes in each cell type, we performed PubMed (https://www.ncbi.nlm.nih.gov/pubmed) searches and counted the number of results for the search "Gene AND CellType" (**Supplementary File 2**), where Gene refers to the character string of the enriched gene symbol and CellType refers to the character string of the enriched cell type, either "astrocyte", "endothelial", "microglia", "neuron", "oligodendrocyte", or "oligodendrocyte precursor". We used the Mann-Whitney U test to compare the number of PubMed mentions found among the corresponding most enriched gene set, compared to the genes most enriched in all of the other cell types, with the exception of oligodendrocytes and OPCs, which were not included in each other's control sets for comparison because of their similarity. We also found the Spearman correlation between the enrichment for each gene in that cell type and the number of PubMed mentions for the gene symbol/cell type combination for each cell type.

Comparison of the three cell-type associated gene expression measurements

We first selected genes such that they needed to be within the top 1000 genes as ranked by one of the measures at a time. We used this filtering method because, on one hand, we needed to remove low-ranked genes because the measures were not designed to be sensitive to differences in gene ranks at the lower ranks, because the expression of a gene in a given cell type for all data sets may be negligible. On the other hand, if we were to filter genes such that they ranked highly in at least one of the categories, we would have introduced a spurious association between the two variables, as a result of Berkson's bias. Therefore, we calculated the Spearman

(rank) correlation between gene rankings for each of the three measures after filtering for the top ranked genes in one measure at a time, for a total of unique six comparisons, for each of the six cell types.

Estimation of relative cell type proportions from bulk RNA expression data

To make relative cell type proportion estimates, we adapted the previously validated singular value decomposition (SVD) method from CellCODE[8], which has been implemented in the BRETIGEA (BRain cEll Type specIfic Gene Expression Analysis) R package (version 1.0). Specifically, after scaling the data, the algorithm in BRETIGEA calculates the first singular vector of a variable number of cell type-specific (marker) genes as an estimate of the relative cell type proportion in those samples. Because the signs of this singular vector can be reversed, we next find the mean correlation between the singular vector and each of the marker genes, and if this is less than zero, then the sign of all of the values in the singular vector are switched. Note that these estimated values can be less than zero for a given sample, which is part of why it is a relative cell type proportion estimate, rather than an absolute cell type proportion estimate. BRETIGEA also offers principal component analysis (PCA) as an option for cell type relative proportion estimation, along with sets of 1000 top consensus cell-specific genes for each cell type.

We used matched RNA expression and immunohistochemistry marker data from the Aging, Dementia, and TBI study from the Allen Brain Atlas[9] as an additional validation of the marker genes identified in bulk brain gene expression data. In this study, investigators performed both RNA-seq and immunohistochemistry (IHC) quantification for protein levels of the astrocyte marker gene GFAP and the microglia marker gene IBA1 in brain samples from the same donor across four brain regions (frontal white matter, hippocampus, temporal cortex, and parietal cortex). We downloaded the normalized RPKM values from the study website (http://aging.brain-map.org/download/index), which has been adjusted for RNA integrity number (RIN) and batch. The IHC quantifications provide an independent measurement for the relative levels of the cell types in each brain sample, which can be used to validate our relative cell type proportion estimates from the RNA expression data. Using all four brain regions available in the Allen Brain Atlas data set, we first calculated 100 SVD-based estimates of relative cell type proportions for astrocytes and microglia in each sample using the top

1-100 marker genes. We correlated all of these estimates with the independent IHC quantifications for GFAP and IBA in those samples. We also found the rank correlation between each of the individual top 100 marker genes for astrocytes and microglia with the independent IHC quantifications for GFAP and IBA1, respectively. As an additional exploratory analysis, we estimated the relative proportion of astrocytes and microglia using the SVD-based method *within* each of the 4 individual brain regions and calculated the rank correlations of these estimates with the IHC quantifications for GFAP and IBA1, respectively, in each of those brain regions.

ATAC-seq validation of novel cell markers

To validate our novel cell marker genes, we performed ATAC-seq on 50mg of frozen postmortem brain tissue from the dorsolateral prefrontal cortex from four controls (i.e. individuals that had no history of neuropsychiatric disease or substance abuse). Human brains from normal control Caucasian subjects without head trauma were collected at autopsy within 24 h after death at the Department of Forensic Medicine, Semmelweis University and at the National Institute of Forensic Medicine (Karolinska Institutet). All material was obtained under approved local ethical guidelines. Nuclei were processed for fluorescence activated nuclear sorting (FANS) as described previously[10]. ATAC-seq was performed on FANS sorted nuclei to assess chromatin accessibility following an established protocol[11]. We mapped ATAC-seq reads with STAR 2.50 and the following settings: --alignIntronMax 1 --outFilterMismatchNmax 100 --alignEndsType EndToEnd --outFilterScoreMinOverLread 0.3 --outFilterMatchNminOverLread 0.3. To rule out sample mislabelling and contamination, genotypes were called with GATK 3.50 and the genetic concordance between all combinations of samples calculated. Peaks were subsequently called with the MACS 2.1 narrow peaks algorithm with the following settings --keep-dup all --shift -100 --extsize 200 --nomodel. We created a consensus set of peaks by keeping only those that were found in two or more samples. Read counts were then quantified within these peaks using RSubread 1.15 and the following settings: allowMultiOverlap=F, isPairedEnd=T, strandSpecific=0, requireBothEndsMapped=F, minFragLength=0, maxFragLength=2000, checkFragLength=T, countMultiMappingReads=F, countChimericFragments=F. This yielded a peak by sample expression matrix, from which peaks mapping to the ENCODE blacklisted regions were excluded. Peaks that had very few read

counts (defined as peaks that had less than 1 count per million in 90% or more of the samples) were then discarded. For the remaining peaks, we normalized the read counts using "TMM" normalization followed by conversion of read counts to counts per million. We then constructed a peak by gene mapping by considering a peak mapped to a gene if a TSS overlapped the peak. Most often a gene overlapped, at most, one peak, but when it overlapped multiple peaks due to multiple transcripts we took the sum of the reads within the peaks. Additionally, genes not having a TSS overlapping an ATAC-seq peak were left out of further analyses. This yielded a gene by sample matrix of read counts. Subsequent averaging across samples from the same cell type yielded a gene by cell type matrix of read counts. This matrix was finally normalized by the sum of read counts for the given gene, resulting in a matrix for each gene indicating how large a fraction of the ATAC-seq read counts originated from a given cell.

Multiscale network analysis in the single cell RNA expression data sets

MEGENA networks were constructed from genes expressed in at least half of the samples for each cell type in each data source by using the R package MEGENA (version 1.3.4-1). Briefly, Pearson correlation coefficients (PCCs) were first computed for all gene pairs. The gene pairs with absolute PCCs larger than 0.3 were ranked and iteratively tested for planarity to grow a Planar Filtered Network (PFN). Multiscale clustering analysis was conducted with the resulting PFN to identify coexpression modules at different network scale topologies using the default parameter settings of the package. We visualized the networks derived from the MEGENA analysis using the prefuse force directed layout option in Cytoscape (version 3.2.1).

To visualize the gene ontology (GO) enrichment of distinct modules in the multiscale network, we filtered for modules containing between 5 and 2000 gene members, and selected only those modules with at least one gene that was specific to that module, thus removing exclusively parent modules. We used the moduleGO function in DGCA[12] (version 1.0.1) to perform gene ontology (GO) enrichment analysis on gene modules, which leverages the GOstats (version 2.34)[13] and org.Hs.eg.db GO annotation (version 3.1.2) R packages. To identify GO terms with potential brain cell type-specific activity, we filtered for those GO terms with less than 500 gene symbols. We adjusted the enrichment p-values for all GO terms in each module using

the Benjamini-Hochberg (BH) method. We used Fisher's Exact Test (FET) to identify cell type enrichments for each module, using the top 500 genes ranked by consensus cell type specificity from each cell type in the human data sets as the cell type enrichment signatures, and adjusted the module enrichment p-values for each cell type signature using the BH method. The background universe size used in the FETs was the number of unique gene symbols found in both the human and mouse data sets. To identify module conservation, we found the FET enrichment of the intersection of all the modules identified in each cell type from the Darmanis *et al.* human data set with all the modules identified in each cell type from the Tasic *et al.* and Zeisel *et al.* mouse data sets. We counted the number of significantly overlapping (BH-adjusted $p < 0.05$) modules in each of these comparisons. We corrected for the total number of overlapping modules identified within each mouse cell comparison set by normalizing the resulting overlap matrix by column sums.

Supplementary Data

Supplemental File 1. Cell type associated gene rankings across data sets.
The Excel file contains tab-separated tables with the rankings for all three cell type associated measures, i.e. cell type-enrichment, cell type-specificity, and cell type-expression, across all data sets, human-only, and mouse-only, in all 6 cell types.

Supplemental File 2. PubMed text mining results.
The Excel file contains tab-separated tables with the top 100 most enriched genes in each of the six cell types, as well as the number of PubMed abstracts for a search of each gene symbol and the cell type in combination, as well as the gene symbol alone.

Supplemental File 3. MGI phenotypes enriched in the cell type specific marker genes.
The Excel file contains the statistics from the enrichment test of MGI phenotype gene signatures in the cell type specific marker gene signatures.

Supplemental File 4. MEGENA modules and networks identified in the human and mouse single cell RNA-seq data sets.
The Excel file contains tab-separated tables with networks and modules identified by MEGENA within each major brain cell type (astrocyte, endothelial cell, microglia, neuron, oligodendrocyte, and OPC) available in each of the Darmanis *et al.*, Tasic *et al.*, and Zeisel *et al.* data sets.

Supplementary Figures

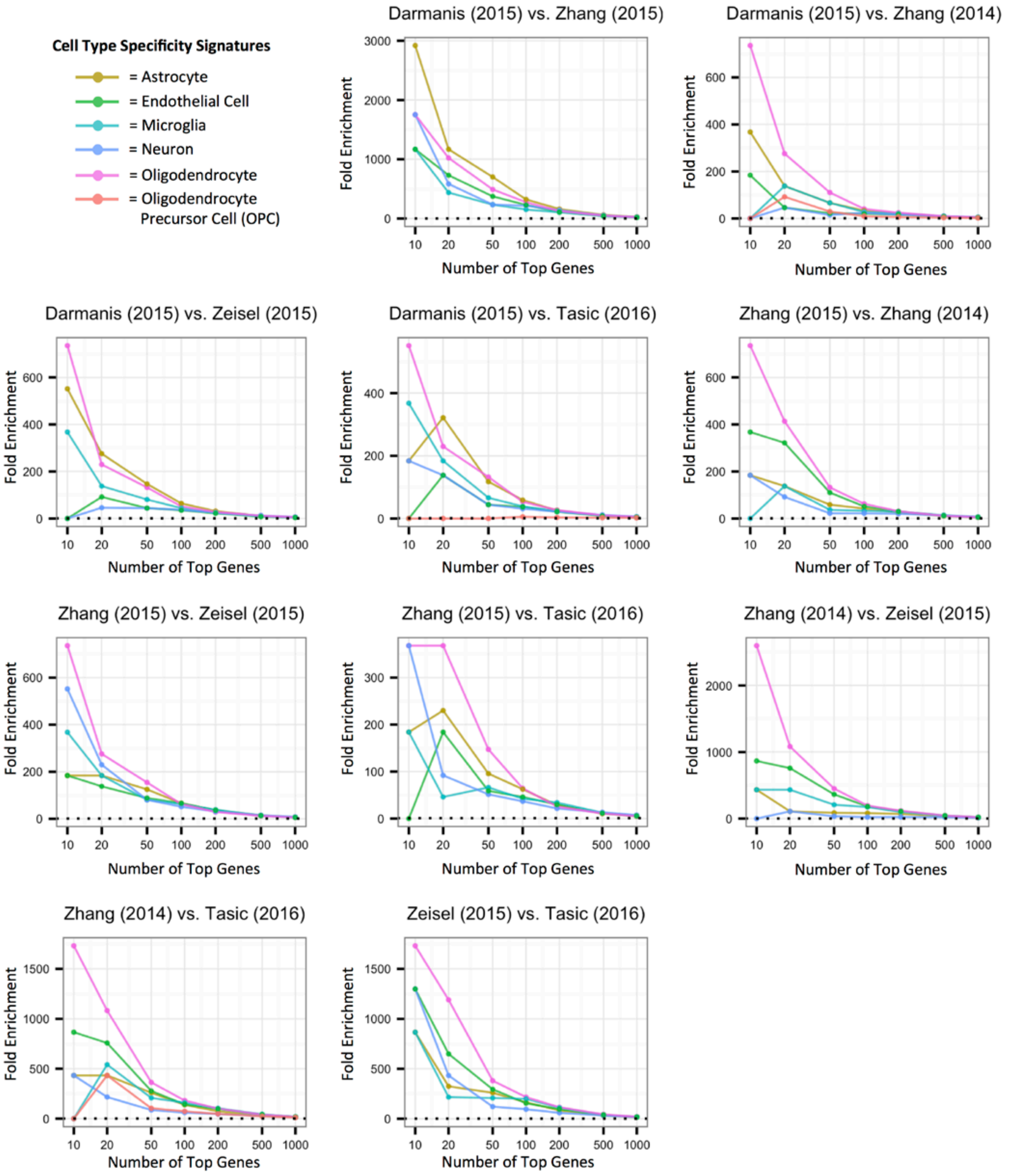

Supplementary Figure 1. Pairwise data set comparison of cell type specificity rankings for each cell type. Plots of the fold-enrichment of the intersections of genes ranked in the top n genes (where n = 10, 20, 50, 100, 200, 500, 1000) between pairs of data sets for the cell type specificity measure. The data sets were merged to only include gene symbols common to both prior to calculating the fold enrichment score. A fold enrichment of 0 indicates that no genes were found in the intersection of those two sets of top genes.

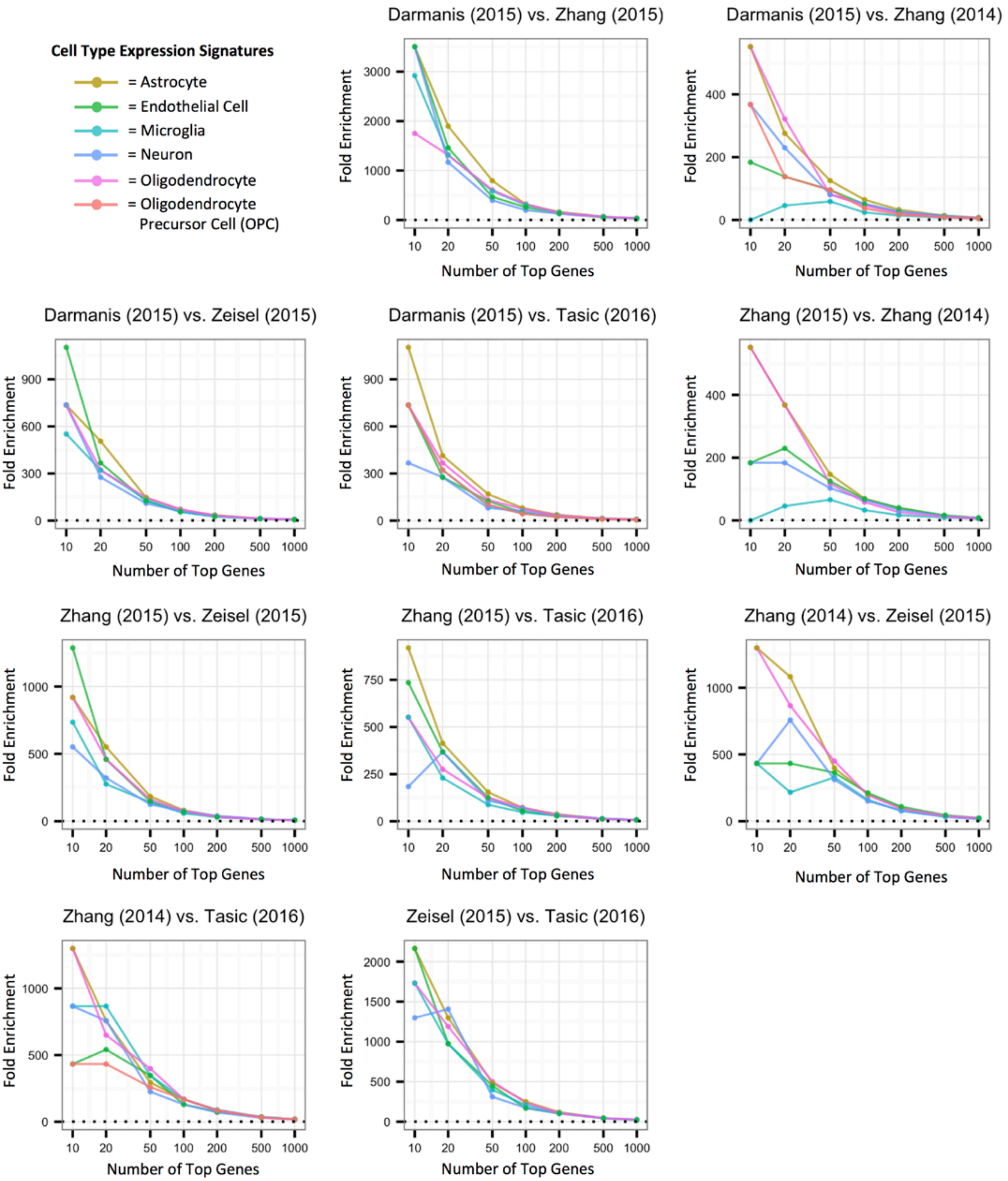

Supplementary Figure 2. Pairwise data set comparison of cell type absolute expression rankings for each cell type. Plots of the fold-enrichment of the intersections of genes ranked in the top n genes (where n = 10, 20, 50, 100, 200, 500, 1000) between pairs of data sets for the cell type absolute expression measure. The data sets were merged to only include gene symbols common to both prior to calculating the fold enrichment score. A fold enrichment of 0 indicates that no genes were found in the intersection of those two sets of top genes.

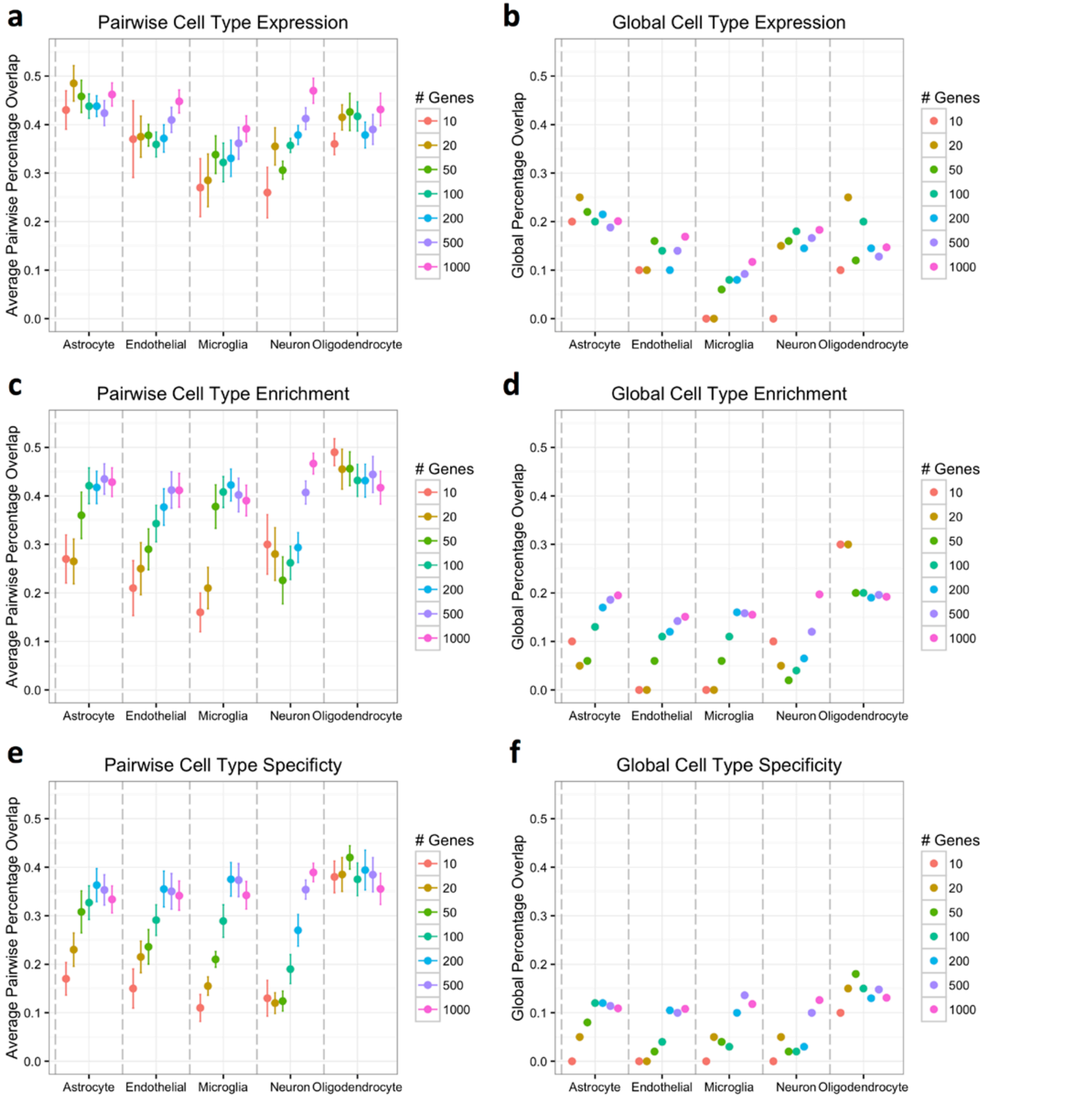

Supplementary Figure 3. Average percentage pairwise and global data set overlaps of top cell type-associated genes. **A, C, E**: The average percentage overlap (i.e., number of overlaps/number of top genes in each signature) of gene symbols between data sets for the top *n* genes in each cell type (at various values of n, i.e. n = 10, 20, 50, 100, 200, 500, 1000) ranked by each of the three cell type associated measures, i.e. cell type absolute expression (**A**), cell type enrichment (**C**), and cell type specificity (**E**). Dots = mean, bars = standard error of the mean. **B, D, F**: The percentage overlap of gene symbols between all five data sets for the top *n* genes in each cell type ranked by each of the three cell type associated measures, i.e. cell type absolute expression (**B**), cell type enrichment (**D**), and cell type specificity (**F**). Summary measures for oligodendrocyte precursor cells (OPCs) are not shown because this cell type is only present in 3 of the data sets, whereas the other cell types are represented in all 5 of the data sets.

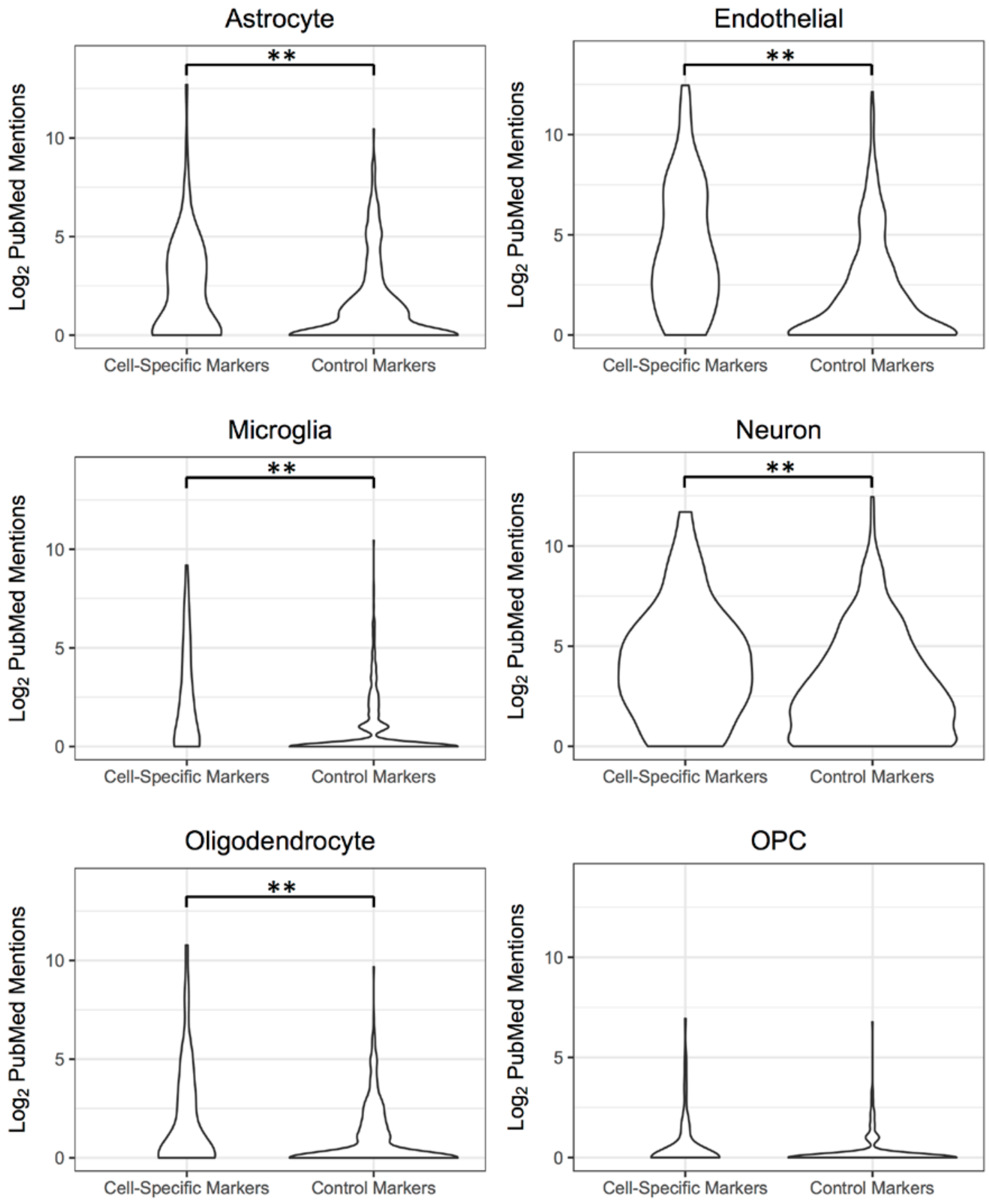

Supplementary Figure 4. PubMed text mining results of marker genes for a given cell type compared to other marker genes. For each of the six cell types, i.e. astrocyte (**A**), endothelial cell (**B**), microglia (**C**), neuron (**D**), oligodendrocyte (**E**), and oligodendrocyte precursor cell (**F**), a violin plot showing the number of PubMed abstracts that mention both that gene symbol as well as the corresponding cell type for the top 100 most enriched gene symbols in that cell type as well as the 500 other marker genes corresponding to each of the other cell types. A Mann-Whitney U test was used to compare the number of PubMed mentions for cell-specific markers compared to the number of PubMed mentions for the control markers ((*, $p < 0.05$; **, $p < 0.01$).

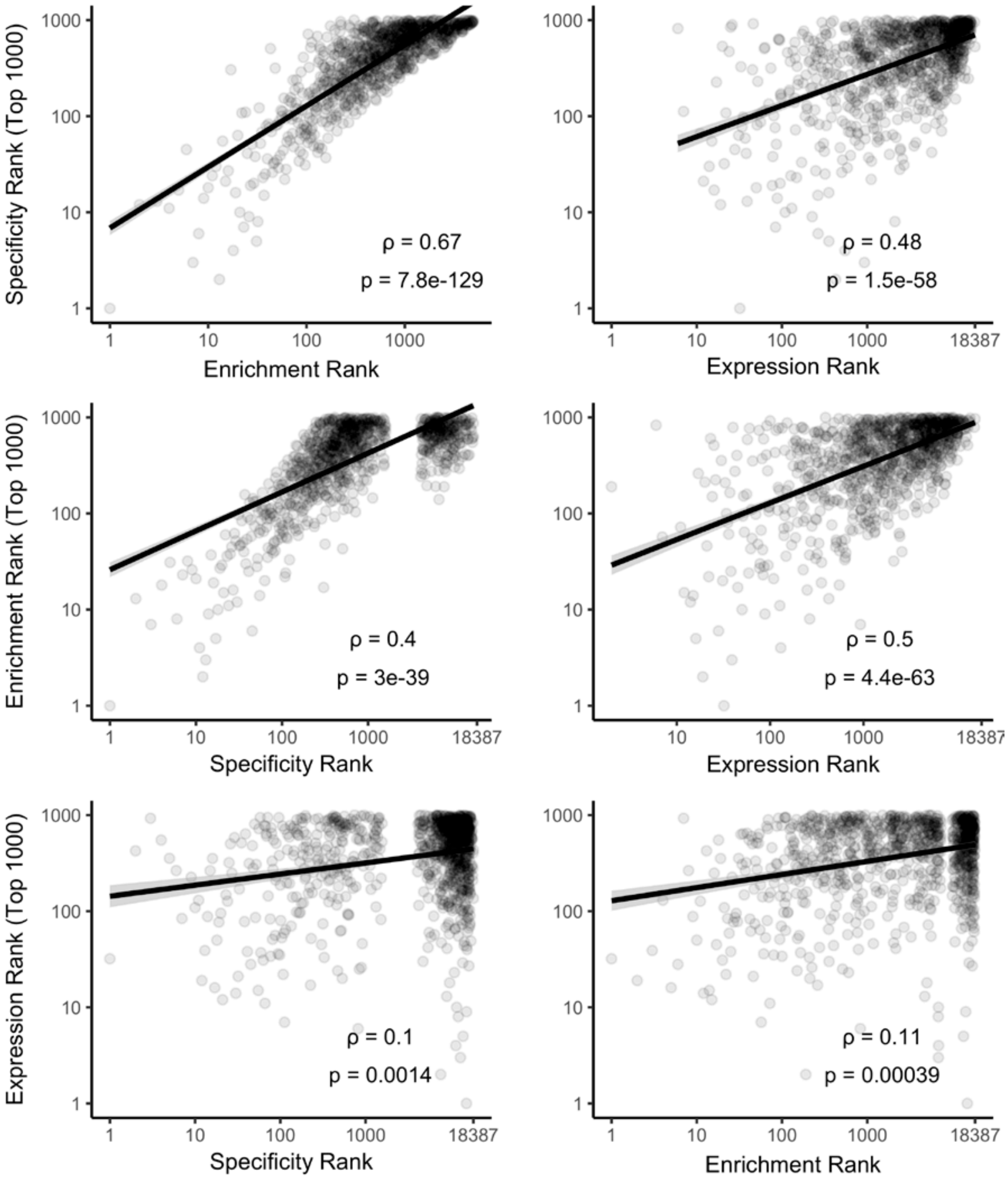

Supplementary Figure 5. Comparison of the three cell type-associated measures in astrocytes. The ranks of the top 1000 genes in each of the three cell type-associated measures (y-axis) compared to the ranks of the other two measures (x-axes; see the Supplementary Methods for more information). Each point represents the ranks of one gene according to each measure. The points are partially transparent (alpha = 0.1) to mitigate overplotting. The black line is a result of a linear model fit to the data, while the grey lines represent 95%

confidence intervals. Note that the discontinuities present in the plots of specificity and enrichment rankings are due to the set of genes with no measured expression in this cell type. The rank correlation and associated p-value for each comparison are noted in the bottom right of each plot.

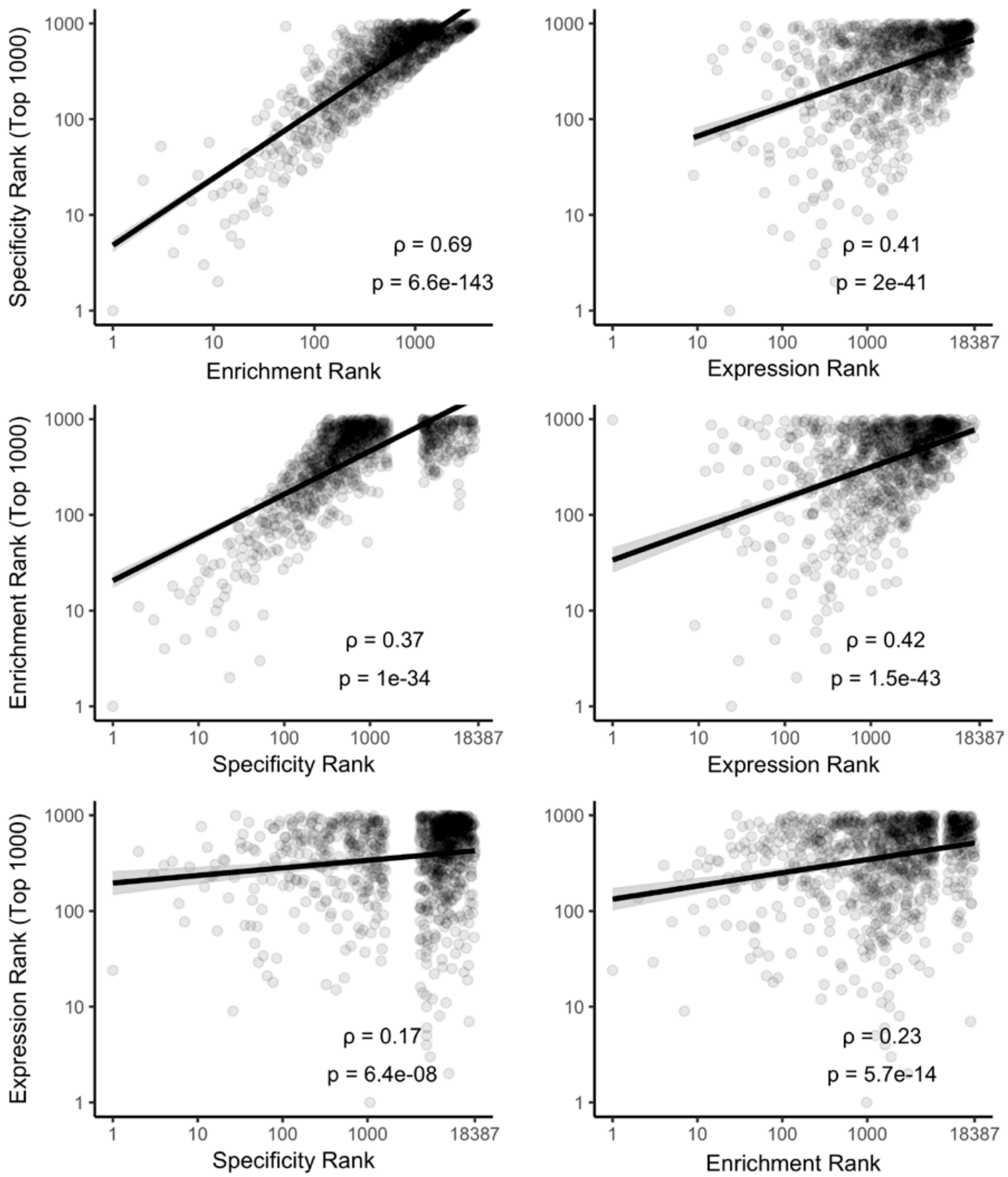

Supplementary Figure 6. Comparison of the three cell type-associated measures in endothelial cells. The ranks of the top 1000 genes in each of the three cell type-associated measures (y-axis) compared to the ranks of the other two measures (x-axes; see the Supplementary Methods for more information). Each point represents the rank of one gene according to each measure. The points are partially transparent (alpha = 0.1) to mitigate overplotting. The black line is a result of a linear model fit to the data, while the grey lines represent 95% confidence intervals. Note that the discontinuities present in the plots of specificity and enrichment rankings are due to the set of genes with no measured expression in this cell type. The rank correlation and associated p-values for each comparison are noted in the bottom right of each plot.

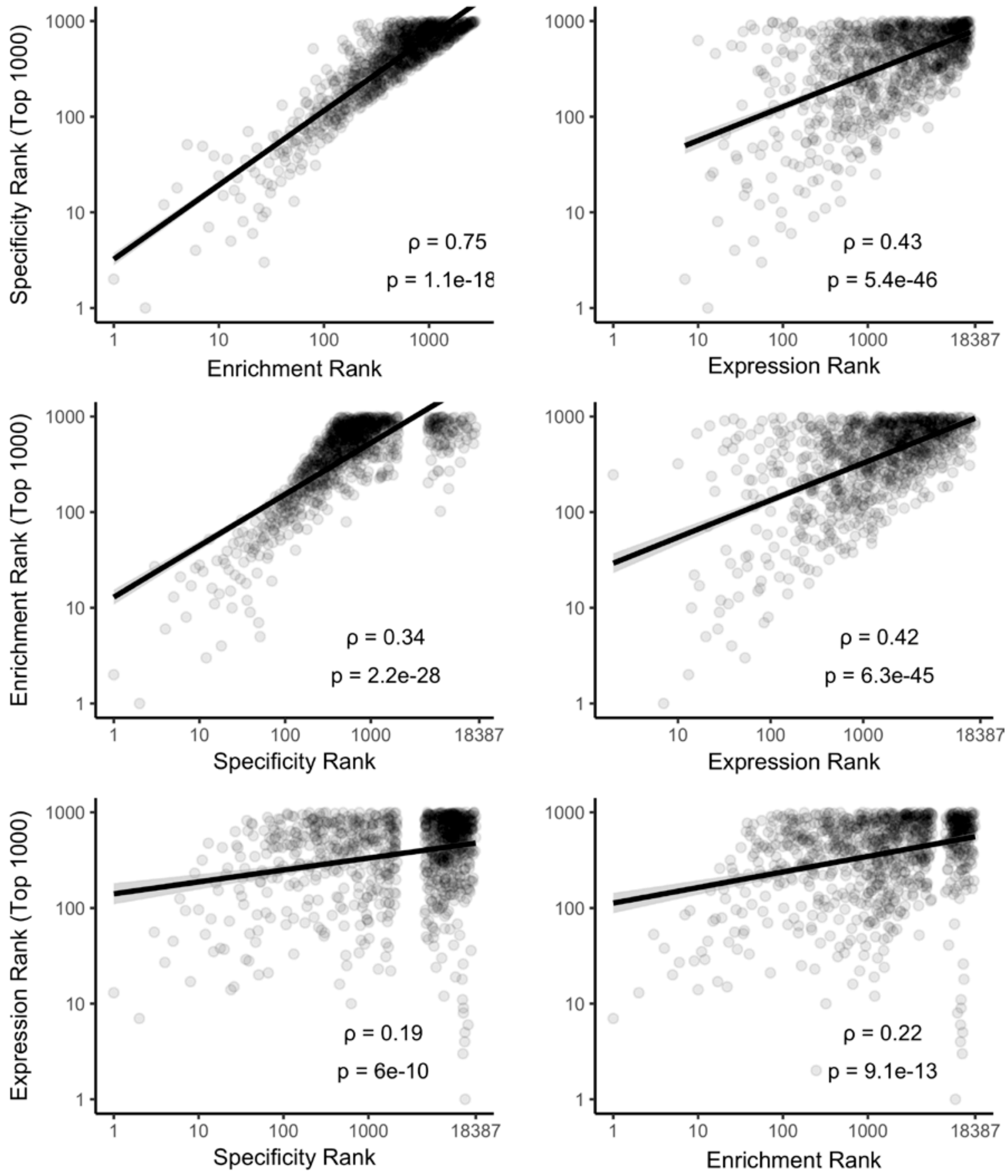

Supplementary Figure 7. Comparison of the three cell type-associated measures in microglia. The ranks of the top 1000 genes in each of the three cell type-associated measures (y-axis) compared to the ranks of the other two measures (x-axes; see the Supplementary Methods for more information). Each point represents the rank of one gene according to each measure. The points are partially transparent (alpha = 0.1) to mitigate overplotting. The black line is a result of a linear model fit to the data, while the grey lines represent 95% confidence intervals. Note that the discontinuities present in the plots of specificity and enrichment rankings are due to the set of genes with no measured expression in this cell type. The rank correlation and associated p-values for each comparison are noted in the bottom right of each plot.

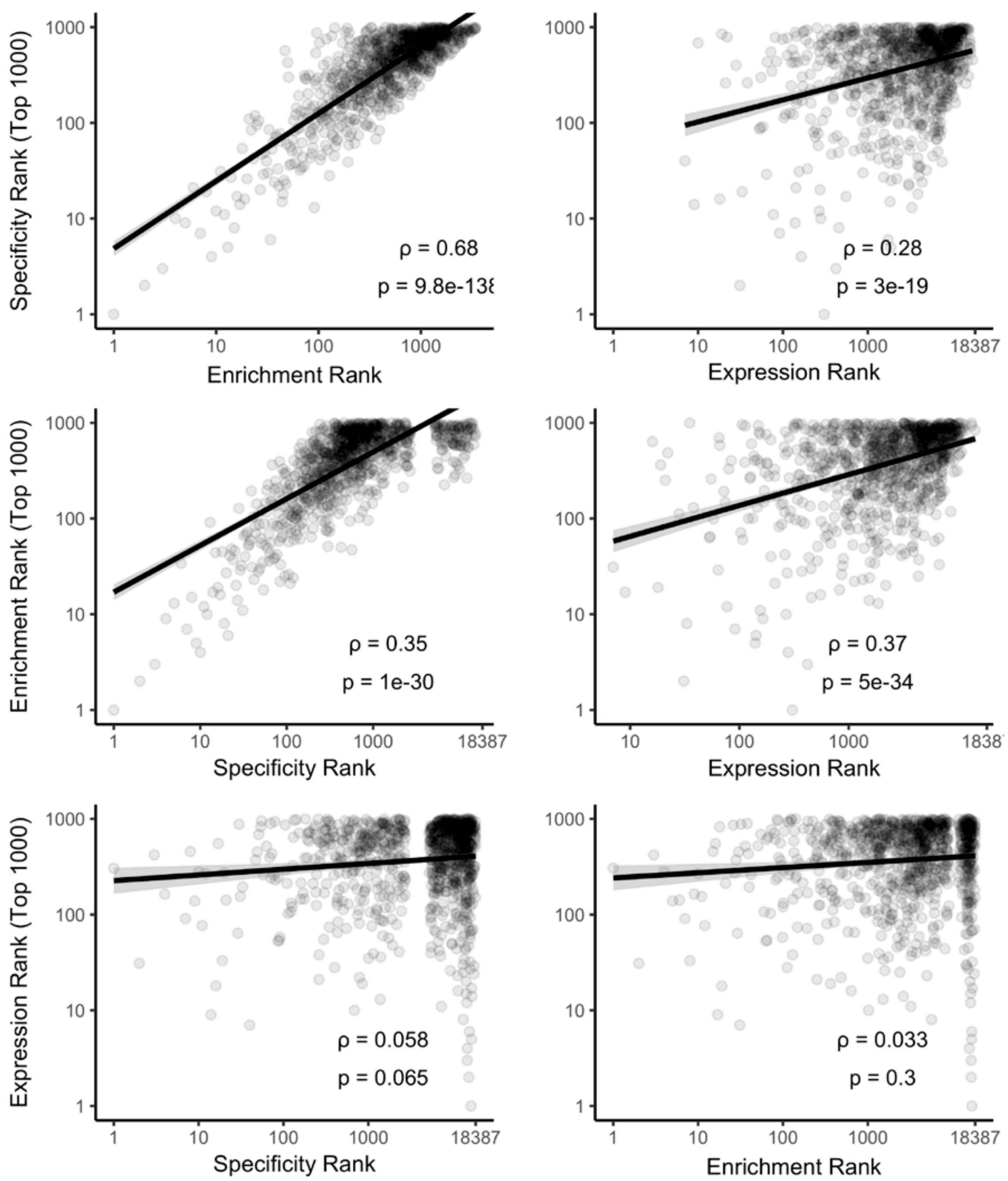

Supplementary Figure 8. Comparison of the three cell type-associated measures in neurons. The ranks of the top 1000 genes in each of the three cell type-associated measures (y-axis) compared to the ranks of the other two measures (x-axes; see the Supplementary Methods for more information). Each point represents the rank of one gene according to each measure. The points are partially transparent (alpha = 0.1) to mitigate overplotting. The black line is a result of a linear model fit to the data, while the grey lines represent 95% confidence intervals. The black line is a result of a linear model fit to the data, while the grey lines represent 95% confidence intervals. Note that the discontinuities present in the plots of specificity and enrichment rankings are due to the set of genes with no measured expression in this cell type. The rank correlation and associated p-values for each comparison are noted in the bottom right of each plot.

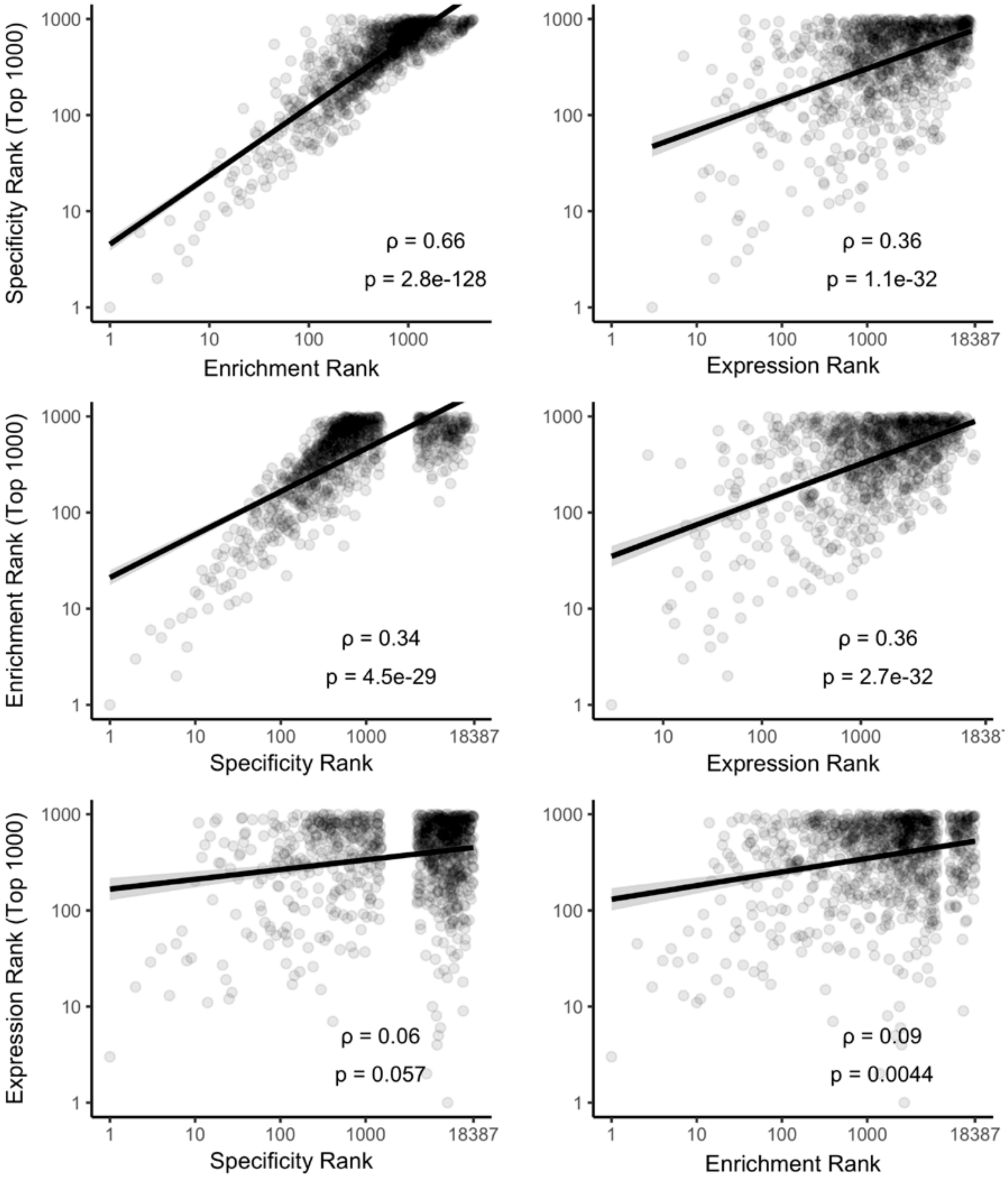

Supplementary Figure 9. Comparison of the three cell type-associated measures in oligodendrocytes. The ranks of the top 1000 genes in each of the three cell type-associated measures (y-axis) compared to the ranks of the other two measures (x-axes; see the Supplementary Methods for more information). Each point represents the rank of one gene according to each measure. The points are partially transparent (alpha = 0.1) to mitigate overplotting. The black line is a result of a linear model fit to the data, while the grey lines represent 95% confidence intervals. Note that the discontinuities present in the plots of specificity and enrichment rankings are due to the set of genes with no measured expression in this cell type. The rank correlation and associated p-values for each comparison are noted in the bottom right of each plot.

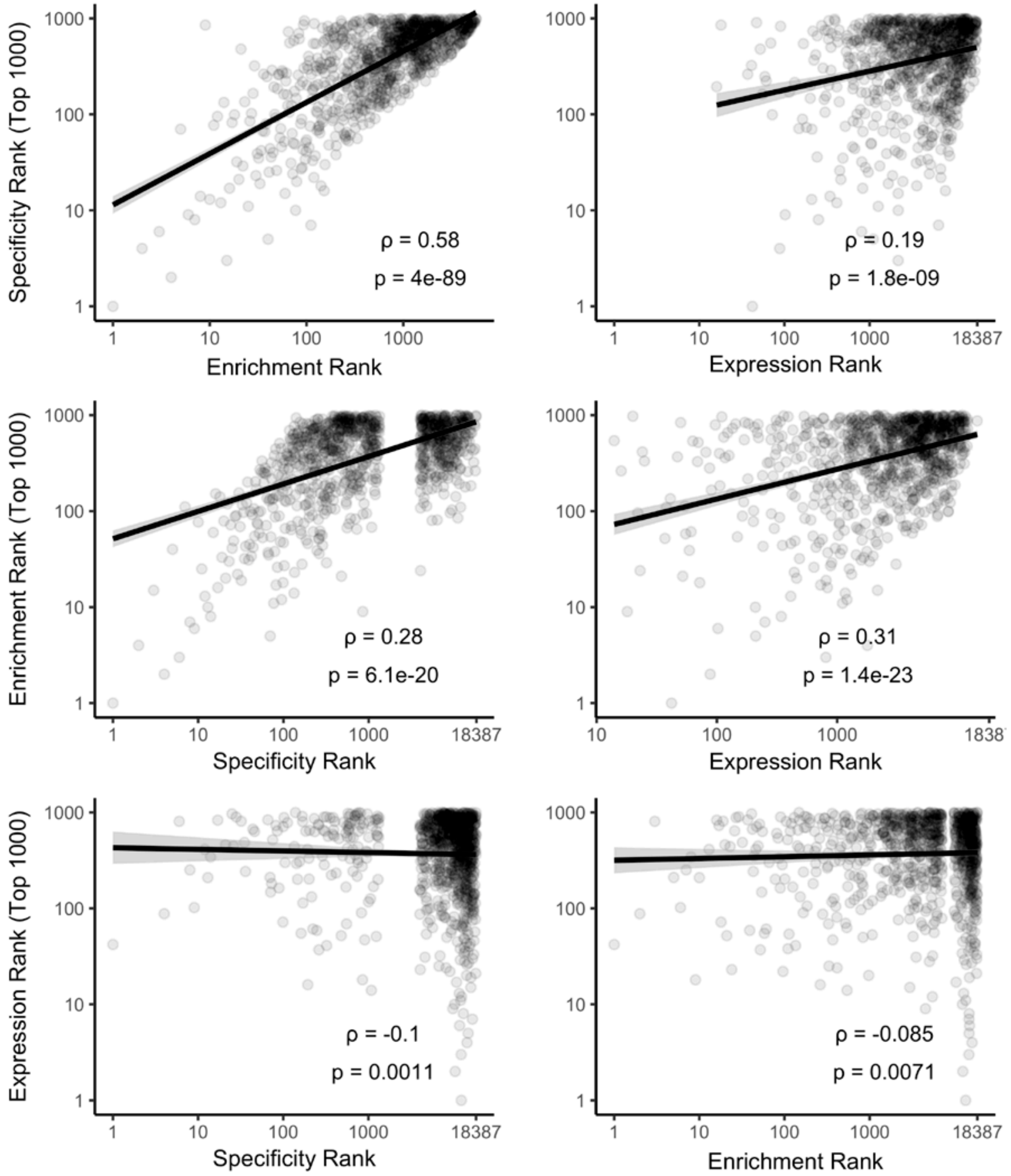

Supplementary Figure 10. Comparison of the three cell type-associated measures in oligodendrocyte precursor cells. The ranks of the top 1000 genes in each of the three cell type-associated measures (y-axis) compared to the ranks of the other two measures (x-axes; see the Supplementary Methods for more information). Each point represents the rank of one gene according to each measure. The points are partially transparent (alpha = 0.1) to mitigate overplotting. The black line is a result of a linear model fit to the data, while the grey lines represent 95% confidence intervals. Note that the discontinuities present in the plots of specificity and enrichment rankings are due to the set of genes with no measured expression in this cell type. The rank correlation and associated p-values for each comparison are noted in the bottom right of each plot.

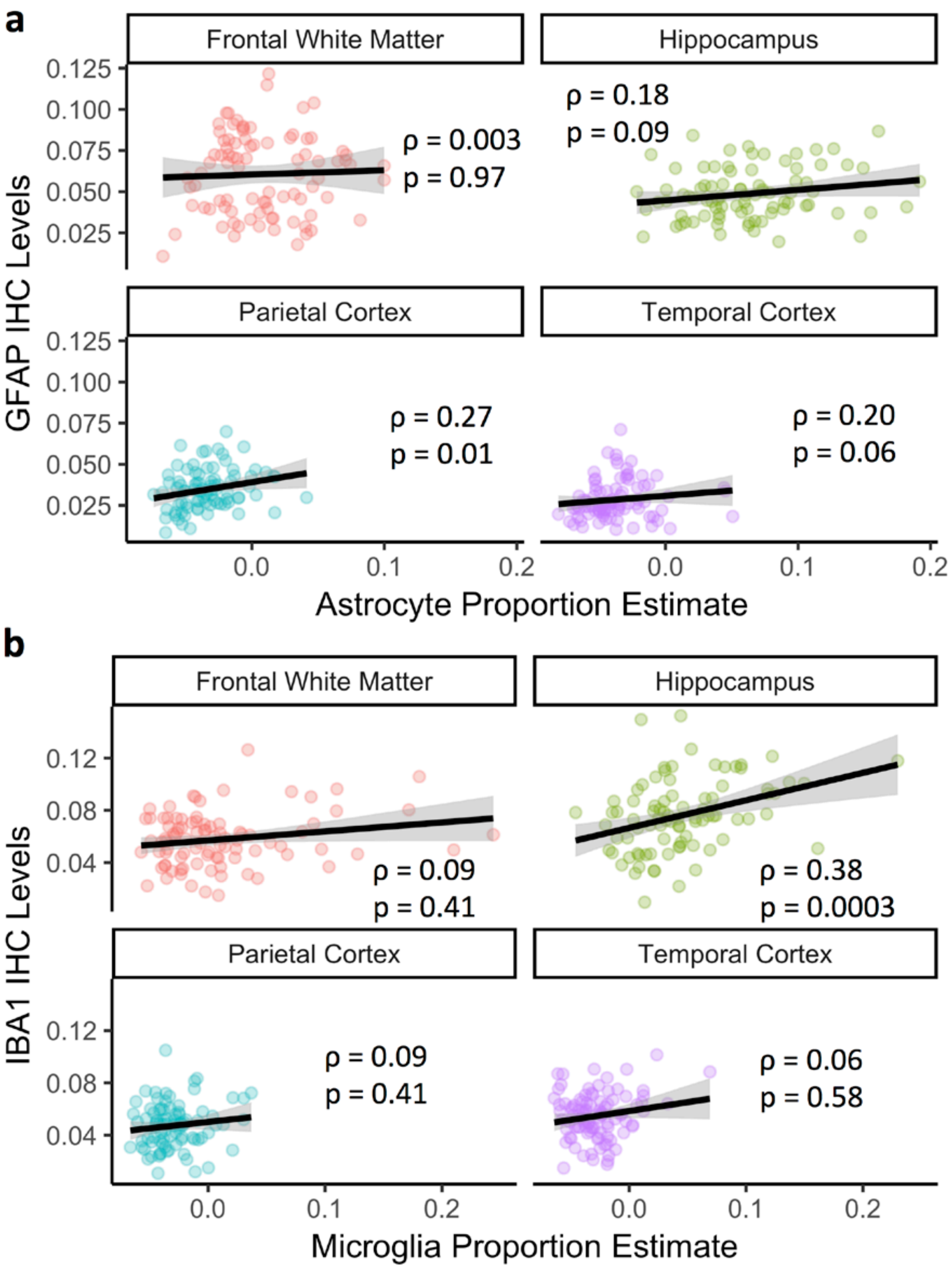

Supplementary Figure 11. Within-region rank correlation between estimated relative cell type proportion and IHC quantifications for astrocytes and microglia.

For each of the four brain regions present in the Allen Brain Atlas Aging, Dementia, and TBI study, we estimated the relative proportion of astrocytes and microglia using an SVD-based method on the top 50 marker genes (x-axis) and found the rank correlation of these estimates with the immunohistochemistry (IHC) quantifications for GFAP and IBA1 (y-axis), respectively. The black line is a result of a linear model fit to the data, while the grey lines represent 95% confidence intervals. FWM, frontal white matter; HIP, hippocampus; PCx, parietal cortex; TCx, temporal cortex.

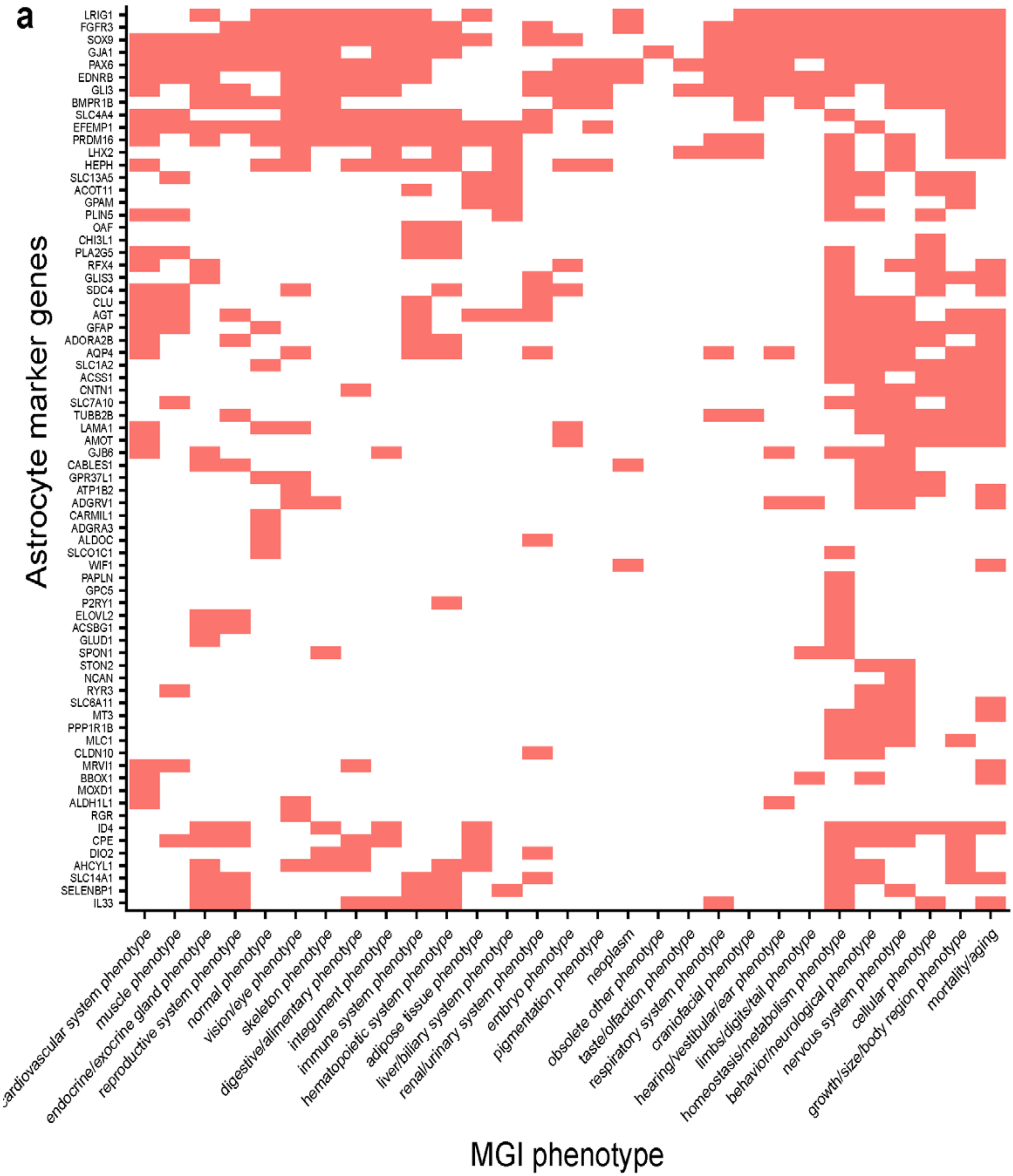

a
Astrocyte marker genes
LRIG1
FGFR3
SOX9
GJA1
PAX6
EDNRB
GLI3
BMPR1B
SLC4A4
EFEMP1
PRDM16
LHX2
HEPH
SLC13A5
ACOT11
GPAM
PLIN5
OAF
CHI3L1
PLA2G5
RFX4
GLIS3
SDC4
CLU
AGT
GFAP
ADORA2B
AQP4
SLC1A2
ACSS1
CNTN1
SLC7A10
TUBB2B
LAMA1
AMOT
GJB6
CABLES1
GPR37L1
ATP1B2
ADGRV1
CARMIL1
ADGRA3
ALDOC
SLCO1C1
WIF1
PAPLN
GPC5
P2RY1
ELOVL2
ACSBG1
GLUD1
SPON1
STON2
NCAN
RYR3
SLC6A11
MT3
PPP1R1B
MLC1
CLDN10
MRVI1
BBOX1
MOXD1
ALDH1L1
RGR
ID4
CPE
DIO2
AHCYL1
SLC14A1
SELENBP1
IL33
ardiovascular system phenotype
muscle phenotype
endocrine/exocrine gland phenotype
reproductive system phenotype
normal phenotype
vision/eye phenotype
skeleton phenotype
digestive/alimentary phenotype
integument phenotype
immune system phenotype
hematopoietic system phenotype
adipose tissue phenotype
liver/biliary system phenotype
renal/urinary system phenotype
embryo phenotype
pigmentation phenotype
neoplasm
obsolete other phenotype
taste/olfaction phenotype
respiratory system phenotype
craniofacial phenotype
hearing/vestibular/ear phenotype
limbs/digits/tail phenotype
homeostasis/metabolism phenotype
behavior/neurological phenotype
nervous system phenotype
cellular phenotype
growth/size/body region phenotype
mortality/aging
MGI phenotype

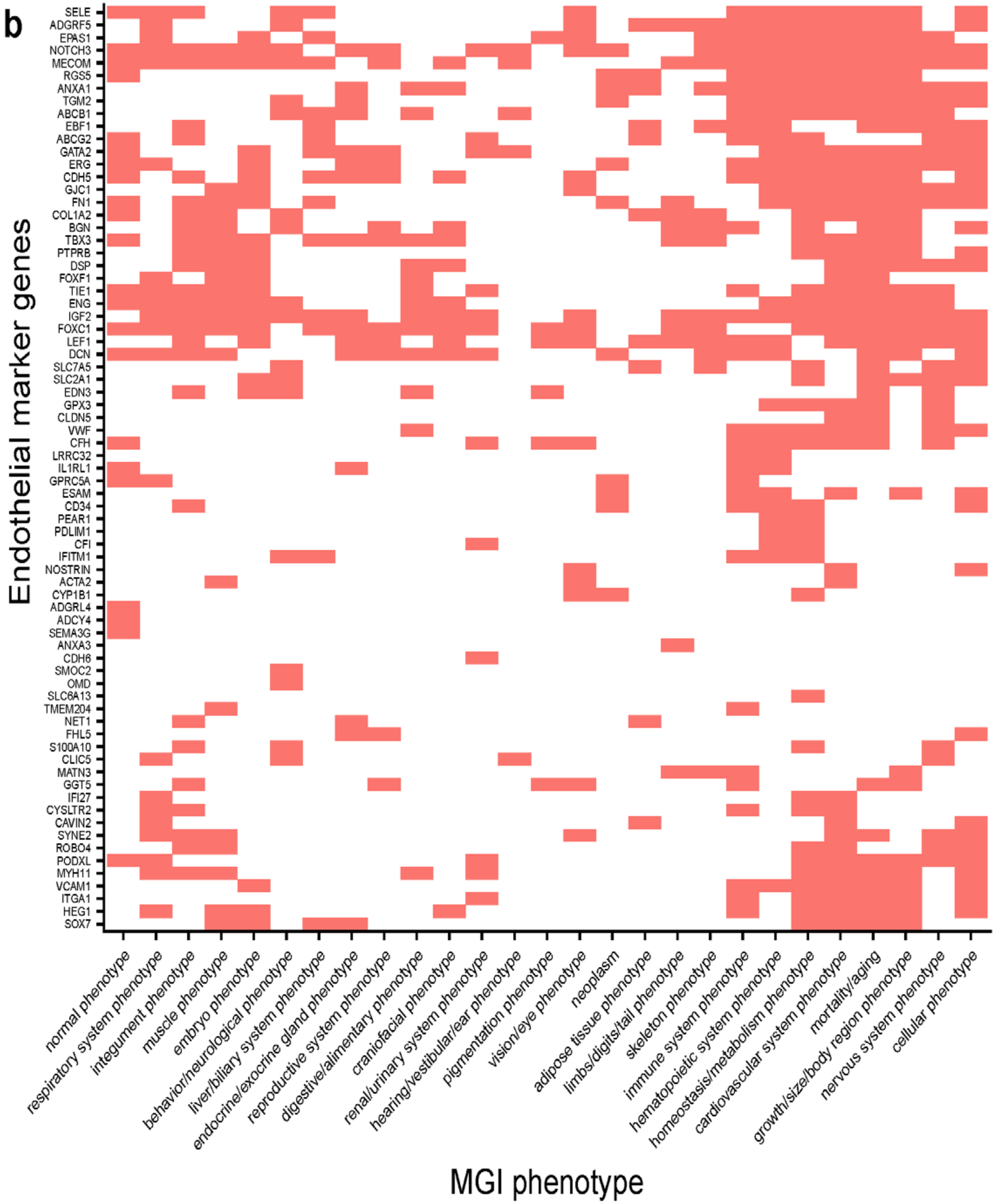
b
Endothelial marker genes
SELE
ADGRF5
EPAS1
NOTCH3
MECOM
RGS5
ANXA1
TGM2
ABCB1
EBF1
ABCG2
GATA2
ERG
CDH5
GJC1
FN1
COL1A2
BGN
TBX3
PTPRB
DSP
FOXF1
TIE1
ENG
IGF2
FOXC1
LEF1
DCN
SLC7A5
SLC2A1
EDN3
GPX3
CLDN5
VWF
CFH
LRRC32
IL1RL1
GPRC5A
ESAM
CD34
PEAR1
PDLIM1
CFI
IFITM1
NOSTRIN
ACTA2
CYP1B1
ADGRL4
ADCY4
SEMA3G
ANXA3
CDH6
SMOC2
OMD
SLC6A13
TMEM204
NET1
FHL5
S100A10
CLIC5
MATN3
GGT5
IFI27
CYSLTR2
CAVIN2
SYNE2
ROBO4
PODXL
MYH11
VCAM1
ITGA1
HEG1
SOX7
normal phenotype
respiratory system phenotype
integument phenotype
muscle phenotype
embryo phenotype
behavior/neurological phenotype
liver/biliary system phenotype
endocrine/exocrine gland phenotype
reproductive system phenotype
digestive/alimentary phenotype
craniofacial phenotype
renal/urinary system phenotype
hearing/vestibular/ear phenotype
pigmentation phenotype
vision/eye phenotype
neoplasm
adipose tissue phenotype
limbs/digits/tail phenotype
skeleton phenotype
immune system phenotype
hematopoietic system phenotype
homeostasis/metabolism phenotype
cardiovascular system phenotype
mortality/aging
growth/size/body region phenotype
nervous system phenotype
cellular phenotype
MGI phenotype

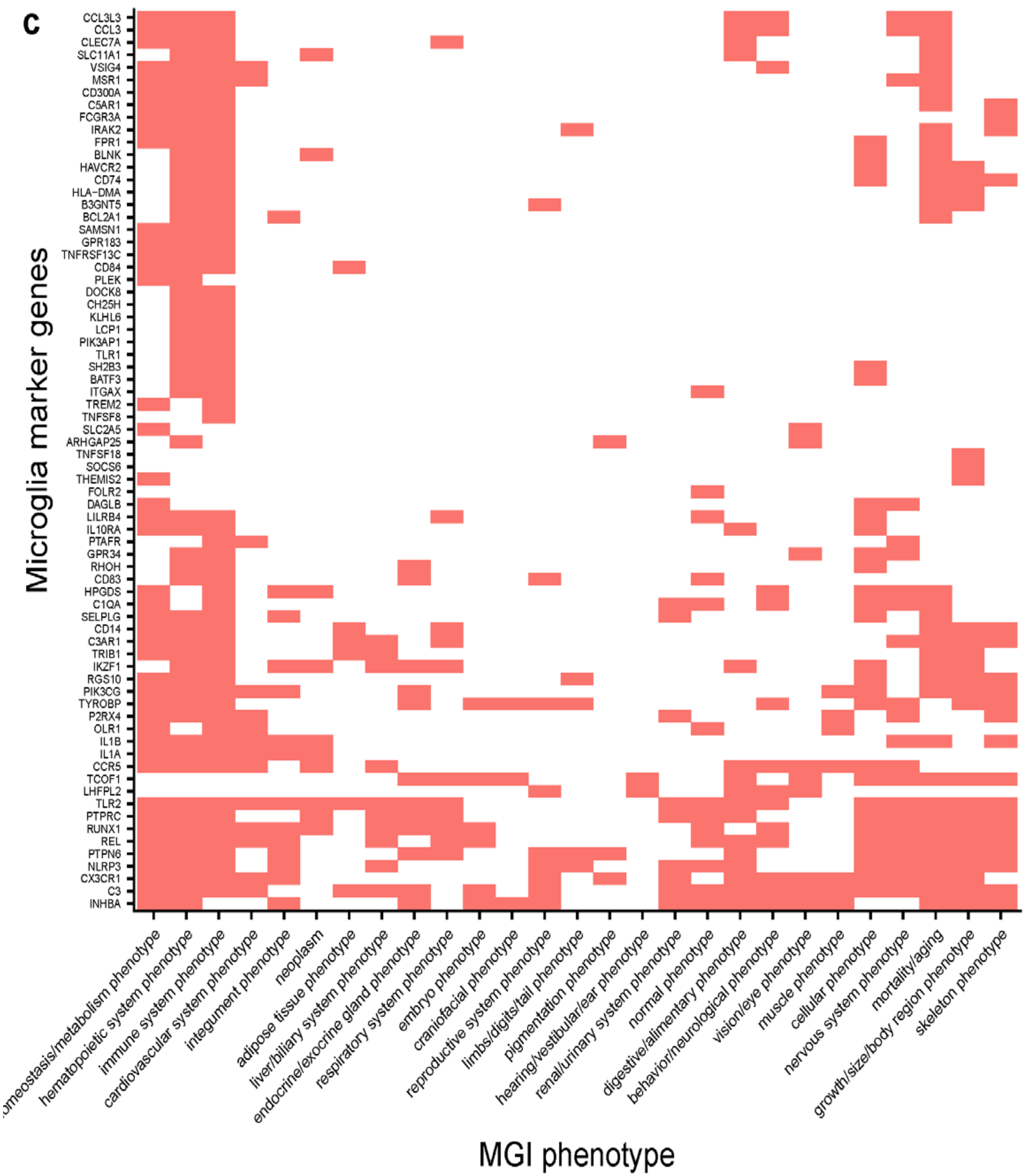
c
Microglia marker genes
CCL3L3
CCL3
CLEC7A
SLC11A1
VSIG4
MSR1
CD300A
C5AR1
FCGR3A
IRAK2
FPR1
BLNK
HAVCR2
CD74
HLA-DMA
B3GNT5
BCL2A1
SAMSN1
GPR183
TNFRSF13C
CD84
PLEK
DOCK8
CH25H
KLHL6
LCP1
PIK3AP1
TLR1
SH2B3
BATF3
ITGAX
TREM2
TNFSF8
SLC2A5
ARHGAP25
TNFSF18
SOCS6
THEMIS2
FOLR2
DAGLB
LILRB4
IL10RA
PTAFR
GPR34
RHOH
CD83
HPGDS
C1QA
SELPLG
CD14
C3AR1
TRIB1
IKZF1
RGS10
PIK3CG
TYROBP
P2RX4
OLR1
IL1B
IL1A
CCR5
TCOF1
LHFPL2
TLR2
PTPRC
RUNX1
REL
PTPN6
NLRP3
CX3CR1
C3
INHBA
homeostasis/metabolism phenotype
hematopoietic system phenotype
immune system phenotype
cardiovascular system phenotype
integument phenotype
neoplasm
adipose tissue phenotype
liver/biliary system phenotype
endocrine/exocrine gland phenotype
respiratory system phenotype
embryo phenotype
craniofacial phenotype
reproductive system phenotype
limbs/digits/tail phenotype
pigmentation phenotype
hearing/vestibular/ear phenotype
renal/urinary system phenotype
normal phenotype
digestive/alimentary phenotype
behavior/neurological phenotype
vision/eye phenotype
muscle phenotype
cellular phenotype
nervous system phenotype
mortality/aging
growth/size/body region phenotype
skeleton phenotype
MGI phenotype

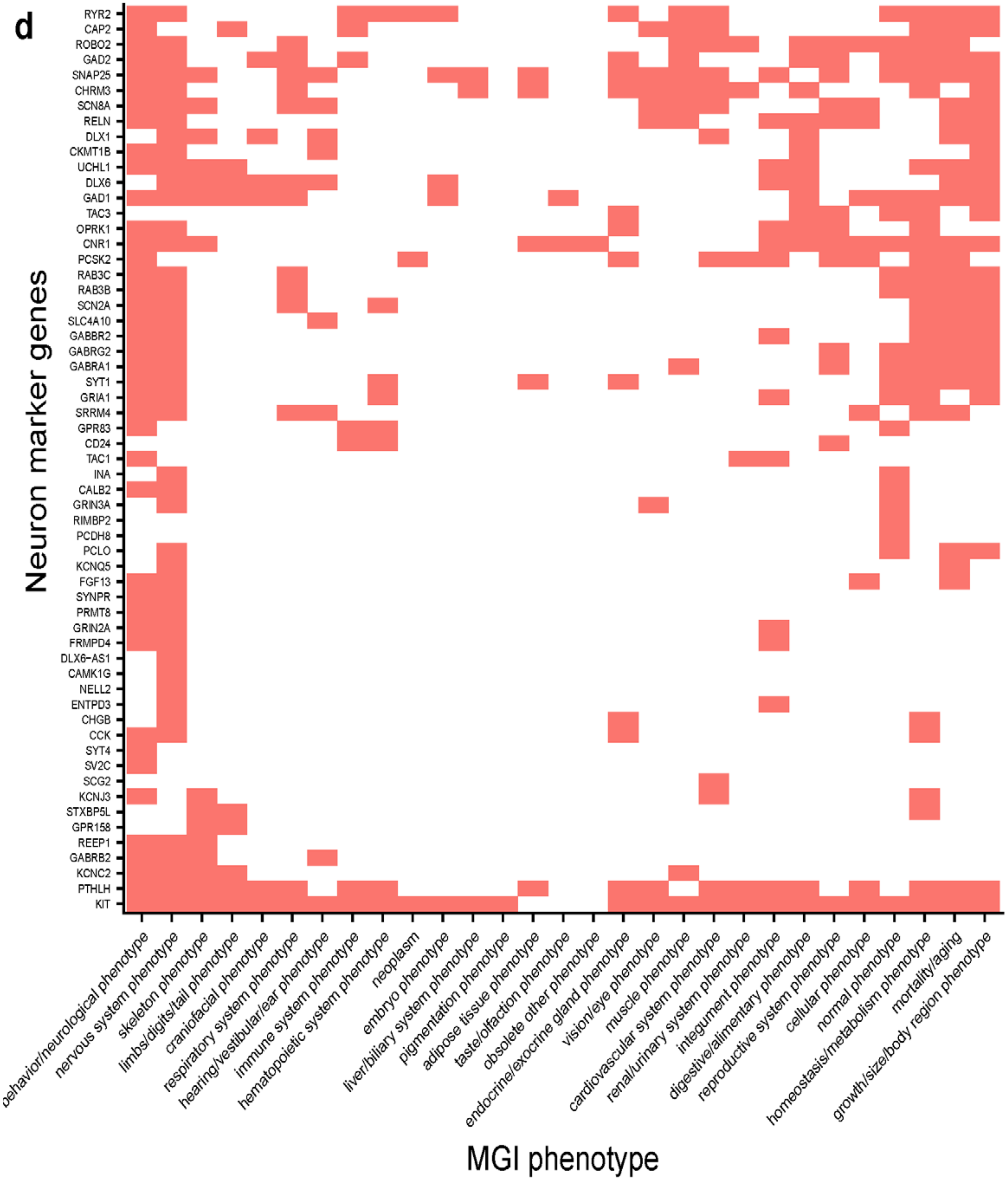
d
Neuron marker genes
RYR2
CAP2
ROBO2
GAD2
SNAP25
CHRM3
SCN8A
RELN
DLX1
CKMT1B
UCHL1
DLX6
GAD1
TAC3
OPRK1
CNR1
PCSK2
RAB3C
RAB3B
SCN2A
SLC4A10
GABBR2
GABRG2
GABRA1
SYT1
GRIA1
SRRM4
GPR83
CD24
TAC1
INA
CALB2
GRIN3A
RIMBP2
PCDH8
PCLO
KCNQ5
FGF13
SYNPR
PRMT8
GRIN2A
FRMPD4
DLX6−AS1
CAMK1G
NELL2
ENTPD3
CHGB
CCK
SYT4
SV2C
SCG2
KCNJ3
STXBP5L
GPR158
REEP1
GABRB2
KCNC2
PTHLH
KIT
behavior/neurological phenotype
nervous system phenotype
skeleton phenotype
limbs/digits/tail phenotype
craniofacial phenotype
respiratory system phenotype
hearing/vestibular/ear phenotype
immune system phenotype
hematopoietic system phenotype
neoplasm
embryo phenotype
liver/biliary system phenotype
pigmentation phenotype
adipose tissue phenotype
taste/olfaction phenotype
obsolete other phenotype
endocrine/exocrine gland phenotype
vision/eye phenotype
muscle phenotype
cardiovascular system phenotype
renal/urinary system phenotype
integument phenotype
digestive/alimentary phenotype
reproductive system phenotype
cellular phenotype
normal phenotype
homeostasis/metabolism phenotype
mortality/aging
growth/size/body region phenotype
MGI phenotype

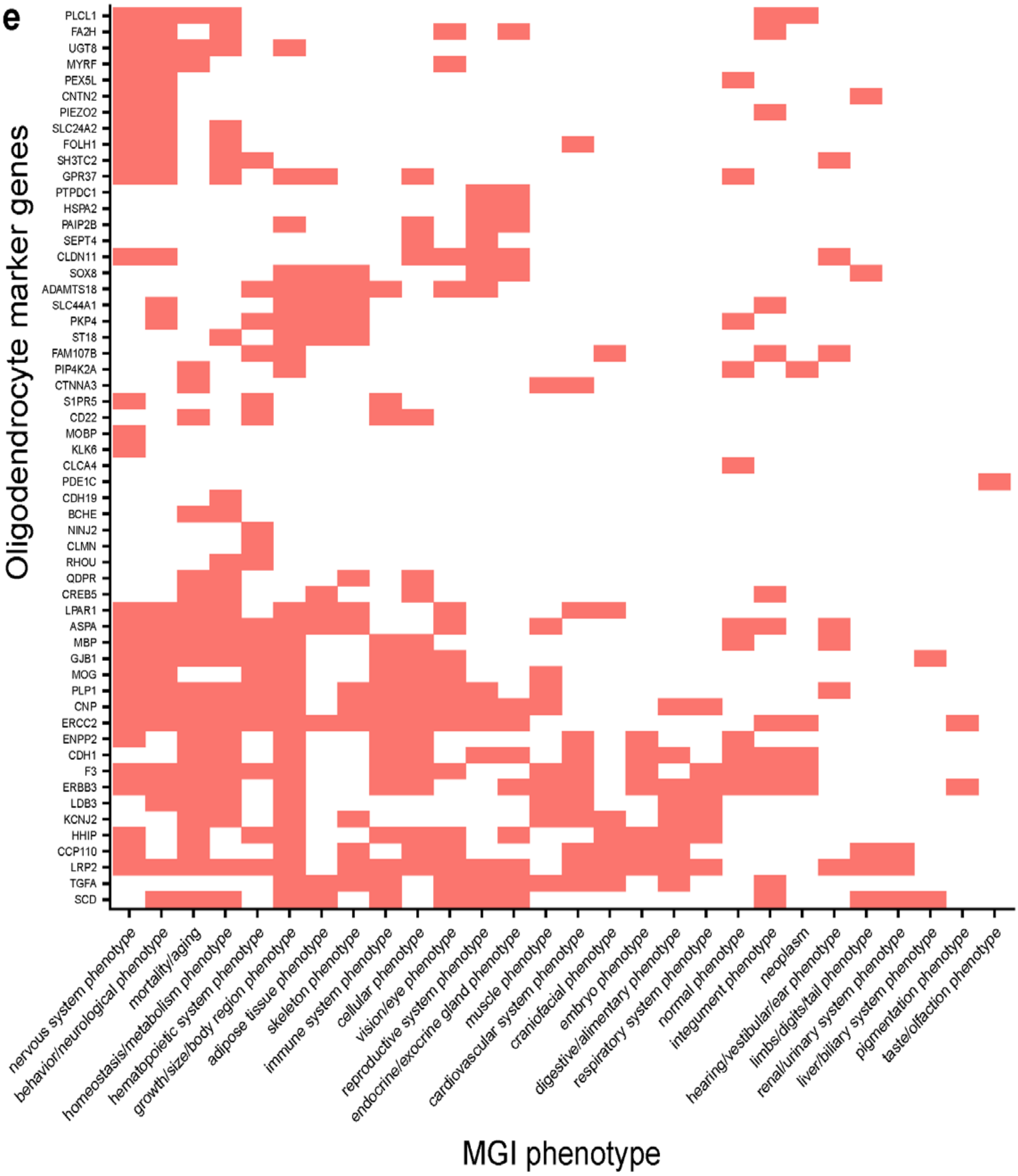

Supplementary Figure 12. MGI phenotypes associated with the brain cell type marker genes.

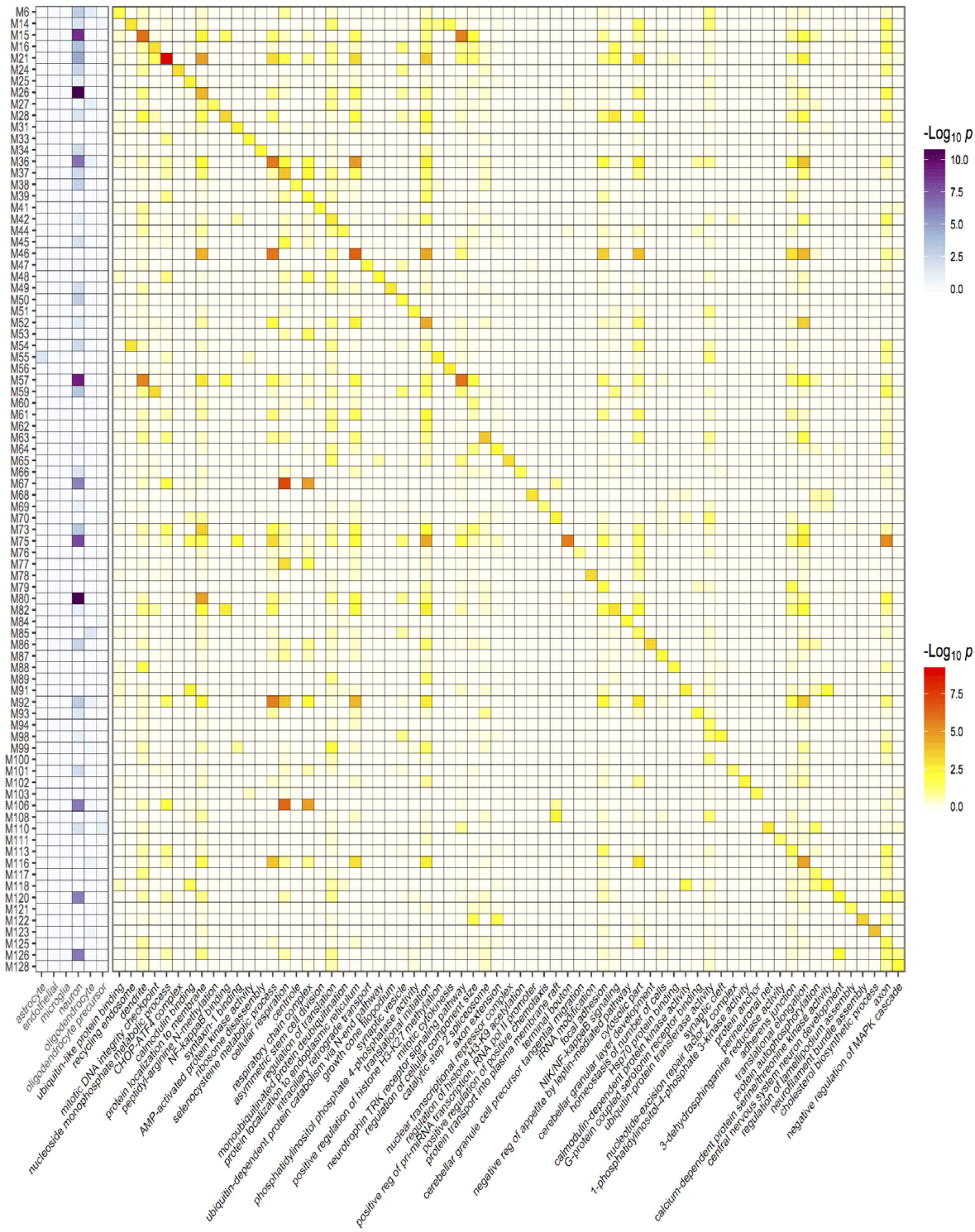

M6
M14
M15
M16
M21
M24
M25
M26
M27
M28
M31
M33
M34
M36
M37
M38
M39
M41
M42
M44
M45
M46
M47
M48
M49
M50
M51
M52
M53
M54
M55
M56
M57
M59
M60
M61
M62
M63
M64
M65
M66
M67
M68
M69
M70
M73
M75
M76
M77
M78
M79
M80
M82
M84
M85
M86
M87
M88
M89
M91
M92
M93
M94
M98
M99
M100
M101
M102
M103
M106
M108
M110
M111
M113
M116
M117
M118
M120
M121
M122
M123
M125
M126
M128
astrocyte
endothelial
microglia
neuron
oligodendrocyte
oligodendrocyte precursor
ubiquitin-like protein binding
recycling endosome
dendrite
mitotic DNA integrity checkpoint
nucleoside monophosphate metabolic process
CHOP-ATF4 complex
calmodulin binding
protein localization to membrane
peptidyl-arginine N-methylation
NF-kappaB binding
AMP-activated protein kinase activity
syntaxin-1 binding
ribosome disassembly
selenocysteine metabolic process
cellular respiration
centriole
respiratory chain complex
asymmetric stem cell division
regulation of translation
monoubiquitinated protein deubiquitination
protein localization to endoplasmic reticulum
ubiquitin-dependent protein catabolism via N-end pathway
intraciliary retrograde transport
growth cone filopodium
phosphatidylinositol phosphate 4-phosphatase activity
synaptic vesicle
positive regulation of translational initiation
positive regulation of histone H3-K27 methylation
mitotic cytokinesis
neurotrophin TRK receptor signaling pathway
regulation of cellular component size
catalytic step 2 spliceosome
axon extension
nuclear transcriptional repressor complex
regulation of histone H3-K9 acetylation
positive reg of pri-miRNA transcription, RNA pol II promoter
positive regulation of positive chemotaxis
protein transport into plasma membrane raft
terminal bouton
cerebellar granule cell precursor tangential migration
tRNA modification
negative reg of appetite by leptin-mediated pathway
NIK/NF-kappaB signaling
focal adhesion
cytosolic part
cerebellar granular layer development
homeostasis of number of cells
Hsp70 protein binding
calmodulin-dependent protein kinase activity
G-protein coupled serotonin receptor binding
ubiquitin-protein transferase activity
nucleotide-excision repair factor 2 complex
synaptic cleft
1-phosphatidylinositol-4-phosphate 3-kinase activity
protein anchor
3-dehydrosphinganine reductase activity
perineuronal net
adherens junction
translational elongation
calcium-dependent protein serine/threonine kinase activity
protein autophosphorylation
central nervous system neuron development
regulation of lamellipodium assembly
neurofilament bundle assembly
cholesterol biosynthetic process
axon
negative regulation of MAPK cascade
-Log10 p
10.0
7.5
5.0
2.5
0.0
-Log10 p
7.5
5.0
2.5
0.0

Supplementary Figure 13. Enrichments of MEGENA modules from human neurons in brain cell type signatures and GO terms. The modules on the left match the modules in the multiscale network shown in **Figure 8A**. The left panel shows the enrichment (Benjamini-Hochberg adjusted -$\log_{10}$ p-value) of each module in the top 500 most cell type-specific genes for each cell type in humans that we identified in this manuscript. The right panel shows the most significantly enriched GO term (Benjamini-Hochberg adjusted -$\log_{10}$ p-value) for the genes in each of the corresponding modules, along with the GO enrichment of that same GO term in all the other modules.

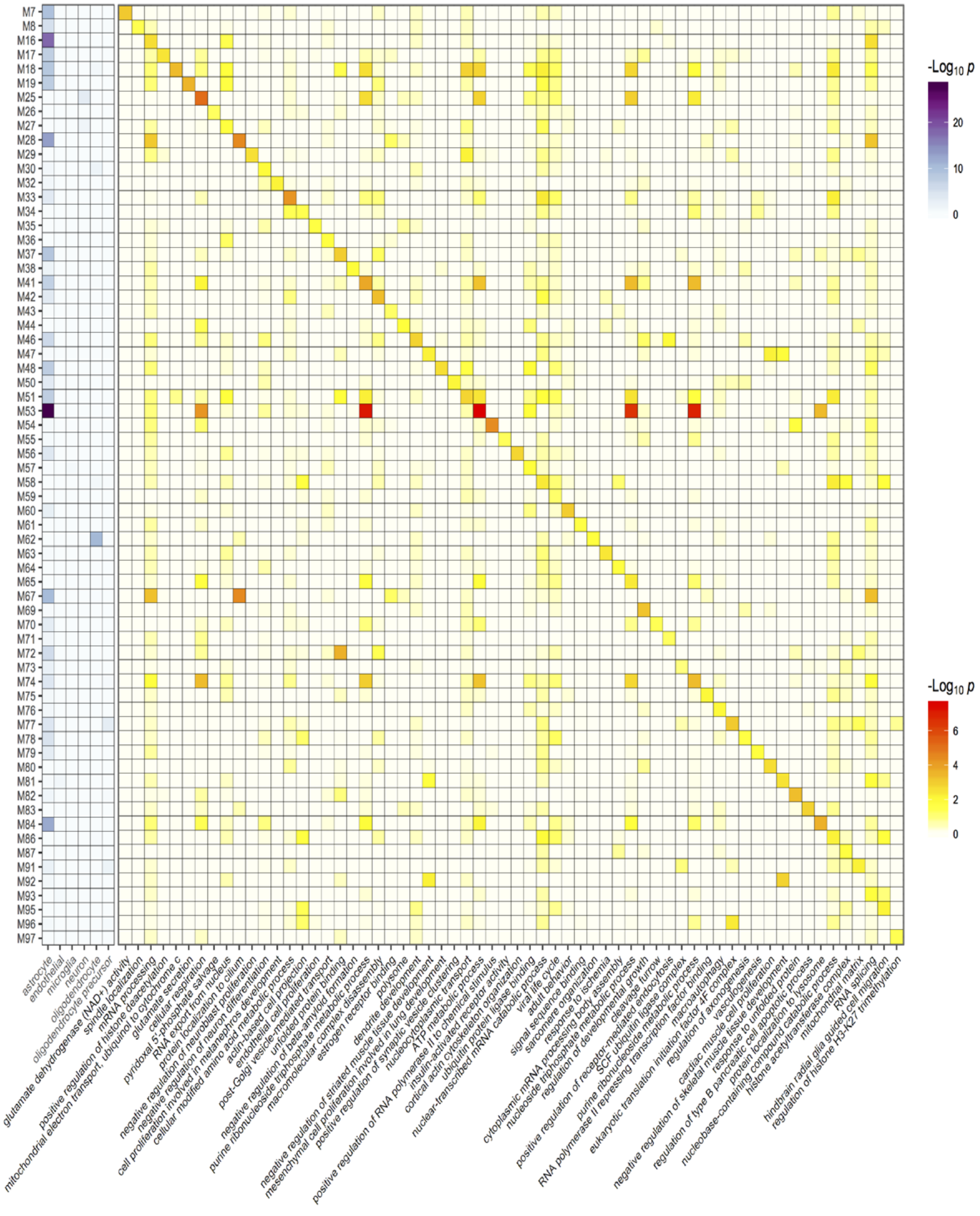

M7
M8
M16
M17
M18
M19
M25
M26
M27
M28
M29
M30
M32
M33
M34
M35
M36
M37
M38
M41
M42
M43
M44
M46
M47
M48
M50
M51
M53
M54
M55
M56
M57
M58
M59
M60
M61
M62
M63
M64
M65
M67
M69
M70
M71
M72
M73
M74
M75
M76
M77
M78
M79
M80
M81
M82
M83
M84
M86
M87
M91
M92
M93
M95
M96
M97
astrocyte
endothelial
microglia
neuron
oligodendrocyte
oligodendrocyte precursor
glutamate dehydrogenase (NAD+) activity
spindle localization
mRNA processing
positive regulation of histone deacetylation
mitochondrial electron transport, ubiquinol to cytochrome c
glutamate secretion
cellular respiration
pyridoxal 5'-phosphate salvage
RNA export from nucleus
protein localization to cilium
negative regulation of neuroblast proliferation
negative regulation of neuron differentiation
cell proliferation involved in metanephros development
cellular modified amino acid metabolic process
actin-based cell projection
endothelial cell proliferation
post-Golgi vesicle-mediated transport
unfolded protein binding
negative regulation of beta-amyloid formation
purine ribonucleoside triphosphate metabolic process
macromolecular complex disassembly
estrogen receptor binding
polysome
dendrite development
negative regulation of striated muscle tissue development
mesenchymal cell proliferation involved in lung development
positive regulation of synaptic vesicle clustering
nucleocytoplasmic transport
ATP metabolic process
positive regulation of RNA polymerase II to chemical stimulus
insulin-activated receptor activity
cortical actin cytoskeleton organization
ubiquitin protein ligase binding
nuclear-transcribed mRNA catabolic process
viral life cycle
adult behavior
signal sequence binding
sarcomere organization
response to ischemia
cytoplasmic mRNA processing body assembly
nucleoside triphosphate metabolic process
regulation of developmental growth
cleavage furrow
positive regulation of receptor-mediated endocytosis
SCF ubiquitin ligase complex
purine ribonucleoside metabolic process
RNA polymerase II repressing transcription factor binding
macroautophagy
eukaryotic translation initiation factor 4F complex
regulation of axonogenesis
vasculogenesis
cardiac muscle cell proliferation
negative regulation of skeletal muscle tissue development
response to unfolded protein
regulation of type B pancreatic cell apoptotic process
protein localization to lysosome
nucleobase-containing compound catabolic process
histone acetyltransferase complex
mitochondrial matrix
RNA splicing
hindbrain radial glia guided cell migration
regulation of histone H3-K27 trimethylation
$-Log_{10}\ p$
20
10
0
$-Log_{10}\ p$
6
4
2
0

Supplementary Figure 14. Enrichments of MEGENA modules from human astrocytes in brain cell type signatures and GO terms. The left panel shows the enrichment (Benjamini-Hochberg adjusted $-\log_{10}$ p-value) of each module in the top 500 most cell type-specific genes for each cell type in humans that we identified in this manuscript. The right panel shows the most significantly enriched GO term (Benjamini-Hochberg adjusted $-\log_{10}$ p-value) for the genes in each of the corresponding modules, along with the GO enrichment of that same GO term in all the other modules.

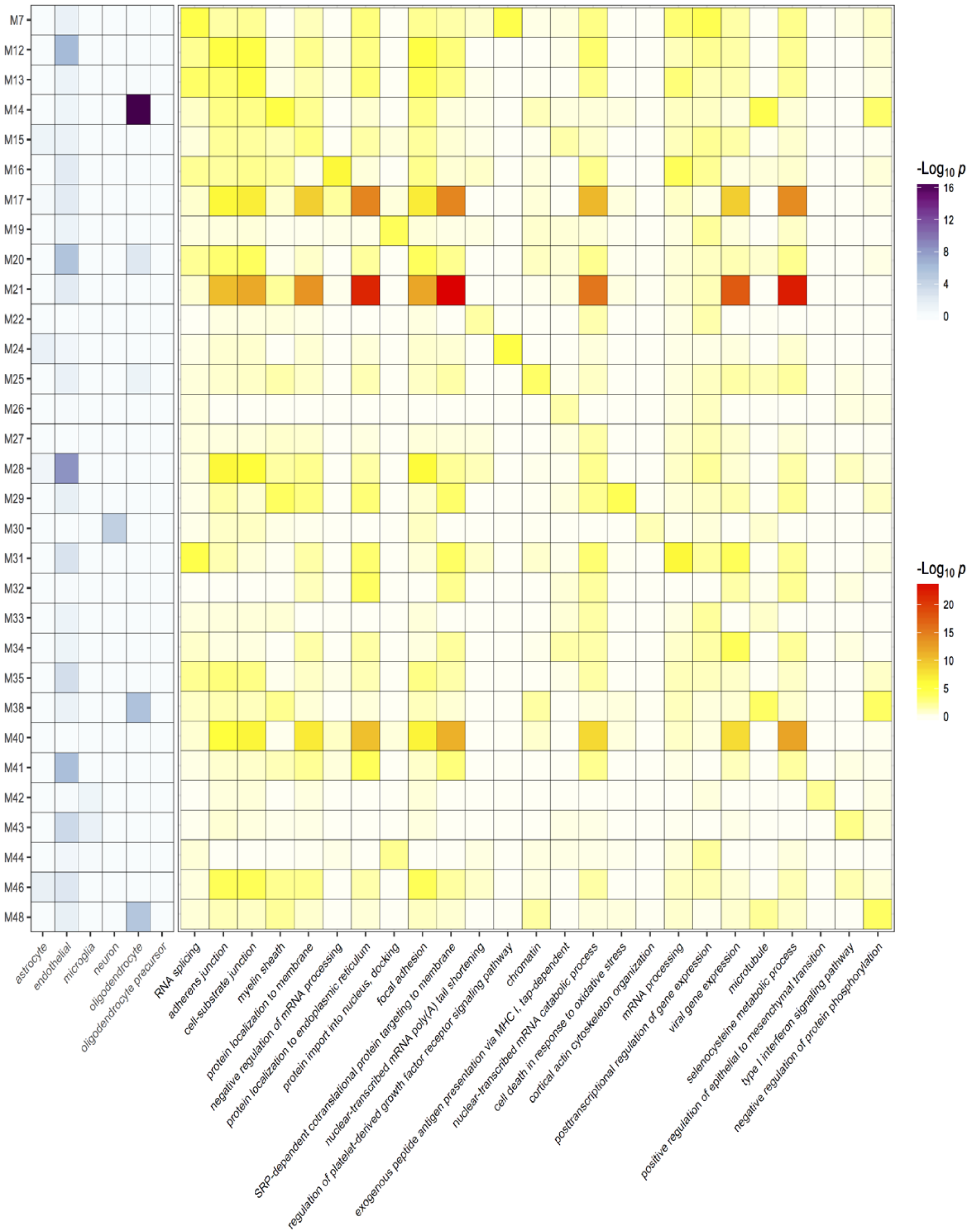
M7
M12
M13
M14
M15
M16
M17
M19
M20
M21
M22
M24
M25
M26
M27
M28
M29
M30
M31
M32
M33
M34
M35
M38
M40
M41
M42
M43
M44
M46
M48
astrocyte
endothelial
microglia
neuron
oligodendrocyte
oligodendrocyte precursor
RNA splicing
adherens junction
cell-substrate junction
myelin sheath
protein localization to membrane
negative regulation of mRNA processing
protein localization to endoplasmic reticulum
protein import into nucleus, docking
focal adhesion
SRP-dependent cotranslational protein targeting to membrane
nuclear-transcribed mRNA poly(A) tail shortening
regulation of platelet-derived growth factor receptor signaling pathway
chromatin
exogenous peptide antigen presentation via MHC I, tap-dependent
nuclear-transcribed mRNA catabolic process
cell death in response to oxidative stress
cortical actin cytoskeleton organization
mRNA processing
posttranscriptional regulation of gene expression
viral gene expression
microtubule
selenocysteine metabolic process
positive regulation of epithelial to mesenchymal transition
type I interferon signaling pathway
negative regulation of protein phosphorylation
-Log10 p
16
12
8
4
0
-Log10 p
20
15
10
5
0

Supplementary Figure 15. Enrichments of MEGENA modules from human endothelial cells in brain cell type signatures and GO terms. The left panel shows the enrichment (Benjamini-Hochberg adjusted -$\log_{10}$ p-value) of each module in the top 500 most cell type-specific genes for each cell type in humans that we identified in this manuscript. The right panel shows the most significantly enriched GO term (Benjamini-Hochberg adjusted -$\log_{10}$ p-value) for the genes in each of the corresponding modules, along with the GO enrichment of that same GO term in all the other modules.

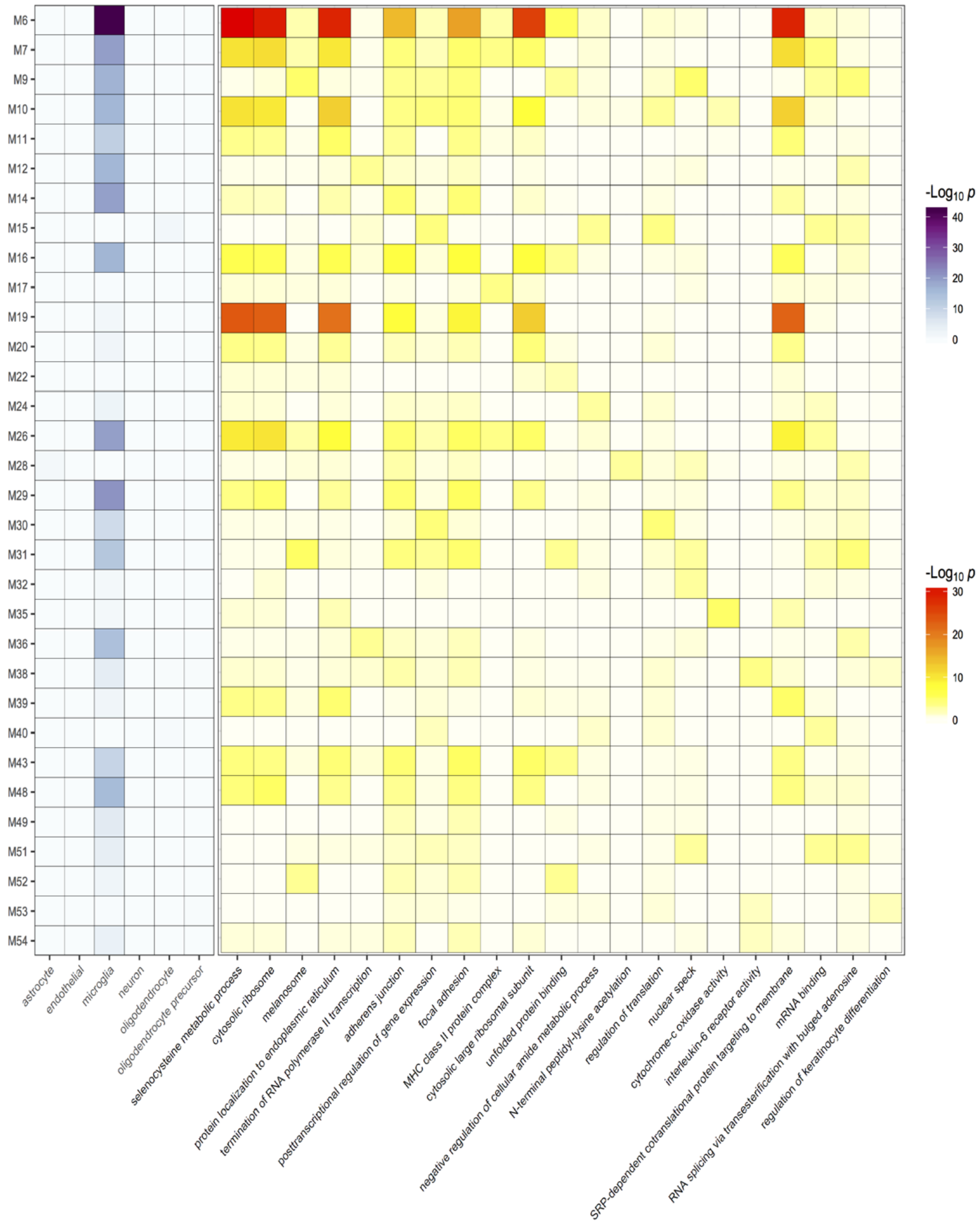
M6
M7
M9
M10
M11
M12
M14
M15
M16
M17
M19
M20
M22
M24
M26
M28
M29
M30
M31
M32
M35
M36
M38
M39
M40
M43
M48
M49
M51
M52
M53
M54
astrocyte
endothelial
microglia
neuron
oligodendrocyte
oligodendrocyte precursor
selenocysteine metabolic process
cytosolic ribosome
melanosome
protein localization to endoplasmic reticulum
termination of RNA polymerase II transcription
adherens junction
posttranscriptional regulation of gene expression
focal adhesion
MHC class II protein complex
cytosolic large ribosomal subunit
unfolded protein binding
negative regulation of cellular amide metabolic process
N-terminal peptidyl-lysine acetylation
regulation of translation
nuclear speck
cytochrome-c oxidase activity
interleukin-6 receptor activity
SRP-dependent cotranslational protein targeting to membrane
mRNA binding
RNA splicing via transesterification with bulged adenosine
regulation of keratinocyte differentiation
$-Log_{10}\ p$
40
30
20
10
0
$-Log_{10}\ p$
30
20
10
0

Supplementary Figure 16. Enrichments of MEGENA modules from human microglia in brain cell type signatures and GO terms. The left panel shows the enrichment (Benjamini-Hochberg adjusted $-\log_{10}$ p-value) of each module in the top 500 most cell type-specific genes for each cell type in humans that we identified in this manuscript. The right panel shows the most significantly enriched GO term (Benjamini-Hochberg adjusted $-\log_{10}$ p-value) for the genes in each of the corresponding modules, along with the GO enrichment of that same GO term in all the other modules.

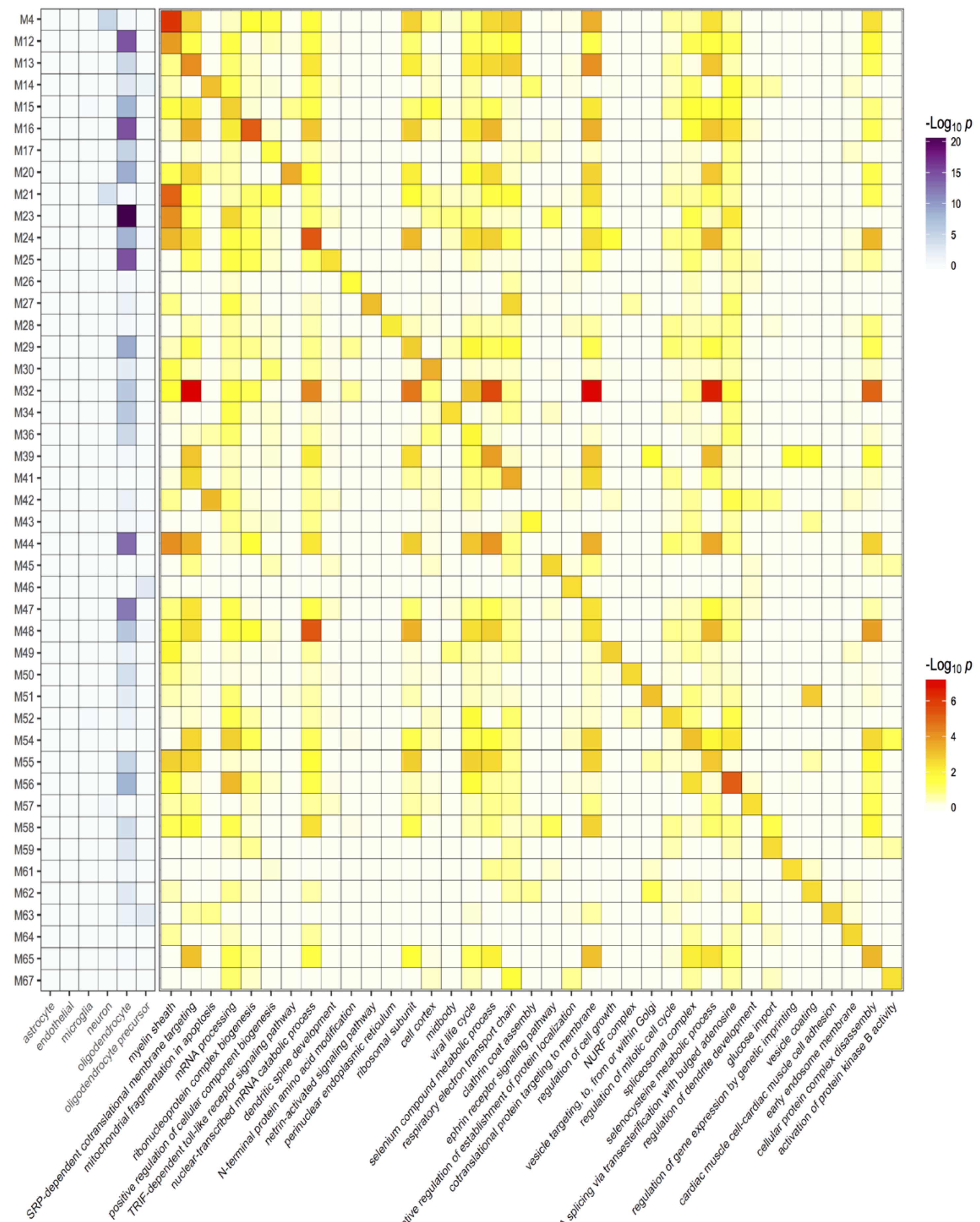
M4
M12
M13
M14
M15
M16
M17
M20
M21
M23
M24
M25
M26
M27
M28
M29
M30
M32
M34
M36
M39
M41
M42
M43
M44
M45
M46
M47
M48
M49
M50
M51
M52
M54
M55
M56
M57
M58
M59
M61
M62
M63
M64
M65
M67
astrocyte
endothelial
microglia
neuron
oligodendrocyte
oligodendrocyte precursor
myelin sheath
SRP-dependent cotranslational membrane targeting
mitochondrial fragmentation in apoptosis
mRNA processing
ribonucleoprotein complex biogenesis
positive regulation of cellular component biogenesis
TRIF-dependent toll-like receptor signaling pathway
nuclear-transcribed mRNA catabolic process
dendritic spine development
N-terminal protein amino acid modification
netrin-activated signaling pathway
perinuclear endoplasmic reticulum
ribosomal subunit
cell cortex
midbody
viral life cycle
selenium compound metabolic process
respiratory electron transport chain
clathrin coat assembly
ephrin receptor signaling pathway
negative regulation of establishment of protein localization
cotranslational protein targeting to membrane
regulation of cell growth
NURF complex
vesicle targeting, to, from or within Golgi
regulation of mitotic cell cycle
spliceosomal complex
selenocysteine metabolic process
RNA splicing via transesterification with bulged adenosine
regulation of dendrite development
glucose import
regulation of gene expression by genetic imprinting
vesicle coating
cardiac muscle cell-cardiac muscle cell adhesion
early endosome membrane
cellular protein complex disassembly
activation of protein kinase B activity
-Log10 p
20
15
10
5
0
-Log10 p
6
4
2
0

Supplementary Figure 17. Enrichments of MEGENA modules from human oligodendrocytes in brain cell type signatures and GO terms. The left panel shows the enrichment (Benjamini-Hochberg adjusted -$\log_{10}$ p-value) of each module in the top 500 most cell type-specific genes for each cell type in humans that we identified in this manuscript. The right panel shows the most significantly enriched GO term (Benjamini-Hochberg adjusted -$\log_{10}$ p-value) for the genes in each of the corresponding modules, along with the GO enrichment of that same GO term in all the other modules.

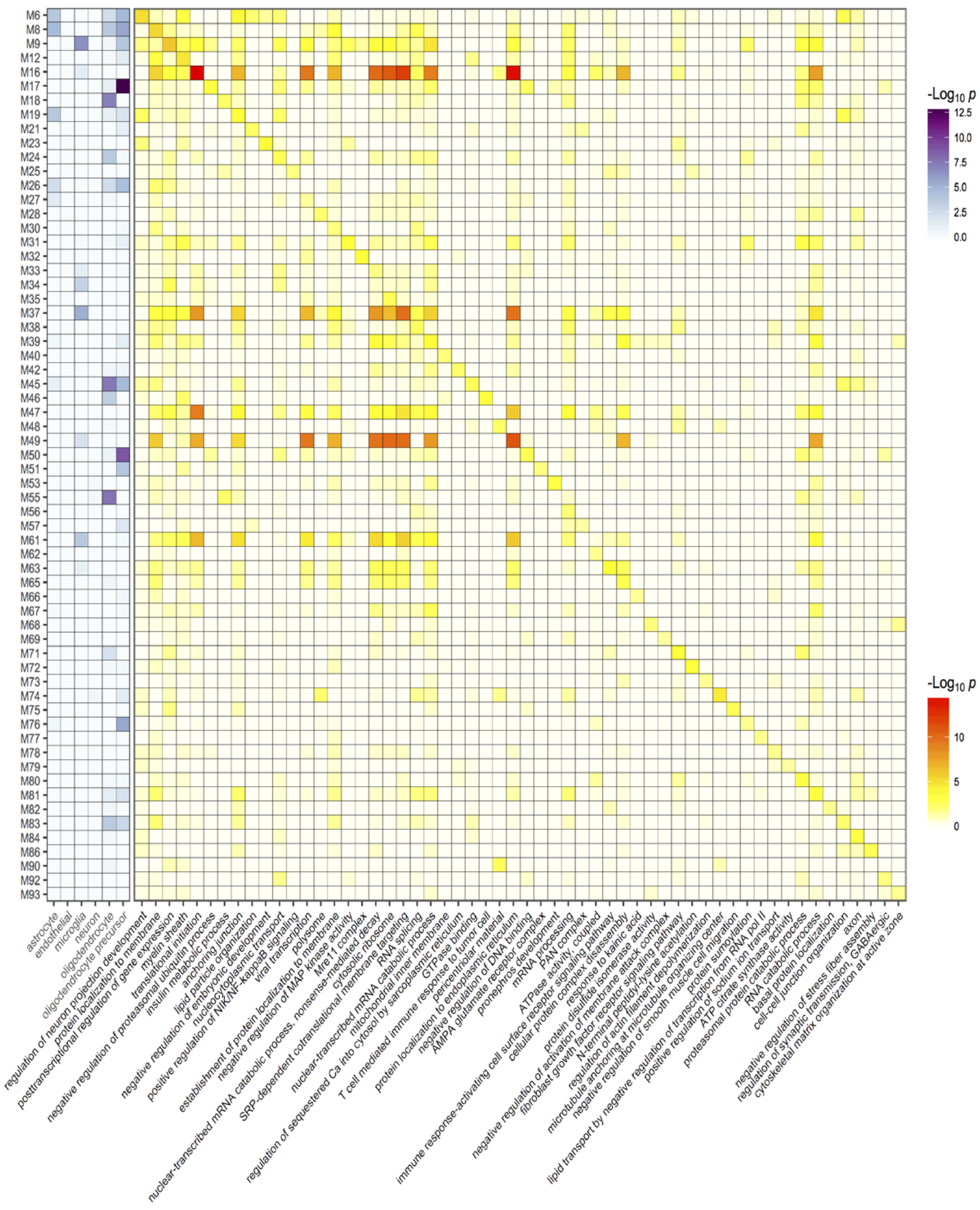

M6
M8
M9
M12
M16
M17
M18
M19
M21
M23
M24
M25
M26
M27
M28
M30
M31
M32
M33
M34
M35
M37
M38
M39
M40
M42
M45
M46
M47
M48
M49
M50
M51
M53
M55
M56
M57
M61
M62
M63
M65
M66
M67
M68
M69
M71
M72
M73
M74
M75
M76
M77
M78
M79
M80
M81
M82
M83
M84
M86
M90
M92
M93
-Log10 p
12.5
10.0
7.5
5.0
2.5
0.0
-Log10 p
10
5
0
astrocyte
endothelial
microglia
neuron
oligodendrocyte
oligodendrocyte precursor
regulation of neuron projection development
protein localization to membrane
posttranscriptional regulation of gene expression
myelin sheath
translational initiation
negative regulation of proteasomal ubiquitin process
insulin metabolic process
anchoring junction
lipid particle organization
negative regulation of embryonic development
nucleocytoplasmic transport
positive regulation of NIK/NF-kappaB signaling
viral transcription
polysome
establishment of protein localization to membrane
negative regulation of MAP kinase activity
Mre11 complex
nuclear-transcribed mRNA catabolic process, nonsense-mediated decay
cytosolic ribosome
SRP-dependent cotranslational membrane targeting
RNA splicing
nuclear-transcribed mRNA catabolic process
mitochondrial inner membrane
regulation of sequestered Ca into cytosol by sarcoplasmic reticulum
GTPase binding
T cell mediated immune response to tumor cell
pericentriolar material
protein localization to endoplasmic reticulum
negative regulation of DNA binding
AMPA glutamate receptor complex
pronephros development
mRNA processing
PAN complex
ATPase activity, coupled
immune response-activating cell surface receptor signaling pathway
cellular protein complex disassembly
response to kainic acid
protein disulfide isomerase activity
negative regulation of activation of membrane attack complex
fibroblast growth factor receptor signaling pathway
N-terminal peptidyl-lysine acetylation
regulation of actin filament depolymerization
microtubule anchoring at microtubule organizing center
negative regulation of smooth muscle cell migration
lipid transport by negative regulation of transcription from RNA pol II
protein sumoylation
positive regulation of sodium ion transport
ATP citrate synthase activity
proteasomal protein catabolic process
RNA catabolic process
basal protein localization
cell-cell junction organization
axon
negative regulation of stress fiber assembly
regulation of synaptic transmission, GABAergic
cytoskeletal matrix organization at active zone

Supplementary Figure 18. Enrichments of MEGENA modules from human oligodendrocyte precursor cells in brain cell type signatures and GO terms. The left panel shows the enrichment (Benjamini-Hochberg adjusted -$\log_{10}$ p-value) of each module in the top 500 most cell type-specific genes for each cell type in humans that we identified in this manuscript. The right panel shows the most significantly enriched GO term (Benjamini-Hochberg adjusted -$\log_{10}$ p-value) for the genes in each of the corresponding modules, along with the GO enrichment of that same GO term in all the other modules.

References

1. Dobin, A. *et al.* STAR: ultrafast universal RNA-seq aligner. *Bioinformatics* **29**, 15-21, doi:10.1093/bioinformatics/bts635 (2012).
2. Liao, Y., Smyth, G. K. & Shi, W. featureCounts: an efficient general purpose program for assigning sequence reads to genomic features. *Bioinformatics* **30**, 923-930, doi:10.1093/bioinformatics/btt656 (2014).
3. Robinson, M. D., McCarthy, D. J. & Smyth, G. K. edgeR: a Bioconductor package for differential expression analysis of digital gene expression data. *Bioinformatics (Oxford, England)* **26**, 139-140, doi:10.1093/bioinformatics/btp616 (2010).
4. McCarthy, D. J., Campbell, K. R., Lun, A. T. L. & Wills, Q. F. scater: pre-processing, quality control, normalisation and visualisation of single-cell RNA-seq data in R. *bioRxiv*, doi:10.1101/069633 (2016).
5. Bullard, J. H. *et al.* Evaluation of statistical methods for normalization and differential expression in mRNA-Seq experiments. *BMC Bioinformatics* **11**, 94, doi:10.1186/1471-2105-11-94 (2010).
6. Durinck, S. *et al.* BioMart and Bioconductor: a powerful link between biological databases and microarray data analysis. *Bioinformatics (Oxford, England)* **21**, 3439-3440, doi:10.1093/bioinformatics/bti525 (2005).
7. Miller, J. A. *et al.* Strategies for aggregating gene expression data: the collapseRows R function. *BMC bioinformatics* **12**, 322, doi:10.1186/1471-2105-12-322 (2011).
8. Chikina, M., Zaslavsky, E. & Sealfon, S. C. CellCODE: a robust latent variable approach to differential expression analysis for heterogeneous cell populations. *Bioinformatics* **31**, 1584-1591, doi:10.1093/bioinformatics/btv015 (2015).
9. Miller, J. A. *et al.* Neuropathological and transcriptomic characteristics of the aged brain. *Elife* **6**, doi:10.7554/eLife.31126 (2017).
10. Kozlenkov, A. *et al.* Substantial DNA methylation differences between two major neuronal subtypes in human brain. *Nucleic acids research* **44**, 2593-2612, doi:10.1093/nar/gkv1304 (2016).
11. Buenrostro, J. D., Giresi, P. G., Zaba, L. C., Chang, H. Y. & Greenleaf, W. J. Transposition of native chromatin for fast and sensitive epigenomic profiling of open chromatin, DNA-binding proteins and nucleosome position. *Nature methods* **10**, 1213-1218, doi:10.1038/nmeth.2688 (2013).
12. McKenzie, A. T., Katsyv, I., Song, W. M., Wang, M. & Zhang, B. DGCA: A comprehensive R package for Differential Gene Correlation Analysis. *BMC Syst Biol* **10**, 106, doi:10.1186/s12918-016-0349-1 (2016).
13. Falcon, S. & Gentleman, R. Using GOstats to test gene lists for GO term association. *Bioinformatics (Oxford, England)* **23**, 257-258, doi:10.1093/bioinformatics/btl567 (2007).
